# Tissue-adhesive hydrogel optical fiber for peripheral optogenetic neuromodulation

**DOI:** 10.1038/s41467-026-74831-1

**Published:** 2026-06-29

**Authors:** Xingmei Chen, Lulu Wang, Chang Wang, Yafei Wang, Liangjie Shan, Yu Xue, Zhongjie Ma, Cunjiang Yu, Yi Lu, Ji Liu

**Affiliations:** 1 Department of Mechanical and Energy Engineering, Southern University of Science and Technology, Shenzhen, China; 2 Materials Research Laboratory, University of Illinois, Urbana-Champaign, Urbana, IL USA; 3 State Key Laboratory of Brain Cognition and Brain-inspired Intelligence Technology, Shenzhen Institutes of Advanced Technology (SIAT), Chinese Academy of Sciences (CAS), Shenzhen, China; 4 Shenzhen-Hong Kong Institute of Brain Science, Shenzhen Institutes of Advanced Technology (SIAT), Chinese Academy of Sciences (CAS), Shenzhen, China; 5 Department of Electrical and Computer Engineering, University of Illinois, Urbana-Champaign, Urbana, IL USA; 6 Department of Materials Science and Engineering, University of Illinois, Urbana-Champaign, Urbana, IL USA; 7 Department of Mechanical Science and Engineering, University of Illinois, Urbana-Champaign, Urbana, IL USA; 8 Department of Bioengineering, University of Illinois, Urbana-Champaign, Urbana, IL USA

**Keywords:** Electronic devices, Biomedical engineering

## Abstract

While hydrogel optical fibers hold promises for visceral peripheral optogenetics, their utility is limited by poor tissue adhesion, unstable light delivery, and micromisplacement under physiological motion, leading to off-target illumination. To address these challenges, we developed tissue-adhesive hydrogel optical fibers (TAHOFs) integrating a poly(HEMA) light-guiding core (*n*_core_ = 1.429 ± 0.004) with a bioadhesive cladding (*n*_cladding_ = 1.343 ± 0.002), achieving dual functionality through refractive index contrast (Δ*n* = 0.086) and robust tissue integration (11.5 ± 1.8 kPa). This architecture enables efficient optical confinement with low propagation loss (0.534 ± 0.092 dB/cm) while maintaining spatial targeting fidelity under 30% tensile strain. Pancreatic implantation in freely moving ChAT-ChR2 mice demonstrated precise vagal fiber activation, effectively triggering insulin secretion through mechanically and optically stable light delivery. Integrated with subcutaneous continuous glucose monitoring, TAHOFs enabled 3-day glycemic control in diabetic models, showing stable blood glucose reduction and real-time regulation. Chronic implantation demonstrated that TAHOF supports stable in vivo adhesion on the pancreas while maintaining optogenetic function for up to 14 days. The TAHOF uniting high optical efficiency, mechanical compliance, and biological integration, offering an application-specific design strategy for optogenetic neuromodulation in moving animals, particularly mechanically dynamic and anatomically complex organs.

## Abstract (web summary)

Precise optogenetic neuromodulation is challenged by body motion. Here, the authors develop a tissue-adhesive hydrogel optical fiber that enables stable pancreatic vagal stimulation in freely moving mice, supporting insulin secretion and glycemic modulation over 14 days.

## Introduction

Optogenetics has transformed biological control by enabling millisecond-precise manipulation of specific neural populations through genetically encoded light-sensitive ion channels^1,2^. Its therapeutic extension to neural circuit interrogation could further facilitate therapeutic modulation of metabolic, immune, and secretory functions through targeted neuromodulation^3–6^. However, adapting these optogenetic tools to dynamically moving internal organs (e.g., heart, pancreas or intestines) requires optical interfaces that deliver high-fidelity light while maintaining concurrent deformation with the organs^3,7–9^. Conventional silica/polymer fibers, while effective for static neural environments, exhibit severe mechanical mismatch (Young’s modulus: GPa vs. kPa for tissues) when interfacing with deforming organs undergoing physiological strains (e.g., 40% peristaltic deformation)^7,10–12^. Such mechanical mismatch causes interfacial decoupling, chronic inflammation, and spatial mis-targeting even during physiological movements like respiration or posture changes, fundamentally limiting their utility in visceral optogenetics.

Recent advances in hydrogel-based optical fibers offer a promising solution by providing tissue-like softness and hydrated architectures that mitigate foreign body responses^11,13–18^. Core-cladding designs, employing the step-index profiles for total internal reflection, could achieve low light loss (0.3–2 dB/cm) for optogenetic neuromodulation^14,15,18,19^. However, visceral optogenetics is fundamentally constrained by motion-induced optical decoupling, a critical limitation arising from cyclic physiological deformations that disrupt conformal integration and attenuate light delivery efficiency in dynamic environments. Existing strategies relying on mechanical fixtures (e.g., nerve cuffs) are ineffective for irregular and ultrasoft visceral organs like the pancreas, leaving the development of flexible, durable, and conformable optical interfaces as a critical challenge in visceral peripheral optogenetics^11,20–23^. To overcome these limitations, the ideal optical fiber for optogenetic neuromodulation of dynamic organs should synergistically possess (i) strain-resilient light confinement capable of withstanding dynamic organ deformations, (ii) persistent wet-tissue integration without compromising optical transmission, and (iii) 3D conformability to complex and dynamic organ surfaces.

**Fig. 1:**
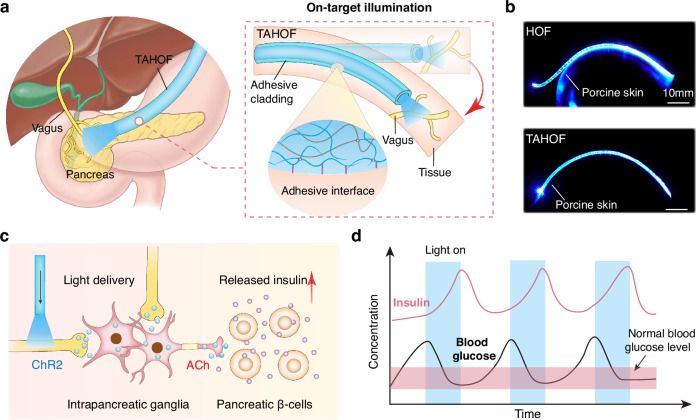
Tissue-adhesive hydrogel optical fibers (TAHOFs) for peripheral optogenetic neuromodulation of pancreatic vagal innervation. **a** Schematic illustration of TAHOFs implantation for light delivery to the vagal nerve in the pancreas, enabling optogenetic glycemia modulation. Inset: TAHOF architecture, comprising an optical hydrogel core and a tissue-adhesive cladding that forms robust biointerface with pancreatic tissue for stable illumination. **b** Optical images showing light propagation through TAHOF adhered to porcine skin under ~90° bending deformation, with core-only poly(2-hydroxyethyl methacrylate) (PHEMA) fiber (HOF) as control. Scale bar: 10 mm. **c** Optogenetic activation of ChR2-expressing cholinergic vagal fibers via TAHOF, triggering acetylcholine (ACh) release from vagal nerve terminals and intrapancreatic ganglia to stimulate insulin secretion in β cells. **d** Schematic of blood glucose regulation mediated by TAHOF-delivered light stimulation. The organs schematic in (**a**) was adapted from the NIH/NIDDK media library (https://www.niddk.nih.gov/news/media-library/23139).

Building on recent advances in hydrogel optical fiber design and hydrogel chemistries, we report a tissue-adhesive hydrogel optical fiber (TAHOF) as a light-delivery tool for peripheral optogenetics, enabling on-target optogenetic neuromodulation of visceral nerves in freely moving animals. The single-fiber hydrogel architecture integrates efficient optical transmission, strong tissue adhesion, and mechanical compliance to achieve high-fidelity modulation (Fig. 1). Our TAHOF is composed of a poly(2-hydroxyethyl methacrylate) (PHEMA) optical core (*n*_core_ = 1.429 ± 0.004) with a tissue-adhesive cladding (*n*_cladding_ = 1.343 ± 0.002), which simultaneously provides tissue adhesion and the refractive-index contrast required for light confinement. The reversible tissue adhesion is achieved through hydrogen bonding and electrostatic interactions, and strain-adaptive compliance matching soft organ mechanics. Fabricated through templated polymerization, TAHOFs exhibit 70% light transmission under 30% tensile strain while maintaining 11.5 ± 1.8 kPa interfacial adhesion on hydrated tissues. In vivo validation demonstrates long-term mechanical and optical stability on the dynamic surface of the pancreas, enabling precise optogenetic activation of pancreatic vagal innervation in freely moving animal models and real-time glycemic regulation when combined with continuous glucose biosensing. TAHOFs maintained continuous glycemic regulation in diabetic models over 3 days, a 3× durability improvement compared to non-adhesive counterparts, which failed within one day. By reconciling previously incompatible requirements for optical efficiency, mechanical resilience, and tissue integration, this TAHOF establishes a robust route for spatiotemporally precise peripheral neuromodulation in dynamic internal organs, particularly of moving animals.

## Results

### Design and fabrication of TAHOFs

**Fig. 2:**
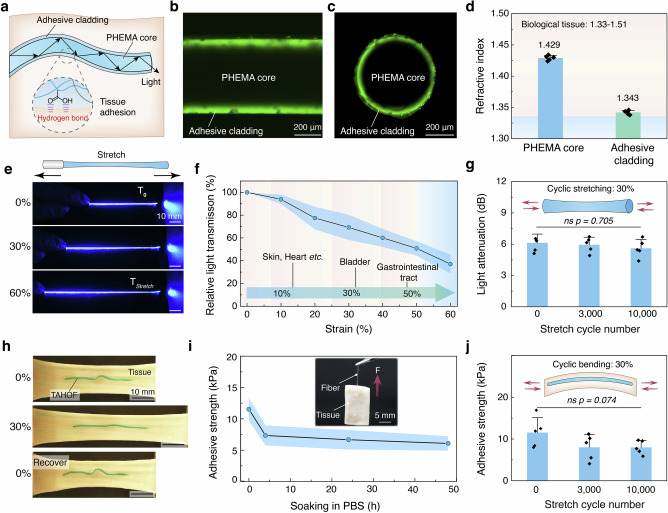
Optical and tissue-adhesive properties of TAHOFs. **a** Schematic of the core-cladding structure, featuring a PHEMA core and a PAA-PVA tissue-adhesive cladding. The step-index configuration ensures total internal reflection, minimizing light leakage in biological environments, while the cladding enables robust bioadhesion for stable optical delivery. **b**, **c** Cross-sectional fluorescence images of the fiber, with the FITC-labeled adhesive cladding highlighting the core-cladding architecture. Scale bar, 200 μm. **d** Refractive index measurements of the PHEMA core and tissue-adhesive hydrogel cladding. **e** Light propagation through the TAHOF under tensile strains (0%, 30%, 60%), with the distal end fixed by transparent adhesive tape. Scale bar, 10 mm. **f** Normalized light transmission intensity versus tensile strain. Inset: representative strain range for biological organs and tissues. **g** Light attenuation profiles during cyclic tensile loading (30% strain) at 0, 3000, and 10,000 cycles (fiber dimensions: 5 cm length, 650 μm diameter). **h** Conformal deformation of a tissue-TAHOF joint adhered to porcine tissue under 30% strain. Scale bar, 10 mm. **i** Adhesive strength between the cladding and porcine skin measured by lap shear tests over 48 hours in PBS. Inset: stability of the adhesive interface between TAHOF and porcine skin. Scale bar, 5 mm. **j** Adhesive strength after 10,000 bending cycles (30% strain). Fiber dimensions: 5 cm length, 650 μm diameter. Data in (**d**, **f**, **g**, and **j**) are presented as mea*n*± SD, whereas data in (**i**) are presented mean ± SE (*n* = 5 independent experiment samples). Statistical significance was determined by one-way ANOVA; *p* > 0.05 was considered not significant.

The TAHOF integrates a poly(HEMA) optical core (550 μm diameter, RI = 1.429 ± 0.004) with a tissue-adhesive poly(acrylic acid)/polyvinyl alcohol (PAA-PVA) cladding (50 μm thickness, RI = 1.343 ± 0.002) to enable efficient and localized light delivery to pancreatic tissue (Fig. 2a and Supplementary Fig. 1). The fibers are fabricated via sequential photopolymerization and chemical crosslinking in silicone molds (Fig. 2b, c, and Supplementary Fig. 2), and coupled to a ceramic ferrule through a swelling-assisted fit, where the dry fiber (528 ± 16 μm) expands to 666 ± 14 μm after 4 h in PBS, forming a conformal interface within a 650 μm-inner-diameter ferrule (Suppl. Fig. 3). The resulting step-index architecture enables efficient optical confinement via total internal reflection, with low propagation loss (0.534 ± 0.092 dB/cm at 470 nm, Fig. 2d and Supplementary Figs. 4, 5). Although core-only fibers exhibited marginally lower propagation loss in air (0.456 ± 0.069 dB/cm), the lower-RI cladding plays a crucial role in suppressing optical leakage when the fibers are implanted into biological tissues with higher refractive indices (RI = 1.33–1.51)^19,24,25^. Moreover, as the wavelength increased from 470 to 644 nm, the light attenuation decreased from 0.534 ± 0.092 dB/cm to 0.452 ± 0.067 dB/cm, indicating wavelength-dependent attenuation in the TAHOF waveguide and compatibility with longer wavelengths (Suppl. Fig. 5). FDTD simulations performed under a conservative condition (*n*_tissue_ = 1.50), together with empirical measurements, confirmed that the cladding enhances optical confinement (Supplementary Figs. 6–8). TAHOFs deliver 70.4 ± 9.7% of in-air light intensity over 6 cm on porcine skin, a 3.3-fold improvement over core-only fibers (21.9 ± 6.2%, Suppl. Fig. 7), highlighting enhanced confinement at the tissue-fiber interface. Notably, only ~30% of the optical power was lost over the 6 cm propagation path in tissue, and this leakage was distributed along the fiber length rather than concentrated at a single site. With further attenuation by tissue scattering and absorption, the resulting side-leakage intensity is unlikely to be sufficient to induce unintended optogenetic activation of surrounding organs.

Physiological motion and organ expansion were simulated by applying tensile strains ranging from 10% to 60%, spanning the range observed in typical in vivo deformations^10,11,26^. Under these conditions, TAHOFs exhibited high mechanical and optical resilience, retaining 69.2 ± 11.0% of normalized light output at 30% strain, representative of physiological extremes in visceral organs (Fig. 2e, f and Suppl. Movie 1), and outperforming conventional fibers such as silica (fracture at ~1% strain) and PDMS (failure at ~20%)^11,27^. Under repeated loading, the normalized transmitted intensity remained above 95% of the initial (pre-cycling) value after 10,000 cycles, while optical attenuation showed minimal change (6.1 ± 0.84 dB to 5.6 ± 0.86 dB, Fig. 2g and Suppl. Fig. 9), and stable performance was maintained after 30-day physiological immersion (Suppl. Fig. 10). Mechanically, the fiber architecture matched tissue compliance (Young’s modulus of 428 ± 117 kPa, Suppl. Fig. 11), approximately 4–5 orders lower than that of silica (∼50 GPa) or polycarbonate (PC, ∼3 GPa) fibers^13^, with robust tensile strength (445 ± 64 kPa) and stretchability (176 ± 24%). Thereby, such design aligns with soft tissues like muscle (∼100 kPa) and pancreas (∼5 kPa)^28,29^. Additionally, cyclic testing confirmed sustained mechanical integrity, with consistent 30% maximum strain tolerance over 10,000 cycles and stable post-hydration performance (Suppl. Fig. 12), thereby mitigating chronic inflammation risks associated with traditional rigid implants.

Despite the mechanical compliance, hydrogel fibers without adhesive cladding are prone to dislocate under dynamic loads, leading to off-target illumination (Fig. 1b). To address this, tough and tissue-adhesive PAA-PVA cladding leverages hydrogen bonding and electrostatic interactions for robust tissue adhesion^30^. Bioadhesion strength of 11.5 ± 1.8 kPa was quantified in porcine skin, surpassing commercial fibrin glue ( < 5 kPa)^31^, while retaining 6.1 ± 1.2 kPa after 48 h hydration (Fig. 2i and Suppl. Figs. 13, 14). Under simulated physiological motion, involving 10,000 cycles of 30% strain, interfacial adhesion maintained >70% integrity, ensuring stable light coupling even during 90° bending (Fig. 1b, Fig. 2h, j, and Suppl. Movie 2). This durability originates from PAA-PVA interpenetration at the core-cladding interface, enhancing the interfacial toughness by 3.6× (Suppl. Fig. 15). Integrated design of the TAHOF effectively eliminated off-target illumination observed in non-adhesive hydrogel optical fibers, thus enabling precise visceral peripheral optogenetic activation and neuromodulation under dynamic physiological conditions.

### Biomechanical integration and strain-resilient optical delivery

**Fig. 3:**
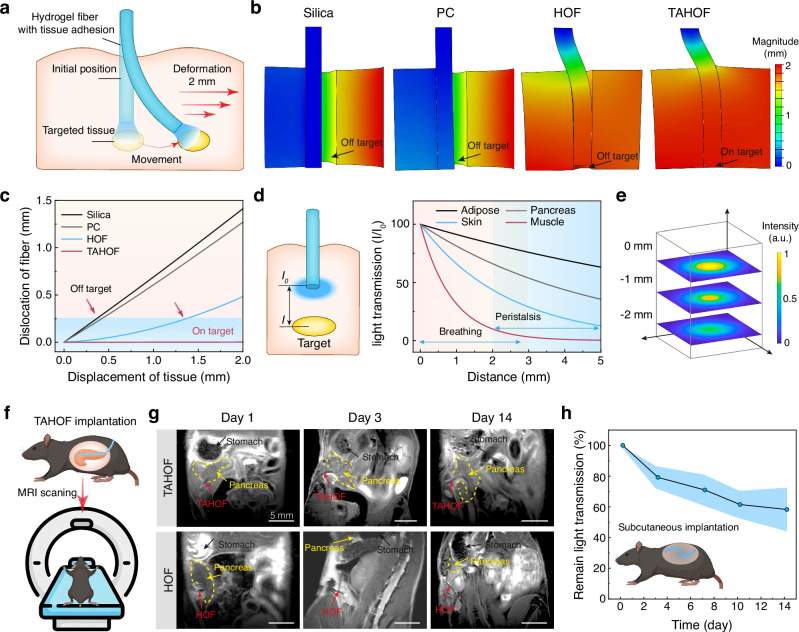
TAHOFs enable targeted optical illumination in dynamically deforming tissues. **a** Finite element analysis (FEA) model simulating TAHOF implantation into biological tissue subjected to physiological displacement (up to 2 mm), mimicking breathing, peristalsis, or organ deformation. **b** Magnitude profiles of silica, polycarbonate (PC), hydrogel optical fibers without adhesive cladding (HOF), and TAHOF implanted into tissue under 2 mm displacement. **c** Relative displacement of the implanted fibers (silica, PC, HOF, TAHOF) versus tissue motion (0−2 mm). **d** Simulated optical attenuation of 470 nm light in adipose, skin, pancreas, and muscle tissues, based on their respective absorption coefficients (adipose: 0.04 mm⁻¹; skin: 0.18 mm⁻¹; pancreas: 0.09 mm⁻¹; muscle: 0.5 mm⁻¹)^41^. **e** Simulated spatial distribution of 470 nm light in pancreatic tissue at 1 mm and 2 mm from the emission point. **f** Schematic of TAHOF implantation on the mouse pancreas and longitudinal in vivo tracking via MRI. **g** Representative MRI images at day 1, day 3, and day 14, showing stable positioning of TAHOF (red arrows) relative to the pancreas (yellow arrows; dashed outlines), with the stomach indicated by black arrows; HOF serves as control. **h** Relative light transmission of TAHOFs retrieved after subcutaneous implantation for 3, 7, 10, and 14 days. Data in h are presented as mea*n*± SD (*n* = 5 independent experiment samples) The mouse model schematic of (**f**, **h**) are created in BioRender. Chen, X. (2026) https://BioRender.com/9g29yc6.

To assess TAHOF’s ability to maintain targeted illumination under mechanical deformation, we developed a 3D finite element analysis (FEA) model simulating physiological motion (e.g., respiration, peristalsis, and muscular contraction, see *Methods* for details)^32,33^. The model mimicked TAHOF implantation in muscle-like tissue (Young’s modulus: 0.12 MPa; Poisson’s ratio: 0.5), with fiber dimensions of 650 μm diameter and 20 mm length (Fig. 3a-c, Suppl. Table 1). FEA revealed exceptional positional fidelity ( <0.1% tip deviation under 2 mm lateral deformation), outperforming non-adhesive hydrogel fiber (HOF, 0.48 mm/24% mismatch) and rigid counterparts ( > 60% displacement) due to synchronized tissue-fiber motion (Fig. 3b, c). Such misalignment, caused by their low strain tolerance ( < 1%)^11^, resulted in substantial off-target illumination. The adhesive cladding’s strain accommodation reduced interfacial von Mises stress by 8× versus silica fibers (Supplementary Fig. 16), while wavelength-dependent attenuation profiles highlighted organ-specific design needs (70% vs. 20% light loss per mm in muscle vs. pancreas at 470 nm; Fig. 3d, e). This synergy enabled TAHOFs to sustain optical coupling despite scattering in dynamic environments.

Experimental validation involved adhering TAHOF to subcutaneous tissue in C57BL/6 J mice to visualize the in vivo optical delivery (Supplementary Fig. 17). Supplementary Fig. 17b, c, and Supplementary Movie 3 confirmed motion-adaptive light delivery under 30% tensile strain, with < 30% intensity loss during deformation and full recovery upon relaxation, consistent with in vitro data (Fig. 2f). Cladding-deficient HOF exhibited complete optical decoupling at 5 mm displacement (Supplementary Fig. 17d–f), underscoring the adhesive’s critical role. To further evaluate positional stability under physiological conditions, we performed longitudinal in vivo tracking using high-field (9.4 T) MRI following pancreatic implantation for up to 14 days (Fig. 3f). While both TAHOFs and control HOFs were correctly positioned at day 1, the HOF control showed pronounced displacement by day 3 and continued migration toward adjacent regions (e.g., kidney) by day 14, whereas TAHOFs remained stably anchored at the implantation site and maintained intimate contact with the pancreas throughout (Fig. 3g).

Post-mortem examination further confirmed persistent adhesion of TAHOFs to the pancreatic surface (Suppl. Fig. 18), consistent with FEA predictions. Meanwhile, optical transmission exhibited a gradual decline after implantation, decreasing to 79.2 ± 7.1% at day 3 and 58.3 ± 14.1% at day 14 relative to the pre-implantation baseline (defined as 100%) (Fig. 3h), indicating progressive attenuation rather than abrupt failure. Collectively, these results demonstrate that TAHOFs uniquely combine high stretchability (170 ± 25%, Suppl. Fig. 11c) with stable, submillimeter targeting in dynamically deforming visceral organs, thereby overcoming a key limitation in peripheral optogenetic neuromodulation.

### Neuromodulatory control of blood glucose via the vagus-pancreas axis enabled by TAHOFs

**Fig. 4:**
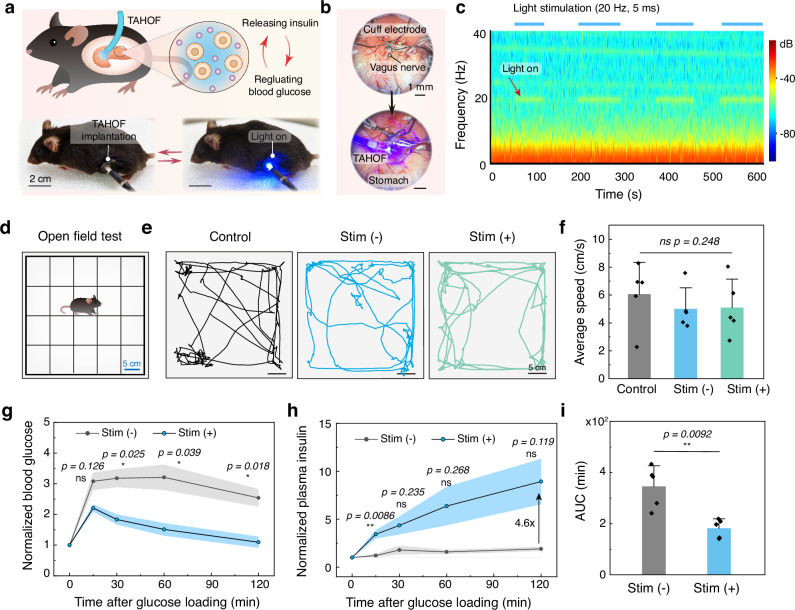
Optogenetic stimulation of the pancreatic vagal innervation using TAHOFs for insulin secretion modulation. **a** Schematic of optical activation of the pancreatic vagus nerve via implanted TAHOF in ChAT-ChR2 transgenic mice (top) and photograph of an implanted mouse (bottom). Scale bar: 2 cm. **b** Experimental setup for simultaneous optical stimulation and electrophysiological recording from the isolated subdiaphragmatic vagus nerve. Blue light was delivered through the TAHOF, while local field potentials (LFPs) were recorded using an adjacent cuff electrode. Scale bar, 1 mm. **c** Spectrogram of vagal LFPs during optical stimulation (470 nm, 20 Hz, 5 ms), showing robust time-locked neural responses. **d** Open field test (OFT) setup for assessing motor behavior in implanted mice. **e** Representative OFT movement traces before (Stim(−)) and during (Stim(+)) optical stimulation, with sham controls. **f** Quantification of the average locomotion speed of mice during 3 min of optical stimulation in the OFT (*n* = 5 mice). **g**, **h** Normalized blood glucose levels (**g**) and plasma insulin (**h**) during intraperitoneal glucose tolerance tests (2 g/kg) with optogenetic stimulation. **i** Area under the curve (AUC) for glucose levels in (**g**) (*n* = 5 mice). Data in (**f**,** i**) are means ± SD, *n* = 5 mice; Data in (**g**,** h**) are means ± SE, *n* = 5 mice. Statistical significance and *p*-values were determined using two-way repeated measures ANOVA followed by Bonferroni post hoc test (**g**, **h**), two-sided t-tests (**i**) and one-way ANOVA (**f**), respectively. ns not significant; *p < 0.05, **p < 0.01. Groups are denoted as Stim(−)/Stim(+). **g** 15 mi*n *ns. *p* = 0.126, 30 min *p = 0.025, 60 min *p = 0.039, 120 min *p = 0.018. **h** 15 min **p = 0.0086, 30 mi*n *ns. *p* = 0.235, 60 mi*n *ns. *p* = 0.268, 120 mi*n *ns. *p* = 0.119. **i** **p = 0.0092.

The vagus nerve (VN) serves as a critical bidirectional conduit between the brain and diverse visceral organs, including the pancreas^34,35^, with efferent cholinergic fibers releasing acetylcholine (ACh) to regulate target physiology^6,36^. To achieve cell-type-specific stimulation, we generated ChAT-ChR2 transgenic mice expressing the blue light-sensitive channel ChR2 in cholinergic vagal axons innervating the pancreas (Suppl. Fig. 19). Optical stimulation (470 nm, 20 Hz, 5 ms) was delivered to the subdiaphragmatic vagus nerve via implanted TAHOFs, while local field potentials (LFPs) were recorded using a nearby cuff electrode. This configuration produced robust, time-locked responses at 20 Hz (Fig. 4b, c), with a steady increase in LFP power during stimulation, indicating reliable and sustained activation of vagal fibers. Importantly, no time-locked responses were observed in control conditions, including blue-light stimulation in ChR2-negative (ChR2(−)) mice and yellow-light stimulation in ChAT-ChR2 mice (Suppl. Fig. 20), confirming that the recorded signals arise from ChR2-dependent neural activation rather than optical or photoelectric artifacts.

Given the VN’s broad influence on motor and cardiovascular function^37^, we assessed whether selective stimulation of its pancreatic branches altered locomotor activity or heart rate. We positioned TAHOF adjacent to pancreatic vagal branches, leveraging their adhesive hydrogel cladding for suture-free placement (Fig. 4a, Suppl. Fig. 21 and Suppl. Movie 4). Open Field Test (OFT) analysis revealed no significant differences in locomotor activity between sham, unstimulated, and stimulated groups (*p* = 0.248; Fig. 4d−f), and heart rate remained stable ( ~ 300 bpm) during stimulation (Supplementary Fig. 22a, b). Histological and immunological analyses confirmed good biocompatibility: Histological and immunological analyses showed no necrosis or inflammation in major organs after 3 days of optical stimulation (Supplementary Figs. 22c and 23), and fibrotic encapsulation around TAHOF remained minimal throughout the 14-day post-implantation period (Supplementary Fig. 24). CD68⁺ macrophage infiltration in TAHOF-implanted tissues (6.1 ± 1.1%) was comparable to that in sham controls (7.8 ± 3.7%; *p* = 0.356, not significant; Supplementary Fig. 25), further supported TAHOF’s suitability for physiological studies and therapeutic applications.

We next evaluated the physiological impact of pancreatic cholinergic vagal activation on glucose homeostasis. Prior studies suggest that activation of cholinergic vagal fibers innervating the pancreas enhances β-cell insulin secretion and local perfusion, leading to reduced blood glucose^4,38^. In fasted ChAT-ChR2 mice, intraperitoneal glucose tolerance tests (GTTs) revealed that optical stimulation (470 nm, 20 Hz, 5 ms) during glucose challenge (2 g/kg) attenuated the rise in blood glucose by 2.2-fold (from 9.2 ± 0.53 to 20.3 ± 1.71 mmol/L), in contrast to 3.1-fold in the absence of stimulation (from 7.0 ± 1.08 to 20.5 ± 1.25 mmol/L, Fig. 4g and Supplementary Fig. 26a). The Stim (+) group exhibited 2.8-fold higher plasma insulin at 15 minutes (1.34 ± 0.08 ng/mL vs. 0.48 ± 0.05 ng/mL) and a 44.3 ± 7.8% reduction in glucose AUC (Fig. 4h, i, and Supplementary Fig. 26b, c). Notably, insulin levels remained elevated for 120 min ( ~ 7.35-fold over baseline *vs*. ~1.91-fold in controls). We further evaluated stimulation frequency dependence and found that optical activation at 10, 20, and 30 Hz all reduced blood glucose levels between 10- and 40 min post-injection (Supplementary Fig. 27), indicating that vagal activation is sufficient to drive glucose-lowering effects. Notably, 20 Hz produced a stronger response than 10 Hz, while increasing the frequency to 30 Hz did not yield additional benefit, supporting 20 Hz as an effective stimulation parameter.

### TAHOFs-mediated real-time optogenetic regulation of glycemia in diabetic mice models

**Fig. 5:**
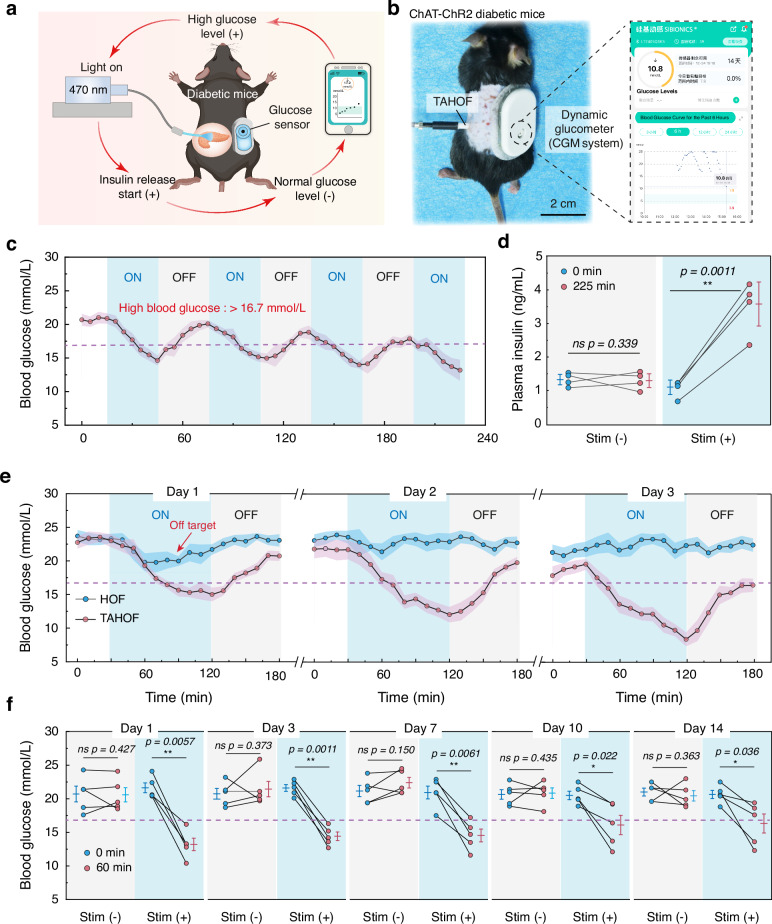
Real-time glycemic modulation via optogenetic stimulation using TAHOFs in diabetic mice. **a** Integrated system combining TAHOF and a continuous glucose monitoring (CGM) sensor for real-time blood glucose regulation in STZ-induced ChAT-ChR2 diabetic mice. **b** Photograph of an implanted mouse with TAHOF and CGM system (SIBIONICS^®^), showing wireless glucose monitoring via smartphone interface (dashed box). Scale bar: 2 cm. **c** Blood glucose changes over 225 minutes in diabetic mice with or without optical stimulation (470 nm, 20 Hz, 5 ms) delivered through TAHOF, recorded via CGM. **d** Plasma insulin levels at 225 min, showing significant increase in stimulated mice versus controls. **e** Sustained glycemic regulation over 3 days with daily 90 min optical stimulation via TAHOF, compared to fibers lacking adhesive cladding (HOF). **f** Changes in blood glucose of individual treated mice before and after 60 min of optical stimulation at day 1, 3, 7, 10, and 14 after TAHOF implantation. Data in (**c**, **e**) are presented as mea*n*± SE, *n*= 4 mice per group; data in (**d**) are presented as mea*n*± SD, *n* = 4 mice per group; and data in (**f**) are presented as mean ± SE, *n *= 5 mice per group. Statistical analyses in (**d**) and (**f**) were performed using two-tailed paired Student’s t-tests. ns, not significant; *p < 0.05; **p < 0.01; ***p < 0.001. Groups are denoted as Stim(−)/Stim(+). **d** Stim(−) ns. *p* = 0.339, Stim(+) **p = 0.0011. **d** Day1/Stim(−) ns. *p* = 0.427, Day1/Stim (+) **p = 0.0057, Day3/Stim(−) ns. *p* = 0.373, Day3/Stim (+) **p = 0.0011, Day7/Stim(−) ns. *p* = 0.150, Day7/Stim (+) **p = 0.0061, Day10/Stim(−) ns. *p* = 0.435, Day10/Stim (+) **p = 0.022, Day14/Stim(−) ns. *p* = 0.363, Day14/Stim (+) *p = 0.036. The mouse model schematic of (**a**) is created in BioRender. Chen, X. (2026) https://BioRender.com/9g29yc6.

Building on evidence that optogenetic vagal stimulation enhances glucose tolerance and insulin secretion in ChAT-ChR2 mice, we further evaluated TAHOFs’ therapeutic potential in STZ-induced diabetic mice (60 mg/kg)^4,39^. With TAHOFs implanted onto the pancreatic surface, real-time glucose monitoring was conducted via a continuous glucose monitoring (CGM, SiBiONICS^®^) system (Fig. 5a, b, and Supplementary Fig. 28), facilitating the temporal resolution of glycemic changes during neuromodulation. Cyclic optogenetic stimulation (470 nm, 20 Hz, 5 ms; 30 min intervals) induced rapid and reversible glucose reductions from ~20.5 ± 0.9 to 16.3 ± 0.8 mmol/L, accompanied by a 3.3-fold increase in plasma insulin (1.1 ± 0.22 to 3.6 ± 0.65 ng/mL; Fig. 5c, d). This rapid and reversible glucose-lowering underscores the temporal specificity of neuromodulation over pharmacological agents. Additionally, the rebound during stimulation pauses highlights the need for sustained intervention to compensate for diminished basal insulin secretion, while repeated activation reliably restored glycemic stability across multiple cycles. In contrast, glucose levels in unstimulated diabetic controls remained elevated ( ~ 20-24 mmol/L, mean of 4 mice) throughout the recording period (Supplementary Fig. 29).

Extended daily stimulation over 3 days further reduced basal glucose levels from 20–23 mmol/L to 8–11 mmol/L (*n* = 4; Fig. 5e), suggesting sustained improvement in glucose homeostasis beyond acute insulin release, potentially reflecting enhanced β-cell responsiveness or islet functional adaptation^4,40^. Notably, the adhesive cladding was critical for maintaining tissue contact and positional stability throughout the 3-day period, uninterrupted delivery of light to the pancreatic innervation. In contrast, non-adhesive HOFs exhibited only transient glucose reduction (23.3 ± 0.7 to 19.8 ± 1.3 mmol/L during the first 30 min of stimulation) followed by rapid rebound due to device displacement and loss of targeting fidelity (Fig. 5e). Unstimulated diabetic controls remained persistently hyperglycemic ( ~ 20-23 mmol/L; *n* = 4) over the same period (Supplementary Fig. 30). Moreover, comprehensive evaluations confirmed that TAHOFs implantation caused no mobility impairment (Supplementary Fig. 31 and Supplementary Movie 5) and maintained normal physiological leukocyte profiles (Supplementary Fig. 32). Overall, these results establish TAHOFs as a spatiotemporally precise neuromodulatory interface for diabetes intervention, enabling stable organ-level control of glucose homeostasis.

To further assess long-term functional stability, we conducted a 14-day in vivo validation study. TAHOF-mediated optical stimulation consistently induced significant reductions in blood glucose at all examined time points (post-implantation days 1, 3, 7, 10, and 14), as quantified by tail-vein sampling (Fig. 5f and Suppl. Fig. S33). In each case, glucose levels decreased during stimulation and returned toward baseline after cessation, confirming stimulation-dependent and reversible control of glycemia. Quantitatively, glucose was reduced by ~6–9 mmol/L during early stages (days 1–7) and ~4–5 mmol/L at later stages (days 10 and 14), with all effects remaining statistically significant. Although a gradual reduction in response magnitude was observed over time, the glucose-lowering effect persisted throughout the full 14-day period. This attenuation is consistent with the progressive decrease in optical transmission (Fig. 3h), rather than loss of device functionality or interface failure. Collectively, these results demonstrate that TAHOF enables stable and reliable modulation of pancreatic vagal activity over extended periods, providing a robust platform for spatiotemporal control of glucose homeostasis.

## Discussion

This work introduces TAHOFs as an integrated platform for chronic optogenetic modulation in mechanically dynamic biological systems. By co-engineering optical confinement and interfacial adhesion within a single fiber architecture, TAHOFs resolve the long-standing trade-off between light-delivery precision and mechanical compatibility in deformable visceral organs. The core-cladding design enables strain-resilient total internal reflection while simultaneously providing conformal tissue integration, thereby minimizing motion-induced optical decoupling in ultrasoft organs such as the pancreas. Consistent with this design principle, MRI tracking confirmed stable in vivo adhesion of TAHOFs on the dynamically moving pancreatic surface for up to 14 days, and optogenetic vagal stimulation remained functionally effective in driving insulin secretion and glycemic regulation in freely moving diabetic mice. Together with the 3-day closed-loop glycemic regulation achieved using continuous glucose monitoring, these results support the feasibility of temporally controlled neuromodulation in dynamic visceral environments.

Beyond the pancreas, TAHOFs establish an application-oriented design framework for soft optical interfaces in anatomically complex and mechanically active biological systems. The key design principles, mechanical compliance, intrinsic tissue adhesion, and deformation-tolerant optical guidance, are broadly applicable to other motion-rich organs, including peristaltic gastrointestinal tissues and pulsatile cardiovascular structures. In support of this generality, we further demonstrated optogenetic modulation of gastric motility in ChAT-ChR2 mice (Suppl. Fig. S34). By replacing rigid external fixation with material-level biointegration, this platform provides a foundation for interrogating peripheral neural circuits in their native physiological context and enables future studies of chronic neuromodulation in dynamically deforming organs.

## Methods

### Materials

Hydroxyethyl methacrylate (HEMA, TCI), 2-hydroxy-2-methylpropiophenone (2-HMP, TCI), and poly(ethylene glycol) diacrylate (PEG(700)-DA, MW = 700 Da, Sigma-Aldrich) were used to synthesize the hydrogel core fiber. To fabricate the tissue-adhesive hydrogel cladding, polyvinyl alcohol (PVA, MW 89–98 kDa, > 99% hydrolyzed, Sigma-Aldrich), acrylic acid (AA, TCI), α-ketoglutarate (α-KA, Adamas), glutaraldehyde (GA, Adamas), and PEG(700)-DA were used to form a chemically crosslinked double-network hydrogel. Blood glucose levels were monitored using either a handheld glucose meter (FreeStyle Libre, Abbott) or a dynamic continuous glucose monitoring (CGM) system (SiBiONICS^®^) via tail vein sampling or subcutaneous implantation in ChAT-ChR2 mice, respectively. PBS buffer purchased from Gibco (USA). Unless otherwise stated, all reagents used for in vivo experiments were obtained from Jackson ImmunoResearch (USA) or Servicebio (Wuhan, China).

### Fabrication of PHEMA hydrogel core fibers

The hydrogel core precursor solution was formulated by combining 80 *wt*% hydroxyethyl methacrylate (HEMA), 1 *wt*% 2-hydroxy-2-methylpropiophenone (2-HMP) photoinitiator, and 2 *wt*% poly(ethylene glycol) diacrylate (PEG(700)-DA) crosslinker in aqueous medium. This mixture was syringe-injected into silicone tube molds (inner diameter: 550 μm; outer diameter: 1200 μm) and photopolymerized under UV irradiation (365 nm, 20 mW cm^−2^) for 45 min. Upon curing, the mold underwent dichloromethane (DCM) immersion (20 min) to facilitate silicone swelling and subsequent core fiber demolding. Residual monomers were removed through sequential solvent treatment: initial ethanol soak (50% *v/v*, 1 h) followed by distilled water immersion (24 h) for solvent exchange.

### Fabrication of the tissue-adhesive hydrogel cladding layer

A dual-network cladding precursor solution was consisted of 10 *wt*% acrylic acid (AA), 0.5 *wt*% α-ketoglutarate (α-KA), 0.5 wt% PEG(700)-DA crosslinker, 10 *wt*% poly(vinyl alcohol) (PVA), and 0.03 *wt*% glutaraldehyde (GA). Additionally, 0.03 M hydrochloric acid (HCl) was added to catalyze the chemical crosslinking of PVA. After vigorous stirring and degassing (2 min), the solution was infused into Tygon^®^ tubing (ID: 760 μm; OD: 2290 μm) housing pre-dried PHEMA core fibers. UV exposure (365 nm, 60 mW cm^−^^2^, 15 min) initiated poly(acrylic acid) (PAA) network formation, followed by 1 h room-temperature incubation to complete PVA chemical crosslinking. Core-cladding fibers were released through DCM-assisted demolding (20 min), air-dried. The resulting tissue-adhesive hydrogel optical fibers (TAHOFs) were then purified by soaking in distilled water for 3 days to remove residual monomers and uncrosslinked polymers.

### Coupling of TAHOF to a custom ceramic ferrule for laser connection

TAHOFs in the dry state were inserted into a custom ceramic ferrule (inner diameter: 650 μm). The assembly was then immersed in PBS for 4 h to reach hydrogel swelling equilibrium. Upon swelling, the fiber diameter slightly exceeded the ferrule inner diameter, forming a tight physical coupling at the fiber-ferrule interface. The ferrule-coupled fiber was subsequently connected to the laser source via an optical patch cable. Unless otherwise stated, all optical characterizations and in vivo experiments used this coupling strategy. For control HOFs (PHEMA core fibers), the same procedure was applied using a 550 μm-inner-diameter ferrule.

### Optical propagation loss and deformation-dependent transmission measurement of TAHOFs

The optical propagation loss (in dB/cm) of fully hydrated TAHOFs in air was measured using the cutback method^17,19^. Fibers were optically coupled to a 470 nm blue laser source (Aurora-300, Newdoon, China) via ferrule-to-ferrule alignment using zirconia sleeves, with transmitted intensity recorded via a calibrated LP10 power meter (Sanwa, Japan). Sequential 1 cm excisions were performed using a precision blade, with output power measurements taken after each resection. Propagation loss calculations were derived from transmission measurements at 1 cm intervals. For biological tissue transmission evaluation, fibers with lengths of 3, 6, or 12 cm were placed in direct contact with porcine skin specimens, with distal output intensity normalized to air measurements under identical conditions.

Real-time strain-responsive transmission was characterized using a 470 nm laser source and inline power meter configuration. During measurements, the fibers were maintained in a high-humidity chamber using a humidifier to minimize water loss. Samples were manually stretched to predesignated strains (0%, 10%, 20%, 30%, 50%, and 60%), and transmitted light intensity was continuously recorded. Cyclic durability assessment involved 10,000 stretching cycles at 30% strain, conducted in aqueous immersion using a horizontal tensile system (CellScale, Canada). Optical transmission stability was continuously monitored throughout mechanical testing.

### Tissue bioadhesion test of TAHOFs

To evaluate the tissue bioadhesion strength of the hydrogel cladding, adhesive films ( ~ 300 μm in thickness) were prepared using the same formulation and fabrication procedure described in the “*Fabrication of the tissue-adhesive hydrogel cladding layer*” section. A standard lap-shear test was performed on adhesive joints formed between the hydrogel adhesive and various porcine tissues, including intestine, fillet, skin, heart, and liver. Briefly, the adhesion tissue joints were assembled by laminating two pieces of porcine tissue with a dry hydrogel film positioned in between, followed by gentle compression ( ~ 1 kPa) for 30 seconds prior to lap shear testing. To prevent elastic deformation of the soft tissues during testing, ~100 μm-thick nylon backing layers were bonded to the opposite side of the tissues using cyanoacrylate glue.

To assess long-term bioadhesion stability under physiological conditions, the hydrogel films were adhered to porcine skin, with both tissues ends fixed in a horizontal tensile testing system (CellScale). The assembled adhesion joints were immersed in PBS buffer and subjected to 10,000 cycles of bending deformation at 30% strain. Following cyclic loading, the remained adhesive strength was evaluated by lap-shear testing.

### Numerical modeling of fiber-tissue biomechanical interactions

A three-dimensional finite element model was developed in ABAQUS (2016) to assess the mechanical conformity and illumination stability of TAHOFs under physiological motion^10^. The simulation framework incorporated four fiber material configurations: silica (Young’s modulus: 50 GPa; Poisson’s ratio: 0.17)^13^, polycarbonate (*E* = 3 GPa, *ν* = 0.37)^29^, non-adhesive hydrogel fibers (HOF), and TAHOF, with identical cylinders shape of 20 mm × 650 μm. Both hydrogel-based fibers were assigned experimentally determined elastic properties (Young’s modulus: 0.485 MPa; Poisson’s ratio: 0.46). The surrounding subcutaneous tissue was represented as a neo-Hookean material mimicking muscle, with a Young’s modulus of 0.12 MPa and a Poisson’s ratio of 0.5^29^, and discretized using 20-node hybrid hexahedral elements (C3D20RH), while fibers employed 8-node reduced integration elements (C3D8R). Physiological micromotion (2 mm lateral displacement) was applied to the tissue basal surface, with proximal fiber fixation simulating surgical anchoring. TAHOFs were assigned a bonded interface with an interfacial shear strength of 10 kPa, while other fibers were coupled via frictional contact (coefficient = 0.3) to simulate non-adhesive engagement^13^.

Spatial targeting fidelity was evaluated through vector summation of triaxial displacements (*ΣΔx, Δy, Δz*) between fiber tip emission points and a predefined tissue-surface reference marker. Concurrently, von Mises stress distributions were analyzed to identify localized concentrations arising from modulus mismatch at fiber-tissue interfaces. This dual assessment framework enabled simultaneous evaluation of optical positioning stability and mechanical compatibility under simulated physiological loading conditions including respiration, cardiac contraction, and musculoskeletal movement.

### Ethics

All animal procedures were approved by the Ethics Committee for Animal Research of the Shenzhen Institutes of Advanced Technology, Chinese Academy of Sciences, and conducted in strict accordance with institutional guidelines (Ethics approval No. SIAT-IACUC-221209-NS-LY-A2232).

### Animals

ChAT-ChR2 mice were generated by crossing ChAT-Cre mice (Jackson Laboratory, stock no. 018957) with Ai27D reporter mice (Jackson Laboratory, stock no. 012567). Male ChAT-Cre mice and ChAT-ChR2 mice (6-10 weeks old) and C57BL/6 J wild-type mice (6-10 weeks old, Charles River Laboratories) were used for experiments. Only male mice were used in this study to improve the stability of the STZ-induced hyperglycemic model and to minimize variability associated with estrous-cycle-related hormonal fluctuations in females, which can influence glucose homeostasis and insulin sensitivity and thereby increase variability in the physiological readouts measured in this study^4,40^. Animals were housed under controlled conditions with a constant temperature of 24 ± 1 °C, relative humidity between 50% and 60%, and a 12-hour light/dark cycle (lights on from 08:00 to 20:00). Food and water were available *ad libitum*.

### Immunohistochemistry

Excised pancreas tissues were fixed in 4% paraformaldehyde (PFA) overnight at 4 °C, embedded in optimal cutting temperature (OCT) compound, and cryosectioned. Sections were washed three times with PBS and stained with DAPI (1:5000, Beyotime) for nuclear labeling. After staining, samples were washed again with PBS and mounted using antifade mounting medium. Fluorescence imaging was performed using an automated slide scanner (VS120, Olympus, Japan).

### Magnetic Resonance Imaging (MRI)

TAHOF and HOF fibers were surgically implanted onto the mouse pancreas. In vivo localization was assessed by MRI on postoperative days 1, 3, and 14. Mice were anesthetized with isoflurane (3% induction, 1.5% maintenance; 1 L/min flow) and positioned prone for imaging. MRI was performed using a 9.4 T preclinical system (uMR 9.4 T, United Imaging Life Science Instrument, Wuhan, China) with an 86 mm volume coil for transmission and a three-channel Rat Brain Surface Coil (RBSC3) for reception. T2-weighted fast spin echo (FSE) sequences were acquired with FOV = 37 × 36 mm², matrix = 296 × 288, repetition time (TR) = 3870 ms, echo time (TE) = 22.5 ms, in-plane spatial resolutio*n*= 0.15 × 1 mm², slice thickness = 1 mm, and number of slices = 13.

### Optogenetic stimulation and electrophysiological recording

Subdiaphragmatic vagal fibers were surgically exposed under aseptic conditions and separated from the esophagus. A cuff electrode (inner diameter: 0.2 mm; edge length: 0.5 mm; inter-electrode spacing: 1 mm) was gently wrapped around the vagal trunk for electrophysiological recording. The TAHOF was positioned adjacent to the vagus nerve and adhered to surrounding tissues to ensure stable light delivery. Blue light stimulation (470 nm, 20 Hz, 5 ms, 20 mW) was applied in ChAT-ChR2 mice. Controls included ChAT-Cre mice (termed as ChAT-ChR2(−)) under blue light and ChAT-ChR2 mice under 589 nm illumination. Local field potentials (LFPs) were recorded using a Plexon multichannel system (1 kHz sampling, 1–500 Hz bandpass). Signal processing and analysis were conducted using alongside NeuroExplorer software (Version 5.437, Plexon, USA).

### Open field test (OFT)

To assess whether fiber implantation and optogenetic stimulation of vagal fibers innervating the pancreas affected locomotor activity, three groups of mice were compared: sham-operated controls, fiber-implanted but unstimulated, and fiber-implanted with optical stimulation. Mice were placed individually in an open field chamber (50 × 50 × 50 cm) for 3 minutes of free exploration. In the stimulated group, 470 nm light pulses (20 mW, 20 Hz, 5 ms pulse duration) were delivered to the pancreatic region. Mouse behavior was recorded via digital video and analyzed using EthoVision XT software (version 16.0.1536, Noldus) to quantify locomotor parameters.

### Diabetic mouse model

An insulin-deficient diabetic mouse model was established by intraperitoneal injections of streptozotocin (STZ; Sigma) freshly dissolved in 0.05 M sodium citrate buffer (pH 4.5) at 60 mg kg^−^^1^ body weight. Mice received intraperitoneal injections every other day, for a total of four administrations. Prior to each injection, mice were fasted for 6 h from food and water. Blood glucose levels were measured via tail vein sampling, and mice with fasting blood glucose exceeding 16.7 mmol/L for three consecutive days were classified as diabetic and used for subsequent experiments.

### In vivo implantation of TAHOFs

ChAT-ChR2 mice were anesthetized under thermoregulated surgical conditions. A 1–1.5 cm midline abdominal incision was made adjacent to the pancreas to expose the pancreatic surface. TAHOF samples (650 μm diameter × 20 mm length) were carefully positioned on the pancreas using gentle manual pressure (approximately 1 kPa for 30 s) to facilitate hydrogen bonding and electrostatic adhesion, followed by hydrogel-mediated stabilization along the subcutaneous trajectory. The proximal ends of the fibers incorporated optical ferrules, which were externalized through the incision and connected to 470 nm laser sources via patch cables. The abdominal musculature and skin were closed in layers using 4-0 PLGA sutures (Jinhuan, Shanghai, China), and ferrules were permanently fixed to the skin surface with light-cured dental cement (LAT SCIENCE, China). When optical stimulation was not being applied, the externalized ceramic ferrule was covered with its matching protective cap, and the ferrule cavity was filled with PBS ( ~ 300–400 μL) to maintain hydration at the hydrogel fiber-ferrule interface. The PBS was replenished every 12 h to reduce dehydration of the hydrogel fiber.

### Optogenetic stimulation for modulating insulin secretion

Optical stimulation (470 nm, 5 ms pulse, 20 Hz) was delivered to the pancreas through the implanted TAHOF using an Aurora-300 laser system (Newdoon, China). Optical output power was calibrated to 20 mW at the fiber tip using an inline power meter (PM16-122, Thorlabs). To evaluate neuromodulation effects on glucose metabolism and insulin secretion, intraperitoneal glucose tolerance tests (GTTs) were performed on urethane-anesthetized ChAT-ChR2 mice (1.5 g/kg *i.p*.) following a 10 h fasting. Immediately after intraperitoneal glucose administration (2 g/kg), optical stimulation was initiated. Blood glucose was monitored by tail vein sampling at 15, 30, 60, and 120 min using a FreeStyle Libre continuous glucose monitoring system (Abbott). Concurrently, blood samples (50–60 μL) were collected from the orbital venous sinus into heparinized capillaries for plasma insulin quantification via ELISA (10-1249-01, Mercodia, Sweden).

### Integrated optogenetic glycemic control in diabetic mice

STZ-induced hyperglycemic ChAT-ChR2 mice (fasting glucose > 16.7 mmol/L for at least 3 days) underwent dual implantation of TAHOFs on the pancreas and subcutaneous continuous glucose monitoring (CGM) sensors (SiBiONICS®: 33.5 × 20 × 5.3 mm, 4 g) on the contralateral abdominal wall. After 24 h of recovery, mice received daily 90 min pulsed optical stimulation sessions (470 nm, 5 ms pulses at 20 Hz) while freely moving, repeated for three consecutive days. At study conclusion, cardiac blood was collected (100–150 μL) for comprehensive hematological analysis, and major organs (heart, liver, spleen, lungs, kidneys) were harvested for histopathological evaluation using hematoxylin and eosin (HE) staining to assess systemic biocompatibility. Control groups included non-stimulated TAHOF-implanted mice and mice implanted with hydrogel optical fibers lacking adhesive cladding (HOF: PHEMA core only), permitting assessment of both optical stimulation efficacy and fiber adhesion importance in glycemic modulation.

### Chronic optogenetic stimulation via implanted TAHOF

STZ-induced diabetic ChAT-ChR2 mice were anesthetized with isoflurane (2% in oxygen for induction and 1.5% in oxygen for maintenance). A 1–1.5 cm abdominal incision was made to expose the pancreas, and a TAHOF sample was implanted onto its surface. The fiber was anchored along the subcutaneous pathway via its adhesive cladding, while the proximal end was coupled to a ceramic ferrule secured dorsally using dental cement. Mice received pulsed stimulation (470 nm, 20 Hz, 5 ms) for 60 min at post-implantation days 1, 3, 7, 10, and 14. Blood glucose was measured every 20 min via tail-vein sampling during 60 min pre-stimulation, stimulation, and post-stimulation phases. Control animals underwent identical procedures without optical stimulation. Postoperatively, mice received meloxicam (2 mg/kg) and cephalexin (15 mg/kg) once daily for 3 days.

### Statistical analysis

The statistical analysis was conducted at least four times (*n* ≥ 4) for each sample, and all the data were processed using Origin 2024 and presented as the means ± standard deviation (SD) or means ± standard error (SE). One-way analysis of variance (One-way ANOVA) was used to determine the significance level between multiple groups. The significance levels were considered as *p < 0.05, **p < 0.01, ***p < 0.001. For statistical analysis between two groups, two-way repeated measures ANOVA followed by Bonferroni post hoc test or the two-sided Student’s t-test was used with a significance threshold of *p < 0.05, **p < 0.01, ***p < 0.001.

### Reporting summary

Further information on research design is available in the Nature Portfolio Reporting Summary linked to this article.

## Supplementary information

Supplementary Information

Description of Additional Supplementary Files

Supplementary Movie 1.

Supplementary Movie 2.

Supplementary Movie 3.

Supplementary Movie 4.

Supplementary Movie 5.

Reporting Summary

Transparent Peer Review file

## Source data

Source data

## Data Availability

All data supporting the findings of this study are available within the article and its supplementary files. Any additional requests for information can be directed to and will be fulfilled by the corresponding authors. Source data are provided with this paper.
