# Supplementary material for: Tissue-adhesive hydrogel optical fiber for peripheral optogenetic neuromodulation
Source: Nat Commun. 2026 Jun 29;17:8101. doi: 10.1038/s41467-026-74831-1 (PMC13458817; doi:10.1038/s41467-026-74831-1)
Supplement: Supplementary file 1 [file 41467_2026_74831_MOESM1_ESM.pdf]

Supplementary Information

Tissue-adhesive Hydrogel Optical Fiber for Peripheral Optogenetic Neuromodulation

Xingmei Chen [1,2†], Lulu Wang [3,4†], Chang Wang[1], Yafei Wang [1], Liangjie Shan [1], Yu Xue [1], Zhongjie Ma[1], Cunjiang Yu [5,6,7,8*], Yi Lu [3,4*], Ji Liu[1*]

Affiliations

[1] Department of Mechanical and Energy Engineering, Southern University of Science and Technology, Shenzhen 518055, China.

[2] Materials Research Laboratory, University of Illinois, Urbana-Champaign, Urbana, IL 61801, USA.

[3] State Key Laboratory of Brain Cognition and Brain-inspired Intelligence Technology, Shenzhen Institutes of Advanced Technology (SIAT), Chinese Academy of Sciences (CAS), Shenzhen, 518055, China.

[4] Shenzhen-Hong Kong Institute of Brain Science, Shenzhen Institutes of Advanced Technology (SIAT), Chinese Academy of Sciences (CAS), Shenzhen, 518055, China.

[5] Department of Electrical and Computer Engineering, University of Illinois, Urbana-Champaign, Urbana, IL 61801, USA.

[6] Department of Materials Science and Engineering, University of Illinois, Urbana-Champaign, Urbana, IL 61801, USA.

[7] Department of Mechanical Science and Engineering, University of Illinois, Urbana-Champaign, Urbana, IL 61801, USA.

[8] Department of Bioengineering, University of Illinois, Urbana-Champaign, Urbana, IL 61801, USA.

[*] **Corresponding Authors**

liuj9@sustech.edu.cn (J. Liu)

luyi@siat.ac.cn (Y. Lu)

cunjiang@illinois.edu (C. Yu)

[†] These authors contributed equally.

Contents

Supplementary Methods
Supplementary Figs. 1-34
Supplementary Table
Supplementary Movies 1-5
Supplementary References

Supplementary Text

Materials

Hydroxyethyl methacrylate (HEMA, TCI), 2-hydroxy-2-methylpropiophenone (2-HMP, TCI), and poly(ethylene glycol) diacrylate (PEG(700)-DA, MW = 700 Da, Sigma-Aldrich) were used to synthesize the hydrogel core fiber. To fabricate the tissue-adhesive hydrogel cladding, polyvinyl alcohol (PVA, MW 89-98 kDa, > 99% hydrolyzed, Sigma-Aldrich), acrylic acid (AA, TCI), α-ketoglutarate (α-KA, Adamas), glutaraldehyde (GA, Adamas), and PEG(700)-DA were used to form a chemically crosslinked double-network hydrogel. Blood glucose levels were monitored using either a handheld glucose meter (FreeStyle Libre, Abbott) or a dynamic continuous glucose monitoring (CGM) system (SiBiONICS®) via tail vein sampling or subcutaneous implantation in ChAT-ChR2 mice, respectively. PBS buffer purchased from Gibco (USA). Unless otherwise stated, all reagents used for in vivo experiments were obtained from Jackson ImmunoResearch (USA) or Servicebio (Wuhan, China).

Sample fabrication

Preparation of the optical hydrogel fiber: Fabrication of PHEMA hydrogel core fibers: The hydrogel core precursor solution was formulated by combining 80 *wt*% hydroxyethyl methacrylate (HEMA), 1 *wt*% 2-hydroxy-2-methylpropiophenone (2-HMP) photoinitiator, and 2 *wt*% poly(ethylene glycol) diacrylate (PEG(700)-DA) crosslinker in aqueous medium. This mixture was syringe-injected into silicone tube molds (ID: 550 μm; OD: 1200 μm) and photopolymerized under UV irradiation (365 nm, 20 $mW\cdot cm^{-2}$) for 45 min. Upon curing, the mold underwent dichloromethane (DCM) immersion (20 min) to facilitate silicone swelling and subsequent core fiber demolding. Residual monomers were removed through sequential solvent treatment: initial ethanol soak (50% v/v, 1 h) followed by distilled water immersion (24 h) for solvent exchange.

Fabrication of the tissue-adhesive hydrogel cladding layer: A dual-network cladding precursor solution was consisted of 10 *wt*% acrylic acid (AA), 0.5 wt% α-ketoglutarate (α-KA), PEG(700)-DA crosslinker, 10 *wt*% poly(vinyl alcohol) (PVA), and 0.03 *wt*% glutaraldehyde (GA). Additionally, 0.03 M hydrochloric acid (HCl) was added to catalyze the chemical crosslinking of PVA. After vigorous stirring and degassing (2 min), the solution was infused into Tygon® tubing (ID: 760 μm; OD: 2290 μm) housing pre-dried PHEMA core fibers. UV exposure (365 nm, 60 mW cm^{-2}, 15 min) initiated poly(acrylic acid) (PAA)

network formation, followed by 1 h room-temperature incubation to complete PVA chemical crosslinking. Core-cladding fibers were released through DCM-assisted demolding (20 min), air-dried. The resulting tissue-adhesive hydrogel optical fiber (TAHOF) were then purified by soaking in distilled water for 3 days to remove residual monomers and uncrosslinked polymers.

Instrumentation and measurements

Microstructure characterization

Optical images and surface microtopography of the TAHOF were obtained using a stereo microscope (SZX16, Olympus, Japan). To visualize the internal microstructures of the hydrogel cladding, a trace amount of fluorescein isothiocyanate-labeled acrylic acid (0.03 wt%, FITC-AA) was added to the monomer precursor solution prior to UV-induced crosslinking of the PAA network. The fluorescently labeled hydrogel fibers were subsequently imaged using a confocal laser scanning microscope (CLSM, TCS SP8, Leica). Unless otherwise specified, all photographs were acquired using a digital camera (D7500, Nikon, Japan).

Optical properties of TAHOFs

TAHOFs are consisted of a poly(2-hydroxyethyl methacrylate) (PHEMA) hydrogel core and a tissue-adhesive cladding layer. The optical transmission spectra of both the PHEMA core and the cladding hydrogel were measured using a UV/VIS/NIR spectrophotometer (Lambda 950, PerkinElmer, USA). The typical thicknesses of the PHEMA core and cladding layer were approximately 550 μm and 100 μm, respectively. The refractive indices (RI) of each hydrogel (2 mm thickness) were determined using an Abbe refractometer (Lichen Bangxi Instrument Technology Co., Ltd., China).

Optical propagation loss and deformation-dependent transmission measurement of TAHOFs

The optical propagation loss (in dB cm^{-1}) of fully hydrated TAHOFs in air was measured using the cutback method [1]. Fibers were optically coupled to a 470 nm blue laser source (Aurora-300, Newdoon, China) via ferrule-to-ferrule alignment using zirconia sleeves, with transmitted intensity recorded via a calibrated LP10 power meter (Sanwa, Japan). Sequential 1 cm excisions were performed using a precision blade, with output power measurements taken after each resection. Propagation loss calculations were derived from transmission measurements at 1 cm intervals. For biological tissue transmission evaluation, fibers with lengths of 3, 6, or 12 cm were placed in direct contact with porcine skin specimens, with distal output

intensity normalized to air measurements under identical conditions.

Real-time strain-responsive transmission was characterized using a 473 nm laser source and inline power meter configuration. During measurements, the fibers were maintained in a high-humidity chamber using a humidifier to minimize water loss. Samples were manually stretched to predesignated strains (0%, 10%, 20%, 30%, 50%, and 60%), and transmitted light intensity was continuously recorded. Cyclic durability assessment involved 10,000 stretching cycles at 30% strain, conducted in aqueous immersion using a horizontal tensile system (CellScale, Canada). Optical transmission stability was continuously monitored throughout mechanical testing. All quantitative analyses were performed with five independent fiber samples.

FDTD simulation of light propagation in TAHOF-tissue systems

Finite-difference time-domain (FDTD) simulations were performed in Ansys Lumerical FDTD (2020 r2) to model the propagation of 470 nm Gaussian light through TAHOFs embedded in biological tissue. The computational domain incorporated scaled fiber dimensions (1:100 reduction from experimental PHEMA core radius of 275 μm and cladding thickness of 50 μm) with a 30 nm mesh resolution, balancing simulation accuracy and computational efficiency. Material optical properties were established as follows: RI of 1.429 (PHEMA core), 1.342 (adhesive cladding), and 1.500 (surrounding tissues). A muscle-equivalent coefficient of 890 m^{-1} was selected for the tissue absorption characteristics modeled [2]. Light intensity profiles and transmission along the propagation axis were numerically solved and analyzed through MATLAB 2018b, enabling quantitative evaluation of guided light transmission in simulated tissue environments.

Tissue bioadhesion test of TAHOFs

To evaluate the tissue bioadhesion strength of the hydrogel cladding, adhesive films (~ 300 μm in thickness) were prepared using the same formulation and fabrication procedure described in the "*Fabrication of the tissue-adhesive hydrogel cladding layer*" section. A standard lap-shear test was performed on adhesive joints formed between the hydrogel adhesive and various porcine tissues, including intestine, fillet, skin, heart, and liver. Briefly, the adhesion tissue joints were assembled by laminating two pieces of porcine tissue with a dry hydrogel film positioned in between, followed by gentle compression (~1 kPa) for 30 seconds prior to lap shear testing. To prevent elastic deformation of the soft tissues during

testing, ~ 100 μm-thick nylon backing layers were bonded to the opposite side of the tissues using cyanoacrylate glue.

To assess long-term bioadhesion stability under physiological conditions, the hydrogel films were adhered to porcine skin, with both tissues ends fixed in a horizontal tensile testing system (CellScale). The assembled adhesion joints were immersed in PBS buffer and subjected to 10,000 cycles of bending deformation at 30% strain. Following cyclic loading, the remained adhesive strength was evaluated by lap-shear testing.

Mechanical tests of TAHOFs

The stress-strain behavior of TAHOFs samples was measured using a horizontal tensile testing system equipped with a 4.4 N load cell (CellScale, Canada). Hydrogel fiber samples were immersed in PBS during testing and stretched at a constant rate of 100 mm min^{-1}. Force-displacement curves were recorded and converted into stress-strain curves. Nominal stress was calculated by dividing the tensile force by the initial cross-sectional area of the fiber (diameter~ 650 μm), and strain was defined as the displacement normalized to the initial gauge length. From the resulting stress–strain curves, several mechanical parameters were extracted including maximum strain, tensile strength and Young's modulus. In addition, cyclic tensile tests were performed using the same instrument to evaluate mechanical durability. TAHOFs samples were stretched under a fixed strain amplitude of 30% for 10,000 loading cycles.

The interfacial toughness between the PHEMA hydrogel core and the tissue-adhesive cladding was assessed using the same formulations and fabrication procedures described in the "*Preparation of the TAHOFs*" section. A 90° peeling test was conducted to quantify the interfacial strength of the bonded hydrogel layers. Briefly, the precursor solution of the tissue-adhesive cladding was cast directly onto the surface of the PHEMA hydrogel fiber and photopolymerized under UV irradiation (365 nm) for 15 min to form a covalently crosslinked hydrogel network. The resulting hydrogel composite was air-dried overnight, after which nylon backing layers were attached to the opposite side using traceable amount of cyanoacrylate glue, in order to provide structural support and minimize deformation during testing. Before mechanical evaluation, the hydrogel materials were immersed in PBS for 4 h to reach swelling equilibrium.

Numerical modeling of fiber-tissue biomechanical interactions

A three-dimensional finite element model was developed in ABAQUS (2016) to assess the mechanical conformity and illumination stability of TAHOFs under physiological motion [3] The simulation framework incorporated four fiber material configurations: silica (Young's modulus: 50 GPa; Poisson's ratio: 0.17)[4], polycarbonate (E = 3 GPa, ν = 0.37) [5] , non-adhesive hydrogel fibers (HOF), and TAHOF, with identical cylinders shape of 20 mm × 650 μm. Both hydrogel-based fibers were assigned experimentally determined elastic properties (Young's modulus: 0.485 MPa; Poisson's ratio: 0.46). The surrounding subcutaneous tissue was represented as a neo-Hookean material mimicking muscle, with a Young's modulus of 0.12 MPa and a Poisson's ratio of 0.5[5], and discretized using 20-node hybrid hexahedral elements (C3D20RH), while fibers employed 8-node reduced integration elements (C3D8R). Physiological micromotion (2 mm lateral displacement) was applied to the tissue basal surface, with proximal fiber fixation simulating surgical anchoring. TAHOFs were assigned a bonded interface with an interfacial shear strength of 10 kPa, while other fibers were coupled via frictional contact (coefficient = 0.3) to simulate non-adhesive engagement[4].

Spatial targeting fidelity was evaluated through vector summation of triaxial displacements ($\Sigma\Delta x$, Δy, Δz) between fiber tip emission points and a predefined tissue-surface reference marker. Concurrently, von Mises stress distributions were analyzed to identify localized concentrations arising from modulus mismatch at fiber-tissue interfaces. This dual assessment framework enabled simultaneous evaluation of optical positioning stability and mechanical compatibility under simulated physiological loading conditions including respiration, cardiac contraction, and musculoskeletal movement.

Ethics

All animal procedures were approved by the Ethics Committee for Animal Research of the Shenzhen Institutes of Advanced Technology, Chinese Academy of Sciences, and conducted in strict accordance with institutional guidelines (Ethics approval No. SIAT-IACUC-221209-NS-LY-A2232).

Animals

ChAT-ChR2 mice were generated by crossing ChAT-Cre mice (Jackson Laboratory, stock no. 018957) with Ai27D reporter mice (Jackson Laboratory, stock no. 012567). Male ChAT-Cre mice and ChAT-ChR2 mice (6-10 weeks old) and C57BL/6J wild-type mice (6-10 weeks old, Charles River Laboratories) were used for experiments. Only male mice were used in this study to improve the stability of the STZ-

induced hyperglycemic model and to minimize variability associated with estrous-cycle-related hormonal fluctuations in females, which can influence glucose homeostasis and insulin sensitivity and thereby increase variability in the physiological readouts measured in this study[6,7]. Animals were housed under controlled conditions with a constant temperature of 24 ± 1 °C, relative humidity between 50% and 60%, and a 12-hour light/dark cycle (lights on from 08:00 to 20:00). Food and water were available ad libitum.

Optogenetic stimulation and electrophysiological recording

Subdiaphragmatic vagal fibers were surgically exposed under aseptic conditions and separated from the esophagus. A cuff electrode (inner diameter: 0.2 mm; edge length: 0.5 mm; inter-electrode spacing: 1 mm) was gently wrapped around the vagal trunk for electrophysiological recording. The TAHOF was positioned adjacent to the vagus nerve and adhered to surrounding tissues to ensure stable light delivery. Blue light stimulation (470 nm, 20 Hz, 5 ms, 20 mW) was applied in ChAT-ChR2 mice. Controls included ChAT-Cre mice (termed as ChAT-ChR2(-)) under blue light and ChAT-ChR2 mice under 589 nm illumination. Local field potentials (LFPs) were recorded using a Plexon multichannel system (1 kHz sampling, 1-500 Hz bandpass). Signal processing and analysis were conducted using NeuroExplorer software (Version 5.437, Plexon, USA).

Open field test (OFT)

To assess whether fiber implantation and optogenetic stimulation of vagal fibers innervating the pancreas affected locomotor activity, three groups of mice were compared: sham-operated controls, fiber-implanted but unstimulated, and fiber-implanted with optical stimulation. Mice were placed individually in an open field chamber (50 × 50 × 50 cm) for 3 minutes of free exploration. In the stimulated group, 470 nm light pulses (20 mW, 20 Hz, 5 ms pulse duration) were delivered to the pancreatic region. Mouse behavior was recorded via digital video and analyzed using EthoVision XT software (version 16.0.1536, Noldus) to quantify locomotor parameters.

***In vivo* biocompatibility of TAHOFs**

TAHOFs samples (diameter: 650 μm; length: 1 cm) were sterilized by UV irradiation for 3 hours prior to subcutaneous implantation beneath the mediodorsal skin of C57BL/6J mice. At designated time points (7- and 14-days post-implantation), subcutaneous tissues in direct contact with the hydrogel fibers were harvested, fixed in 4% paraformaldehyde, and embedded in paraffin for histological and

immunofluorescence analyses. In addition, 100-200 μL of blood was collected from each animal via cardiac puncture for complete blood count (CBC) analysis. Mice were euthanized by carbon dioxide (CO_2) inhalation following sample collection.

Histology and immunofluorescence

Fixed tissue samples were sequentially dehydrated in gradient ethanol (75%-90%-95%-100%)[8], and processed for paraffin embedding. Sections were cut at 4 μm thickness using a rotary microtome (RM2016, Leica, Germany) and subjected to hematoxylin and eosin (HE) staining and Masson's trichrome staining to assess general tissue morphology and inflammatory cell infiltration.

For immunofluorescence analysis, paraffin-embedded sections (4 μm) were deparaffinized, rehydrated, and subjected to antigen retrieval using the steam method. After cooling, the sections were washed three times in PBS containing 0.1% Tween 20 (PBS-Tween) and incubated with rabbit anti-CD68 primary antibody (1:200, GB153109-100, Servicebio) at 4 °C for 4 hours. Sections were then washed again in PBS-Tween and incubated with Alexa Fluor 488-conjugated goat anti-rabbit secondary antibody (1:1000, 111-545-144, Jackson ImmunoResearch) at 37 °C for 1 hour. Nuclear counterstaining was performed with DAPI in PBS for 10 minutes. Immunofluorescence images were acquired using a confocal laser scanning microscope (CLSM; SP8, Leica). For quantitative analysis, fluorescence-positive area ratios were calculated using ImageJ (1.52p) from more than 100 randomly selected regions of interest per group.

Diabetic mouse model

An insulin-deficient diabetic mouse model was established by intraperitoneal injections of streptozotocin (STZ; Sigma) freshly dissolved in 0.05 M sodium citrate buffer (pH 4.5) at 60 mg/kg body weight. Male mice received intraperitoneal injections every other day, for a total of four administrations. Prior to each injection, mice were fasted for 6 hours from food and water. Blood glucose levels were measured via tail vein sampling, and mice with fasting blood glucose exceeding 16.7 mmol/L for three consecutive days were classified as diabetic and used for subsequent experiments.

In vivo implantation of TAHOFs

ChAT-ChR2 mice were anesthetized under thermoregulated surgical conditions. A 1-1.5 cm midline abdominal incision was made adjacent to the pancreas to expose the pancreatic surface. TAHOF samples (650 μm diameter × 20 mm length) were carefully positioned on the pancreas using gentle manual pressure (approximately 1 kPa for 30 seconds) to facilitate hydrogen bonding and electrostatic adhesion, followed

by hydrogel-mediated stabilization along the subcutaneous trajectory. The proximal ends of the fibers incorporated optical ferrules, which were externalized through the incision and connected to 470 nm laser sources via patch cables. The abdominal musculature and skin were closed in layers using 4-0 PLGA sutures (Jinhuan, Shanghai, China), and ferrules were permanently fixed to the skin surface with light-cured dental cement (LAT SCIENCE, China). When optical stimulation was not being applied, the externalized ceramic ferrule was covered with its matching protective cap, and the ferrule cavity was filled with PBS (~300-400 μL) to maintain hydration at the hydrogel fiber-ferrule interface. The PBS was replenished every 12 h to reduce dehydration of the hydrogel fiber.

Optogenetic stimulation for modulating insulin secretion

Optical stimulation (470 nm, 5 ms pulse width, 20 Hz frequency) was delivered to the pancreas through the implanted TAHOF using an Aurora-300 laser system (Newdoon, China). Optical output power was calibrated to 20 mW at the fiber tip using an inline power meter (PM16-122, Thorlabs). To evaluate neuromodulation effects on glucose metabolism and insulin secretion, intraperitoneal glucose tolerance tests (GTTs) were performed on urethane-anesthetized ChAT-ChR2 mice (1.5 g/kg i.p.) following a 10-hour fasting. Immediately after intraperitoneal glucose administration (2 g/kg), optical stimulation was initiated. Blood glucose was monitored by tail vein sampling at 15, 30, 60, and 120 minutes using a Free Style Libre continuous glucose monitoring system (Abbott). Concurrently, blood samples (50-60 μL) were collected from the orbital venous sinus into heparinized capillaries for plasma insulin quantification via ELISA (10-1249-01, Mercodia, Sweden).

Integrated optogenetic glycemic control in diabetic mice

STZ-induced hyperglycemic ChAT-ChR2 mice (fasting glucose > 16.7 mmol L^{-1} for at least 3 days) underwent dual implantation of TAHOFs on the pancreas and subcutaneous continuous glucose monitoring (CGM) sensors (SiBiONICS®: 33.5 × 20 × 5.3 mm, 4 g) on the contralateral abdominal wall. After 24 hours of recovery, mice received daily 90-minute pulsed optical stimulation sessions (470 nm, 5 ms pulses at 20 Hz) while freely moving, repeated for three consecutive days. At study conclusion, cardiac blood was collected (100-150 μL) for comprehensive hematological analysis, and major organs (heart, liver, spleen, lungs, kidneys) were harvested for histopathological evaluation using hematoxylin and eosin (HE) staining to assess systemic biocompatibility. Control groups included non-stimulated TAHOF-implanted mice and mice implanted with hydrogel optical fibers lacking adhesive cladding (HOF:

PHEMA core only), permitting assessment of both optical stimulation efficacy and fiber adhesion importance in glycemic modulation.

Chronic optogenetic stimulation via implanted TAHOF.

STZ-induced diabetic ChAT-ChR2 mice were anesthetized with isoflurane (2% in oxygen for induction and 1.5% in oxygen for maintenance). A 1-1.5 cm abdominal incision was made to expose the pancreas, and a TAHOF sample was implanted onto its surface. The fiber was anchored along the subcutaneous pathway via its adhesive cladding, while the proximal end was coupled to a ceramic ferrule secured dorsally using dental cement. Mice received pulsed stimulation (470 nm, 20 Hz, 5 ms) for 60 min at post-implantation days 1, 3, 7, 10, and 14. Blood glucose was measured every 20 min via tail-vein sampling during 60 min pre-stimulation, stimulation, and post-stimulation phases. Control animals underwent identical procedures without optical stimulation. Postoperatively, mice received meloxicam (2 mg/kg) and cephalexin (15 mg/kg) once daily for 3 days.

Gastric optogenetic modulation via TAHOF

To evaluate the feasibility of using TAHOF for optogenetic modulation in another mechanically dynamic peripheral organ, gastric optical stimulation and simultaneous gastric slow-wave recording were performed in ChAT-ChR2 mice. In brief, mice were anesthetized with isoflurane (2% in oxygen for induction and 1.5% in oxygen for maintenance) throughout the procedure. A midline abdominal incision was made to exteriorize the stomach, and the TAHOF was adhered to the lesser curvature of the stomach. For gastric slow-wave recording, one pair of Pt wires (diameter, 58 μm) inserted into the serosal surface of the proximal stomach, approximately 0.3-0.5 cm away from the TAHOF implantation site. The electrodes were secured using a 6-0 purse-string suture. The stomach was then returned to the abdominal cavity, and optical stimulation was applied through the implanted TAHOF (470 nm, 5 ms pulses, 20 Hz) for 3 min. Gastric slow-wave signals were recorded using a Plexon multi-channel neural acquisition system (OmniPlex Release 1.20, Plexon, USA), sampled at 1 kHz, and bandpass filtered 0.08-0.13 Hz. Signal processing and analysis were performed using NeuroExplorer software (Version 5.437, Plexon, USA).

Statistical analysis

The statistical analysis was conducted at least four times ($n \geqslant 4$) for each sample and all the data were

processed using Origin 2024 and presented as the means ± standard deviation (SD) or means ± standard error (SE). One-way analysis of variance (One-way ANOVA) was used to determine the significance level between multiple groups. The significance levels were considered as $^{*}p < 0.05$, $^{**}p < 0.01$, $^{***}p < 0.001$. For statistical analysis between two groups, two-way repeated measures ANOVA followed by Bonferroni post hoc test or the two-sided Student's t-test was used with a significance threshold of $^{*}p < 0.05$, $^{**}p < 0.01$, $^{***}p < 0.001$.

Supplementary Figure

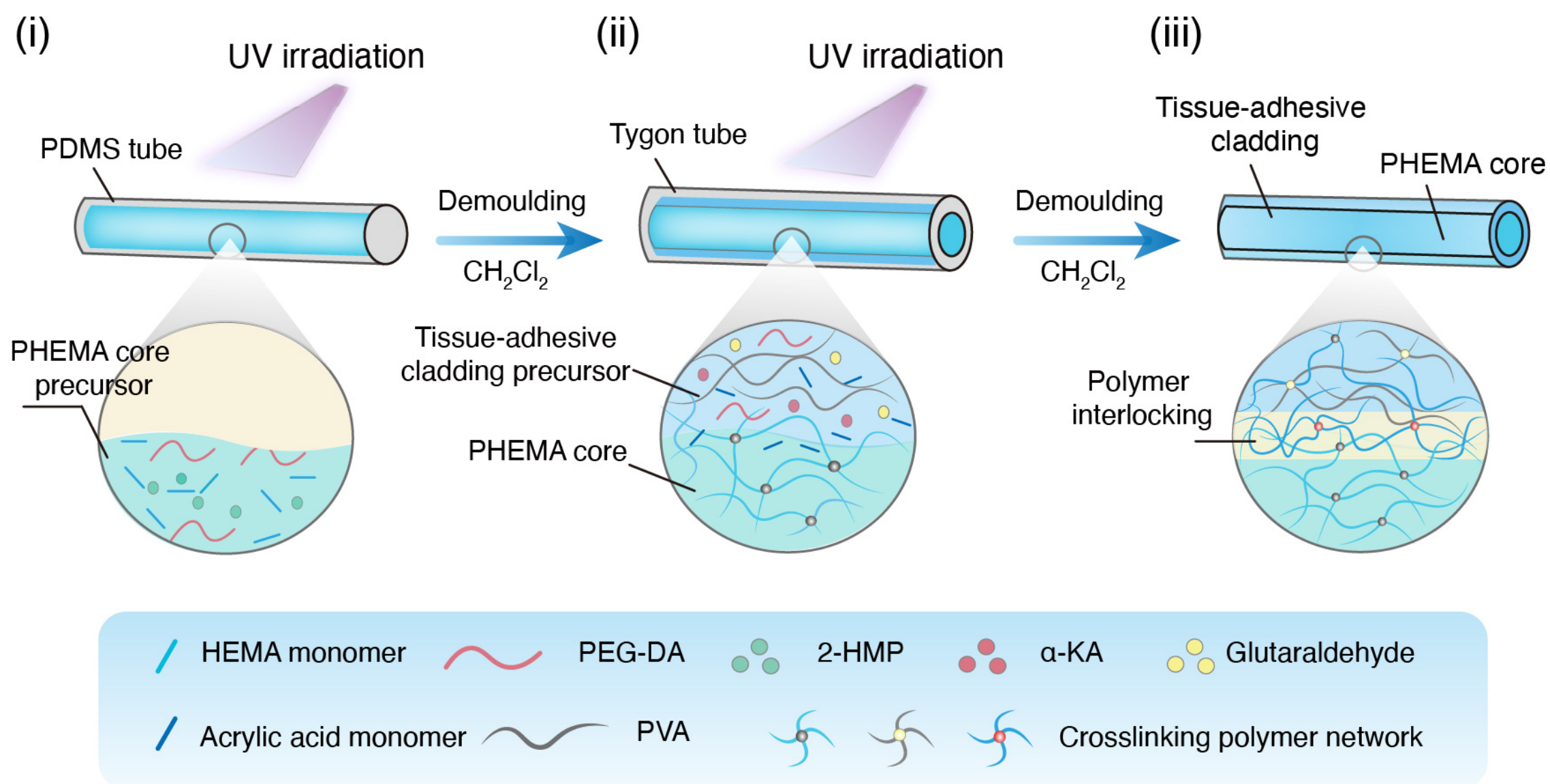

Supplementary Fig. 1 Schematic illustration of the fabrication process for tissue-adhesive hydrogel optical fibers (TAHOFs). (i) A hydrogel core fiber was formed by photopolymerizing a HEMA monomer solution containing PEG(700)-DA as a chemical crosslinker inside a PDMS tube mold. The resulting fiber was demolded in CH_2Cl_2 to yield a flexible and optically transparent core. (ii) For cladding formation, the hydrogel core was inserted into a second mold made of Tygon tubing, pre-filled with a dual-network precursor solution comprising UV-crosslinkable acrylic acid (PAA) and chemically crosslinking poly(vinyl alcohol) (PVA). Upon UV exposure, a conformal adhesion shell formed tightly around the core. (iii) The resulting core–cladding hydrogel fiber was demolded in CH_2Cl_2, exhibiting a robust interfacial structure facilitated by polymer interpenetration and interlocking across the core-cladding boundary [9,10].

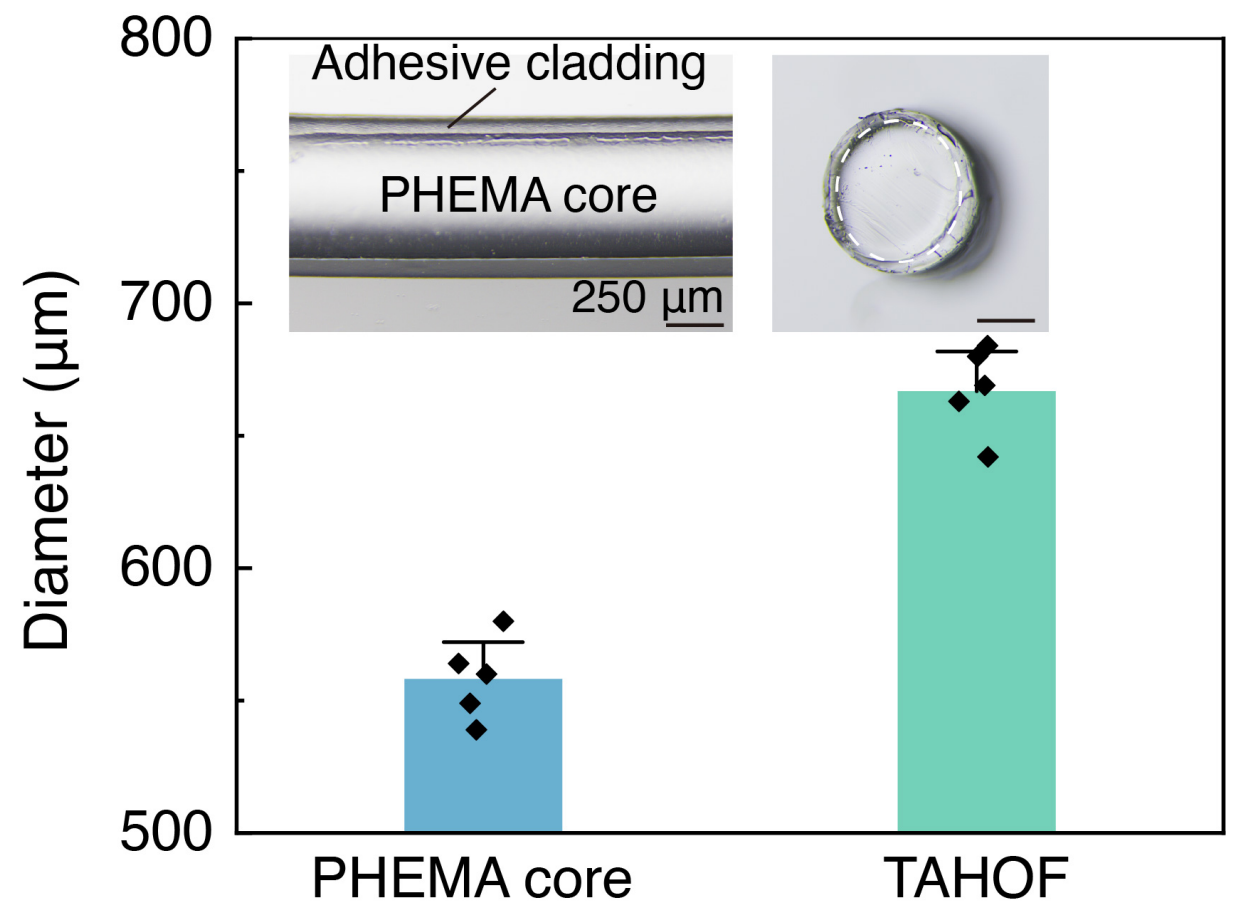

Supplementary Fig. 2 Structural Characterization of TAHOFs samples. The fabricated TAHOFs demonstrate coaxial architecture comprising a poly(HEMA) core (550 µm diameter) surrounded by a uniform tissue-adhesive hydrogel cladding layer (50 µm thickness). Optical microscopy inset highlights the longitudinal profile and cross-sectional interface demarcation between core and cladding components (scale bar: 250 µm). Dimensional measurements represent mean values ± standard deviation from five independent fabrication batches.

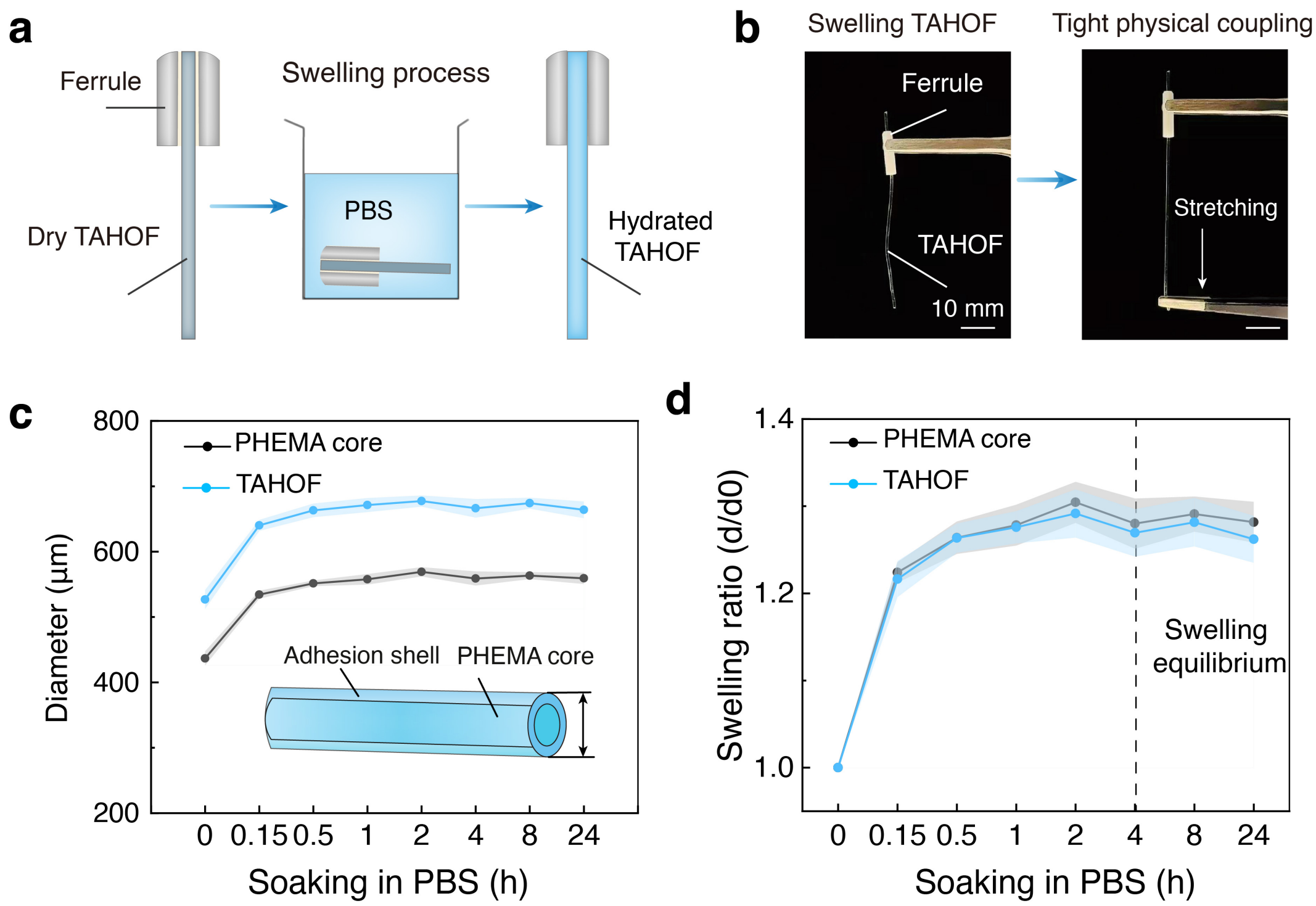

Supplementary Fig. 3 Swelling-assisted coupling of the TAHOF with a ceramic ferrule for optical connection. a Schematic illustration of the TAHOF-ceramic ferrule junction formed by swelling-assisted fitting. **b** Photograph of the tightly coupled TAHOF-ceramic ferrule interface after the hydrogel fiber reached swelling equilibrium. Scale bar, 10 mm. **c-d** Changes in the diameter (**b**) and swelling ratio (**c**) of the TAHOF and PHEMA core during immersion in PBS for 24 h. The data at 0 h correspond to the dry state. Data in (**c**) and (**d**) are presented as mean ± SE (n = 5 independent samples).

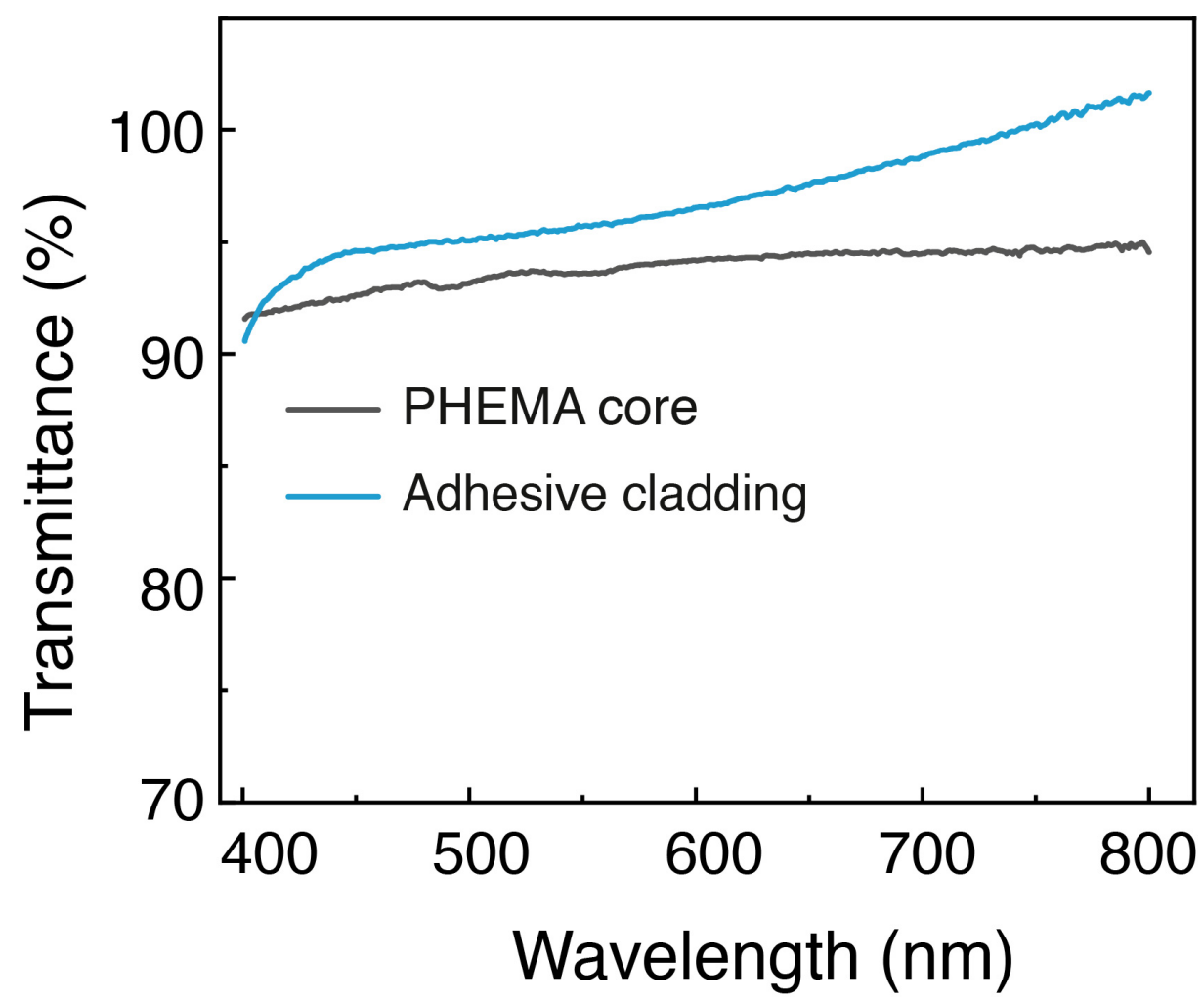

Supplementary Fig. 4 Optical properties of hydrogel fiber components. The hydrogel core and adhesive cladding exhibited high optical transmittance across the visible spectrum (400-780 nm), with both materials maintaining over 90% transparency throughout the entire wavelength range.

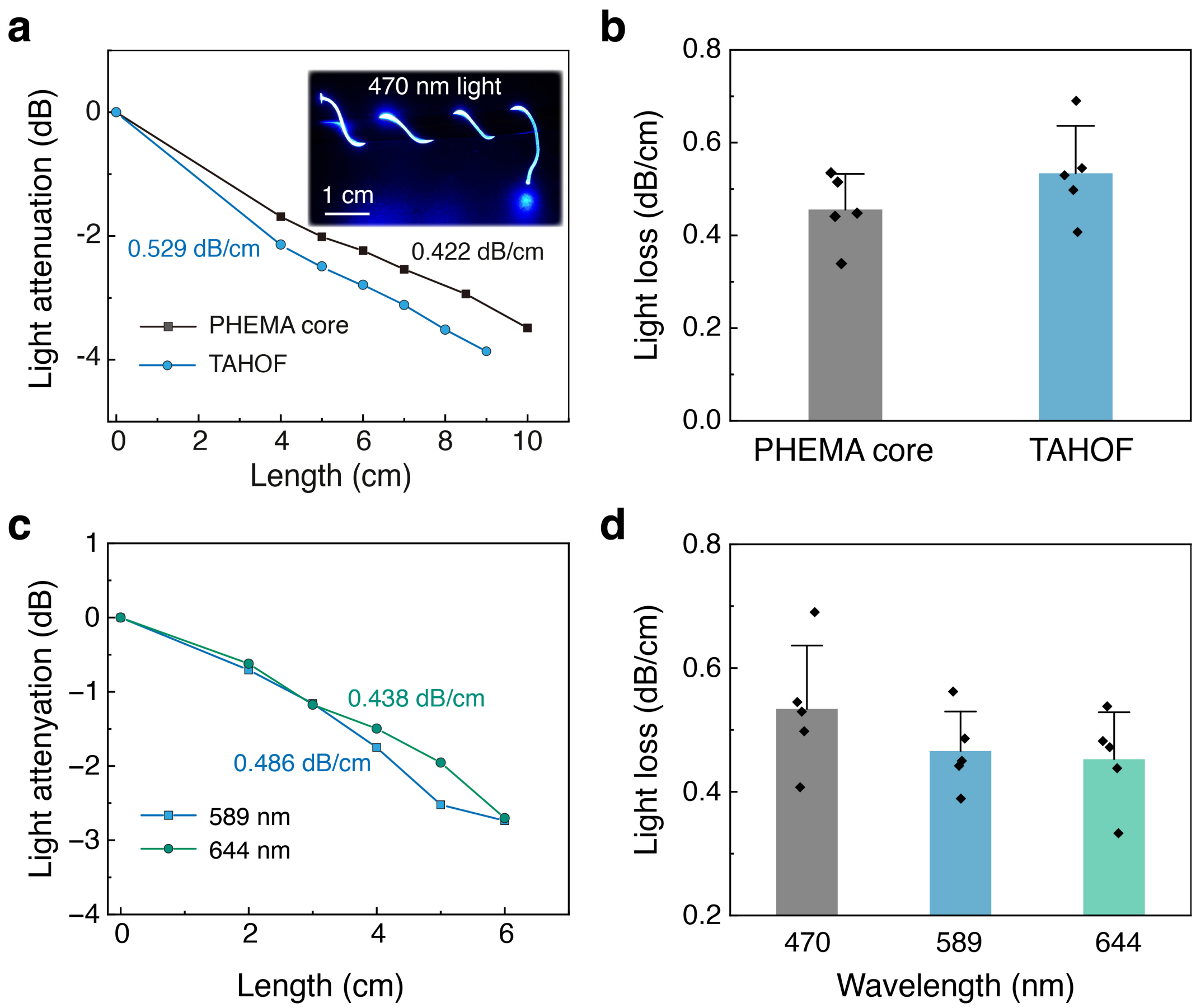

Supplementary Fig. 5 Light propagation in TAHOF. a Light propagation in TAHOF. a Representative propagation loss of TAHOF and PHEMA core fibers at 470 nm, measured by the cutback method. Inset, maintained light guiding of TAHOF during mechanical deformation (rod-wrapping configuration). Scale bar, 1 cm. **b** Quantitative summary of the propagation loss of TAHOF and HOF measured by the cutback method. **c** Representative propagation loss of TAHOF hydrogel fibers at 589 nm and 644 nm, measured by the cutback method. **d** Quantitative summary of the propagation loss of TAHOF at λ = 470, 589, and 644 nm**.** Data are presented as mean ± SD, n = 5 independent samples.

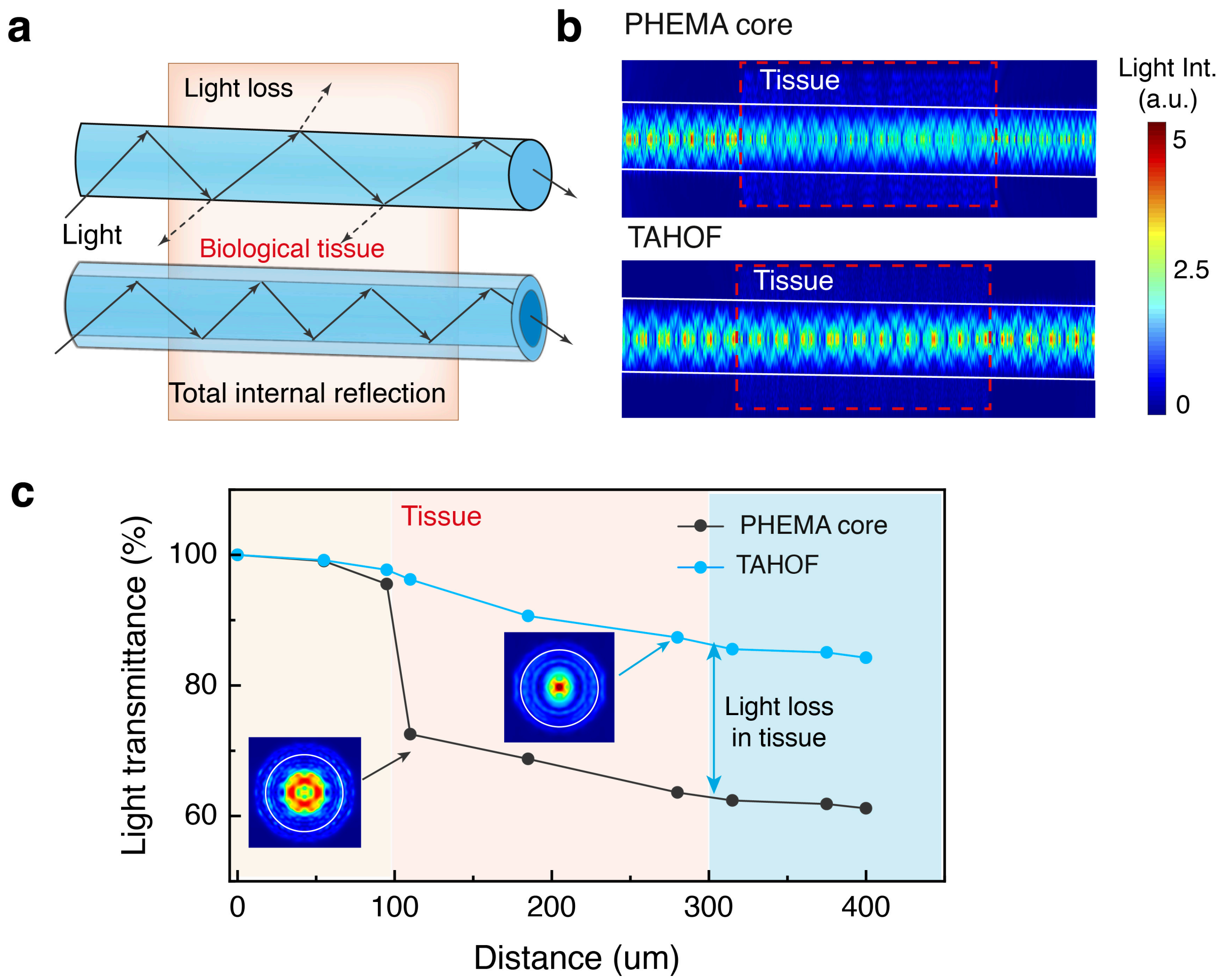

Supplementary Fig. 6 Optical transmission mechanisms in TAHOF implanted in biological tissue. **a** Comparative schematic demonstrates total internal reflection (TIR) maintenance in cladded TAHOFs (n_{core} = 1.429; $n_{cladding}$ = 1.342) versus significant light leakage in core-only fibers when interfaced with higher refractive index biological tissue (n_{tissue} = 1.500). **b** FDTD simulations of 470 nm propagation reveal enhanced waveguide confinement in scaled structural models (TAHOF: 5.5/1 μm core/cladding vs. 6.5 μm core-only configuration) under biologically embedded conditions. **c** Axial light distribution analysis along 400 μm fiber segments quantifies transmission preservation (100-300 μm tissue contact zone) through cross-sectional intensity profiles (insets), validating adhesive cladding's critical role in maintaining optical path integrity.

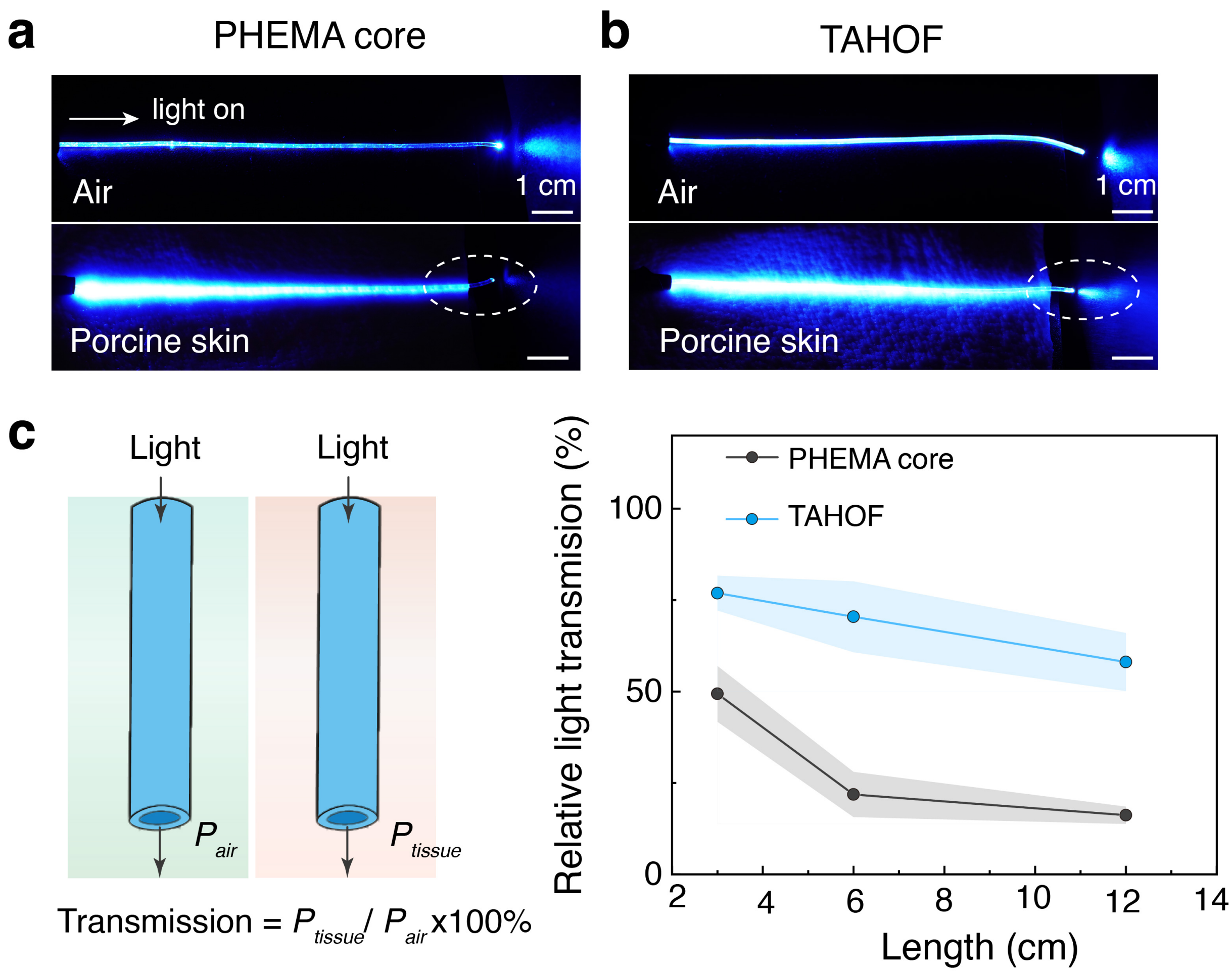

Supplementary Fig. 7 Light propagation performance of TAHOFs on the porcine tissue. a-b Representative images of light transmission through a PHEMA hydrogel core (**a**) and TAHOFs (**b**, with adhesive cladding) in air upon adhesion onto porcine tissues. Scale bar: 1 cm. **c** Normalized light intensity as a function of transmission distance for two fiber upon adhesion onto porcine tissues. The schematic on the left illustrates the calculation method for normalized light intensity. Data are presented as mean ± SD, n = 6 independent samples.

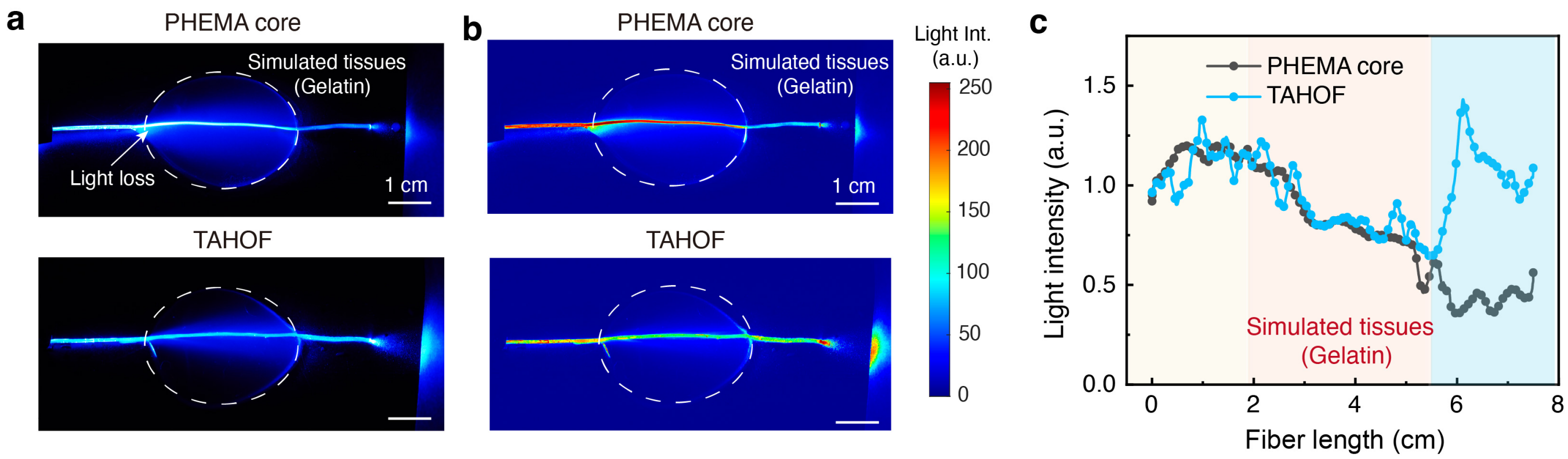

Supplementary Fig. 8 Light delivery performance of TAHOFs in tissue-mimicking environment. a-b Comparative visualization of 470 nm light transmission through PHEMA core (unclad) versus TAHOFs embedded in 15 *wt*% gelatin matrices reveals enhanced waveguide confinement in cladded structures, with corresponding axial intensity profiles quantifying interfacial light leakage reduction. The transparent biological tissue analog enables direct observation of guided propagation characteristics (scale bar: 1 cm). **c** Quantitative analysis derived from normalized intensity profiles demonstrates 38% relative intensity preservation in adhesive-clad TAHOFs compared to core-only configurations when implanted in gelatin hydrogel substrates, validating the cladding layer's critical role in maintaining optical path integrity under simulated physiological conditions.

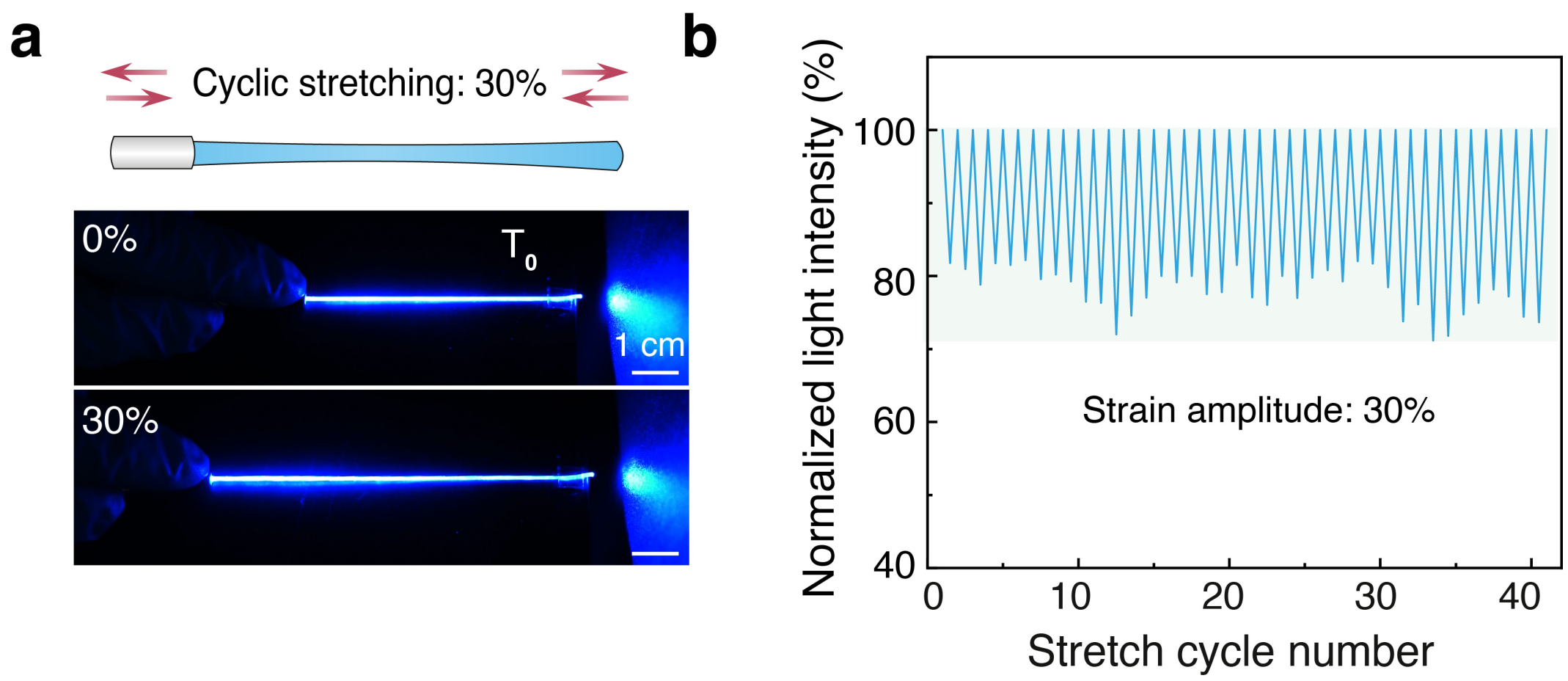

Supplementary Fig. 9 Light propagation in TAHOF under repeated tensile strain. a Comparative visualization demonstrates maintained 470 nm light transmission through TAHOFs under fixed-end configuration, both at rest and during 30% uniaxial tensile strain (scale bar: 1 cm), revealing preserved waveguide integrity despite mechanical deformation. Scale bar: 1 cm. **b** Quantitative tracking of normalized intensity retention over 40 strain cycles at a tensile strain of 30%.

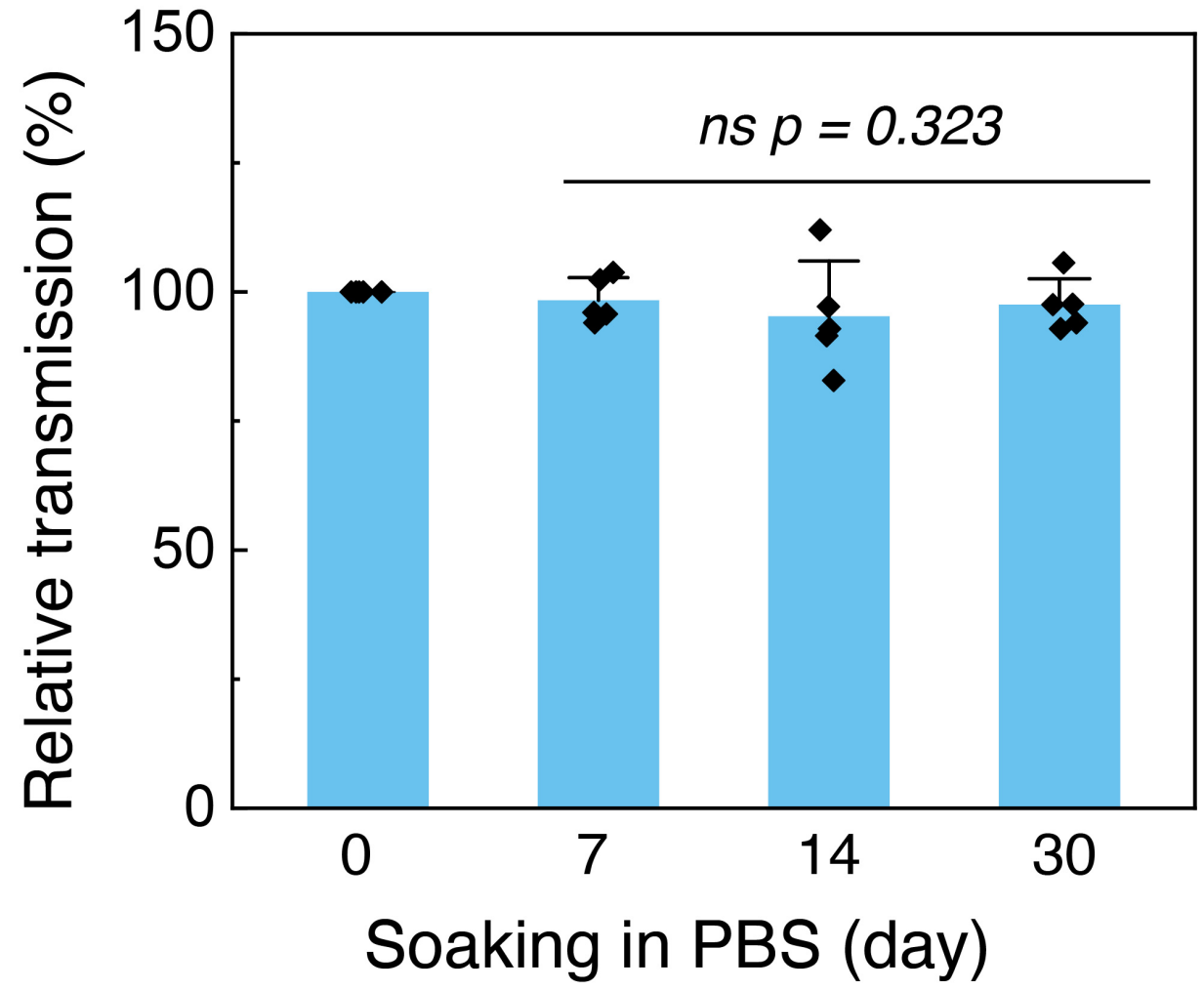

Supplementary Fig. 10 Stability of optical performance in TAHOF. Changes in light transmittance of TAHOFs samples during soaking in PBS for 0, 7, 14, and 30 days. Data are presented as mean ± SD, n = 5 independent samples. p values were determined by one-way ANOVA; ns, $p > 0.05$, not significant.

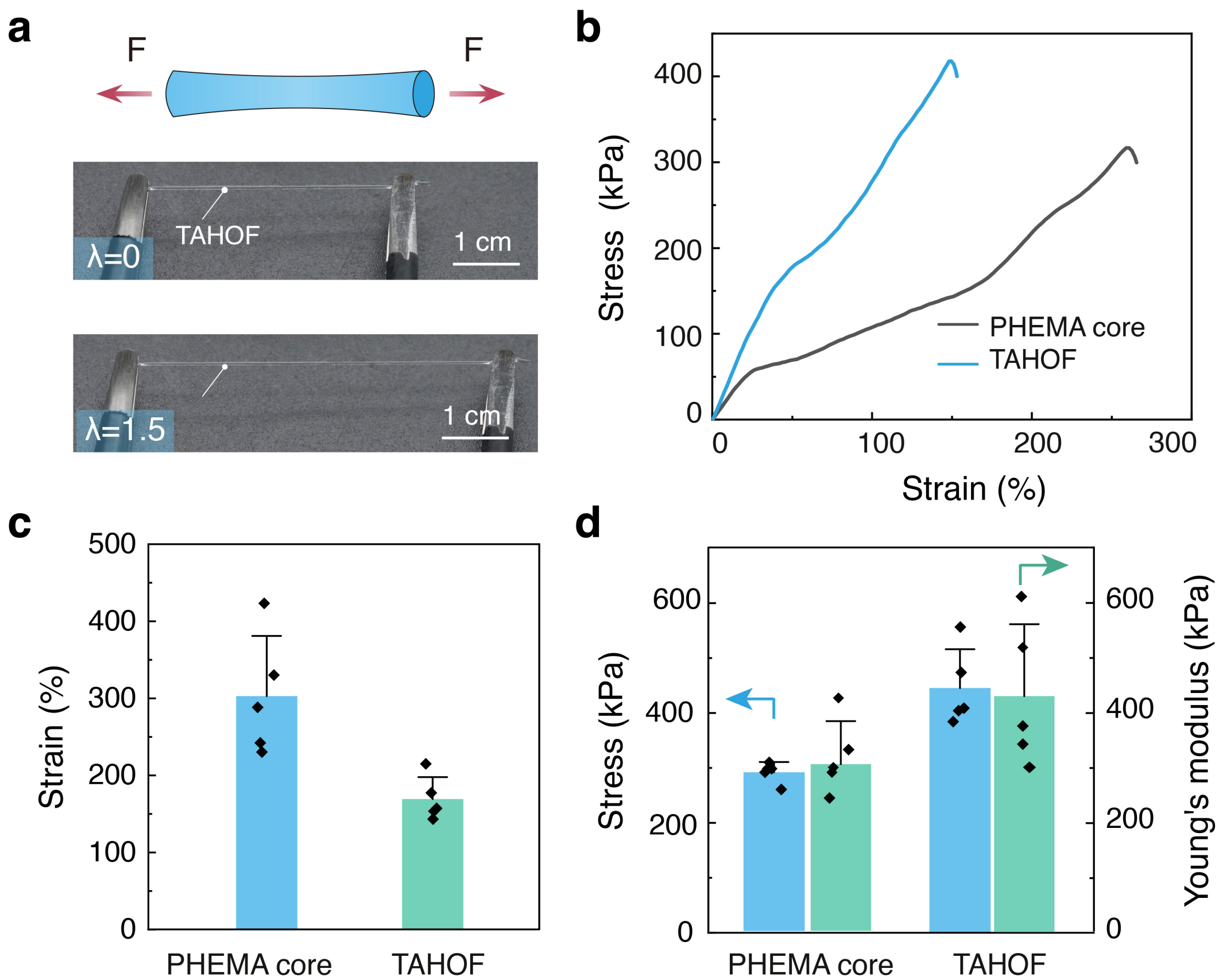

Supplementary Fig. 11 Mechanical properties of TAHOF at swelling equilibrium. **a** Photographs of TAHOFs under 0% and 150% uniaxial tensile strain. Scale bar: 1 cm. **b, c** Representative stress–strain curves **b** and corresponding maximum strain values **c** for the PHEMA hydrogel core and TAHOFs both measured at the swelling equilibrium state. **d** Comparison of maximum stress (left) and Young's modulus (right) between the two fiber types. Data in (**c** and **d**) are presented as mean ± SD (n = 5 independent samples).

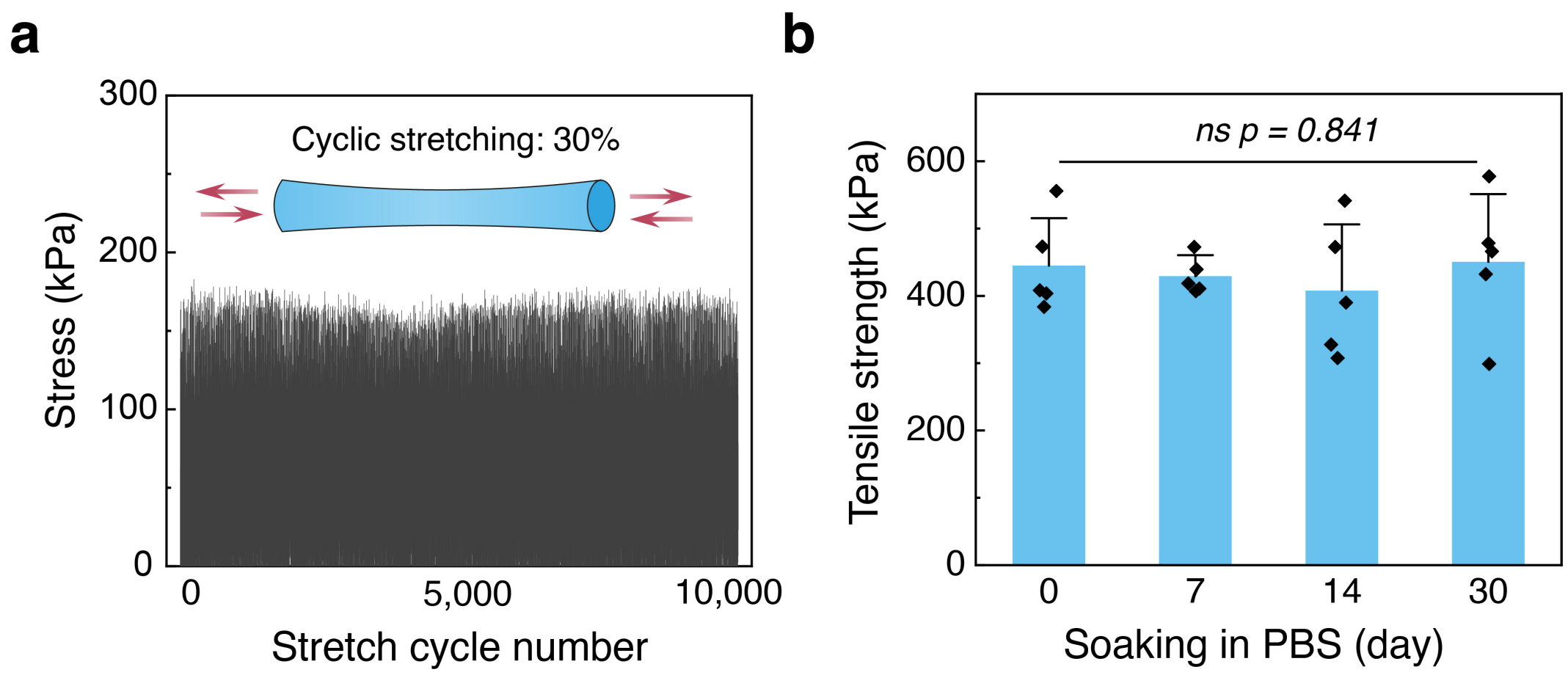

Supplementary Fig. 12 Mechanical stability of TAHOFs. a Representative cyclic stress profiles of TAHOFs samples subjected to 10,000-cycle tensile loading at 30% strain. **b** Evolution of maximal tensile strength over a 30-day incubation in PBS. Data are presented as mean ± SD (n = 5 independent samples). Statistical analysis was performed using one-way ANOVA; ns indicates no significant difference ($p > 0.05$).

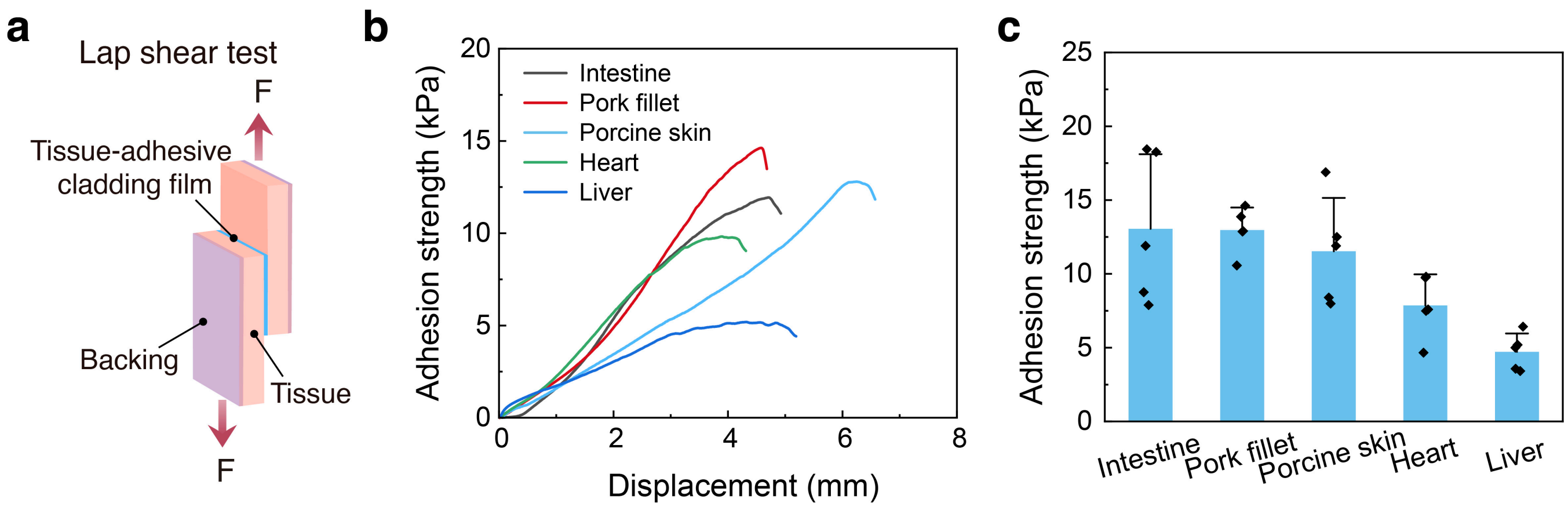

Supplementary Fig. 13 Adhesion performance of the adhesive cladding layer of TAHOF. a Schematic illustration of the lap-shear adhesion test. **b-c** Representative lap-shear curves of shear stress versus displacement (**b**) and quantitative summary of the adhesion strength (**c**) of the adhesive cladding layer on various biological tissues, including porcine intestine, fillet, skin, heart, and liver. Data in **c** are presented as mean ± SD, n = 5 independent samples.

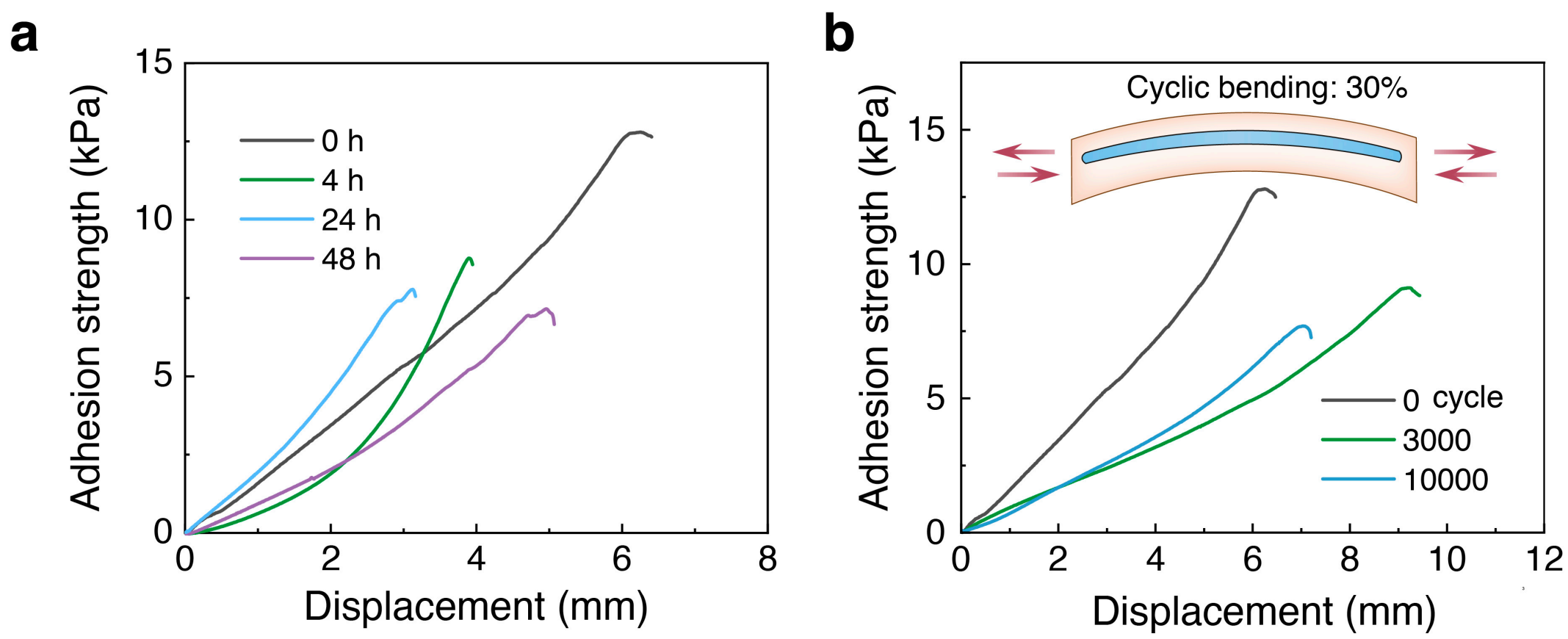

Supplementary Fig. 14 Tissue adhesion performance of TAHOFs. a Shear strength of adhered joints of porcine skins with the cladding films after soaking in PBS for 0, 4, 24, and 48 h. **b** Shear strength of adhered joints of porcine skins with the cladding films after 0, 3,000, and 10,000 cycles of 30% bending strain, following adhesion with the cladding film.

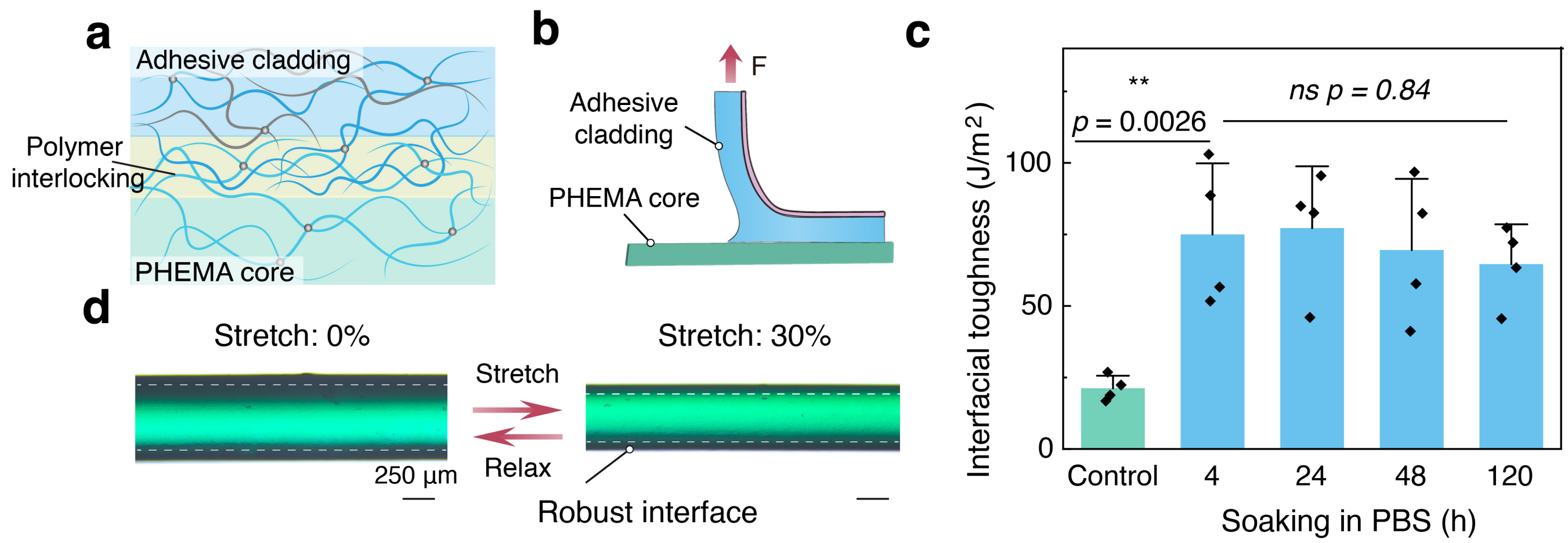

Supplementary Fig. 15 Robust interfacial adhesion between the PHEMA core and the tissue-adhesive cladding. **a** Schematic illustration of the polymer interlocking mechanism at the core-cladding interface. Acrylic acid monomers infiltrate the PHEMA core and are subsequently crosslinked under UV light to form a grafted poly(acrylic acid) (PAA) hydrogel layer. **b** Schematic of the 90° peel test setup used to measure interfacial toughness. **c** Interfacial toughness of the cladding hydrogel grafted onto the PHEMA core after soaking in PBS for 4, 24, 48, and 120 h. The control group represents a PVA hydrogel bonded to the PHEMA core without polymer interlocking. **d** Optical microscope images of a TAHOF before and after 30% tensile strain, showing that the core-cladding interface remains intact after deformation. Scale bar: 250 μm. Data are presented as mean ± SD (n = 5 independent samples).

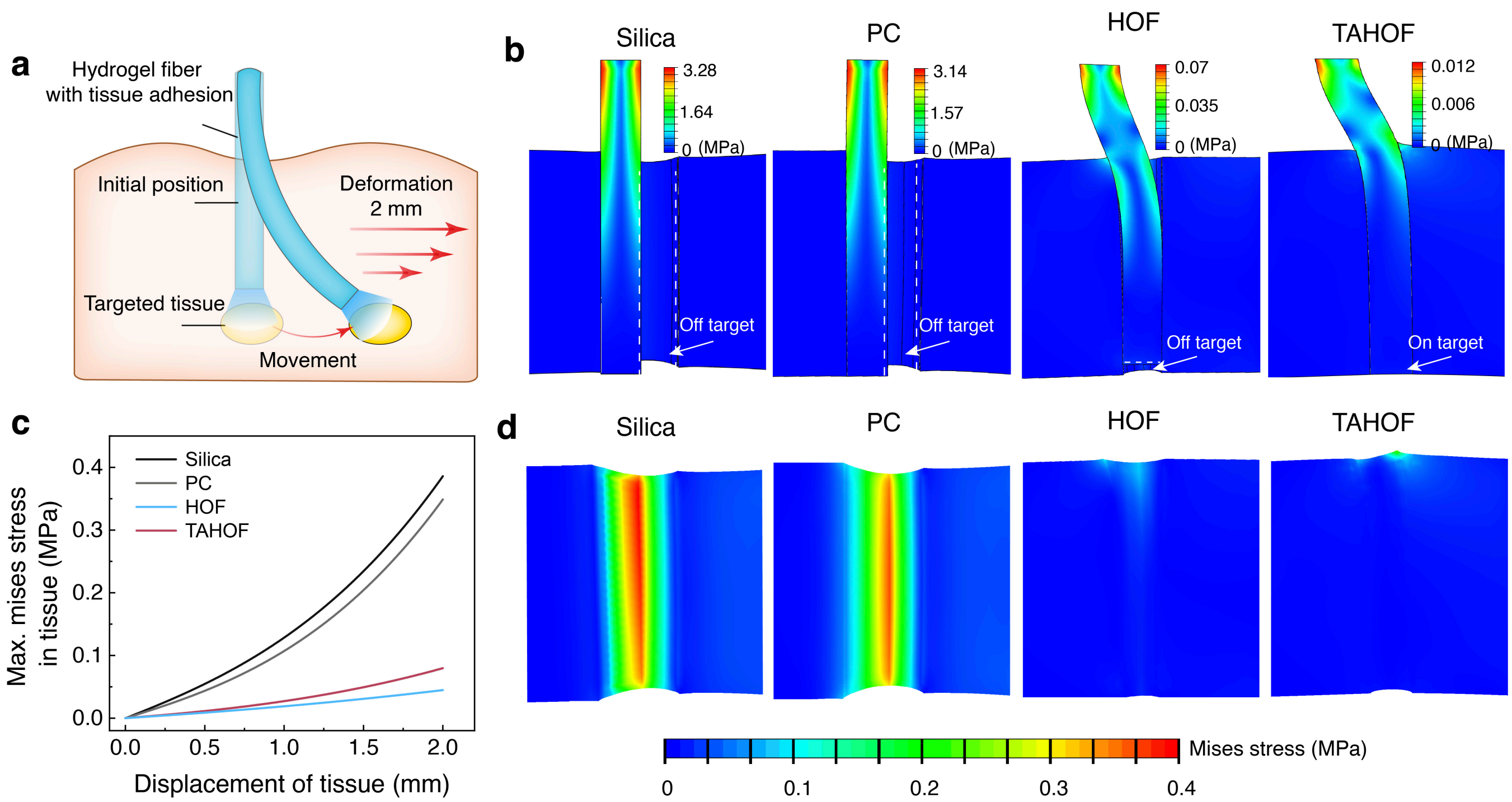

Supplementary Fig. 16 Mises stress analysis of implanted fibers and surrounding tissue under physiological motion simulation. **a** Finite element analysis framework models TAHOFs embedded in soft tissue undergoing 2 mm dynamic displacement, replicating respiratory, peristaltic, and volumetric deformation patterns. **b** Comparative von Mises stress profiles reveal material-dependent mechanical responses. **c** Quantification of the maximum von Mises stress in tissue containing each fiber type under displacement ranging from 0 to 2 mm. **d** Stress distribution maps showing von Mises stress in the surrounding tissue under 2 mm deformation for each fiber condition, illustrating differences in stress localization and intensity.

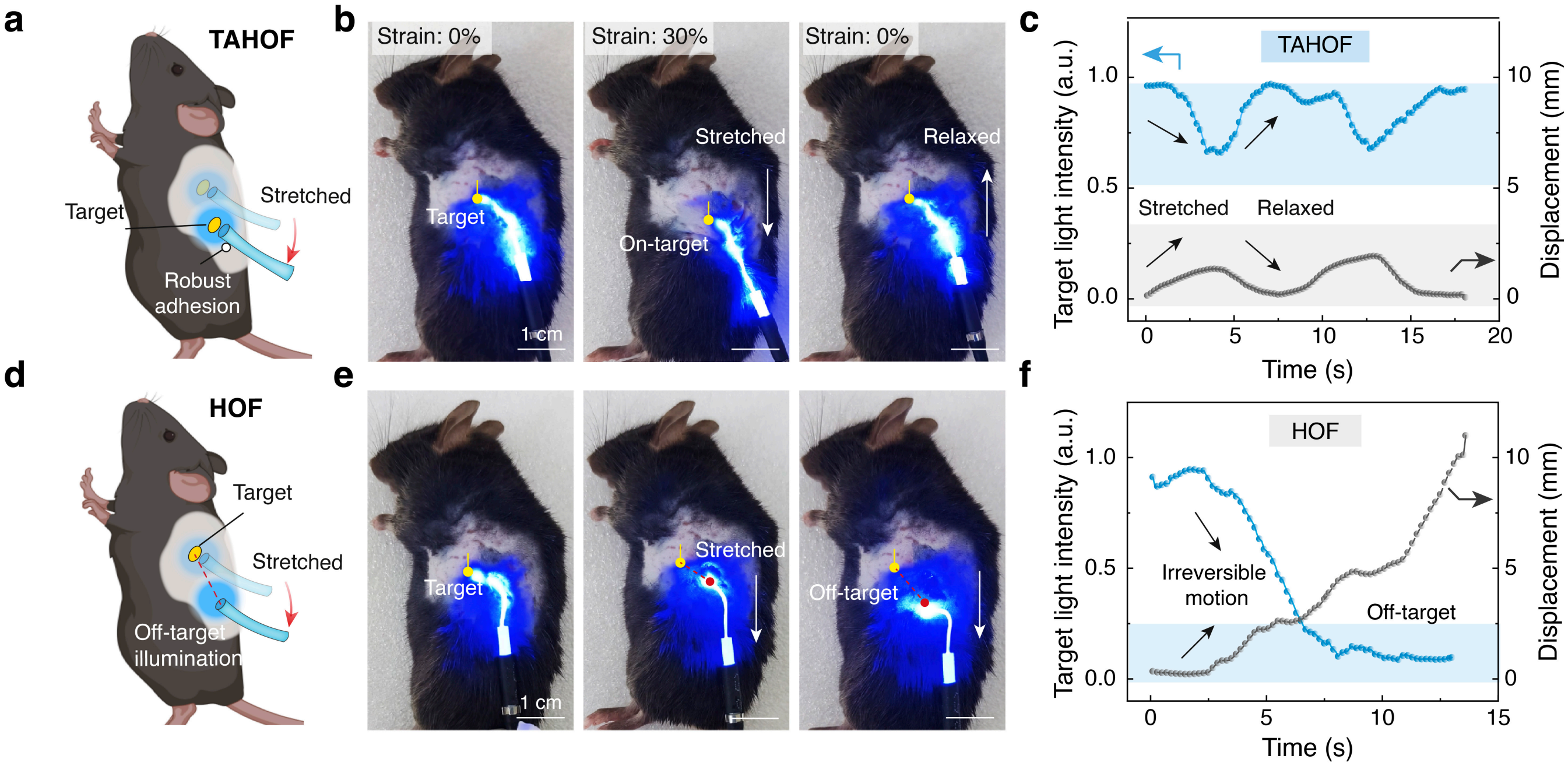

Supplementary Fig. 17 In vivo validation of tissue adhesion of TAHOF after implantation on the subcutaneous tissue of mice. a Schematic of light path deviation in TAHOF adhered to mouse subcutaneous tissue under stretching. **b** Light path tracking during 30% strain, demonstrating TAHOF's stable on-target illumination in mouse subcutaneous tissue (red: source outlet; yellow: target site). **c** Quantitative analysis of target-site light intensity and source-target displacement during strain for TAHOF. **d** Schematic of light path deviation in HOF adhered to subcutaneous tissue in mice upon stretching. **e** HOF exhibits light drift and off-target illumination under strain. **f** Quantitative analysis of target-site light intensity and displacement for HOF. The mouse model schematic of (**a**) and (**d**) are are created in BioRender. Chen, X. (2026) https://BioRender.com/9g29yc6.

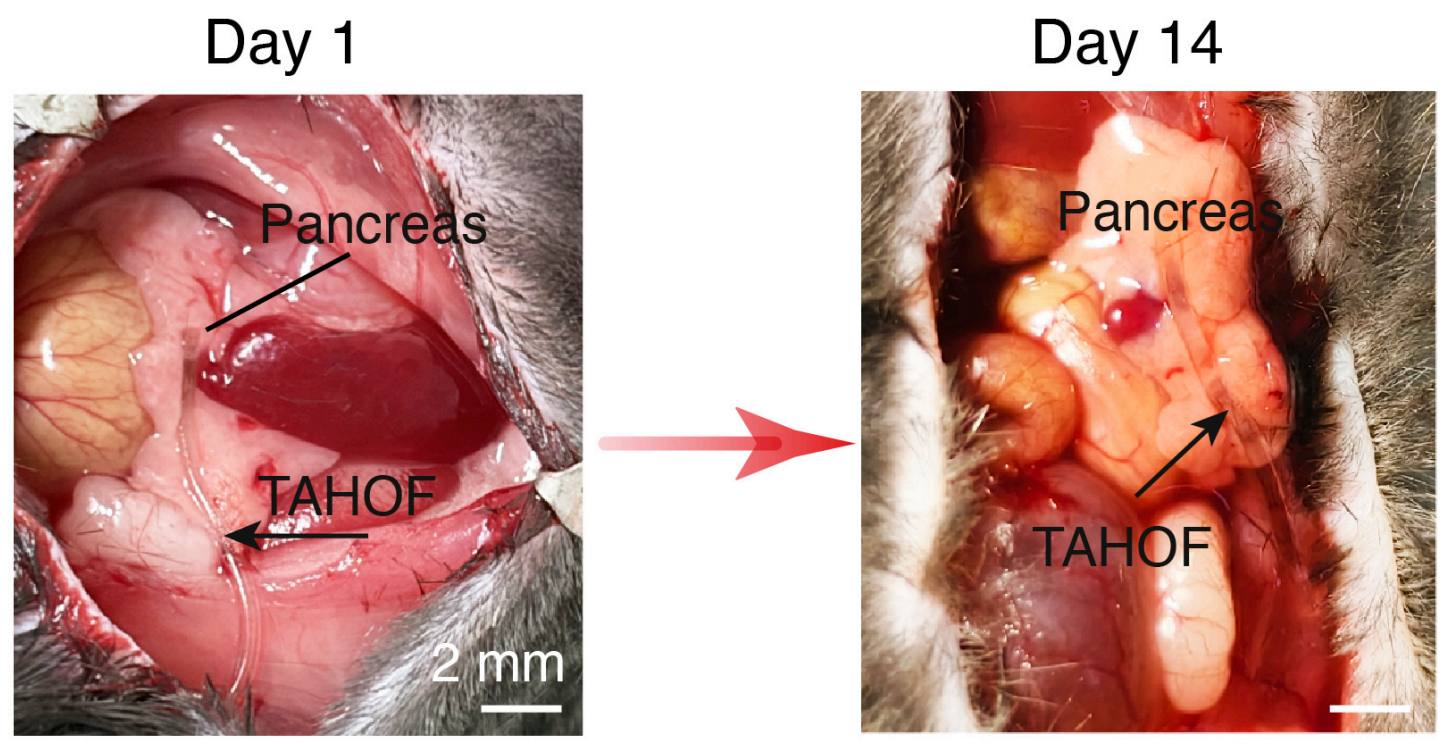

Supplementary Fig. 18 Representative photographs of TAHOFs on the pancreas at day 1 and day 14, showing persistent adhesion after chronic implantation. Scale bar 2 mm.

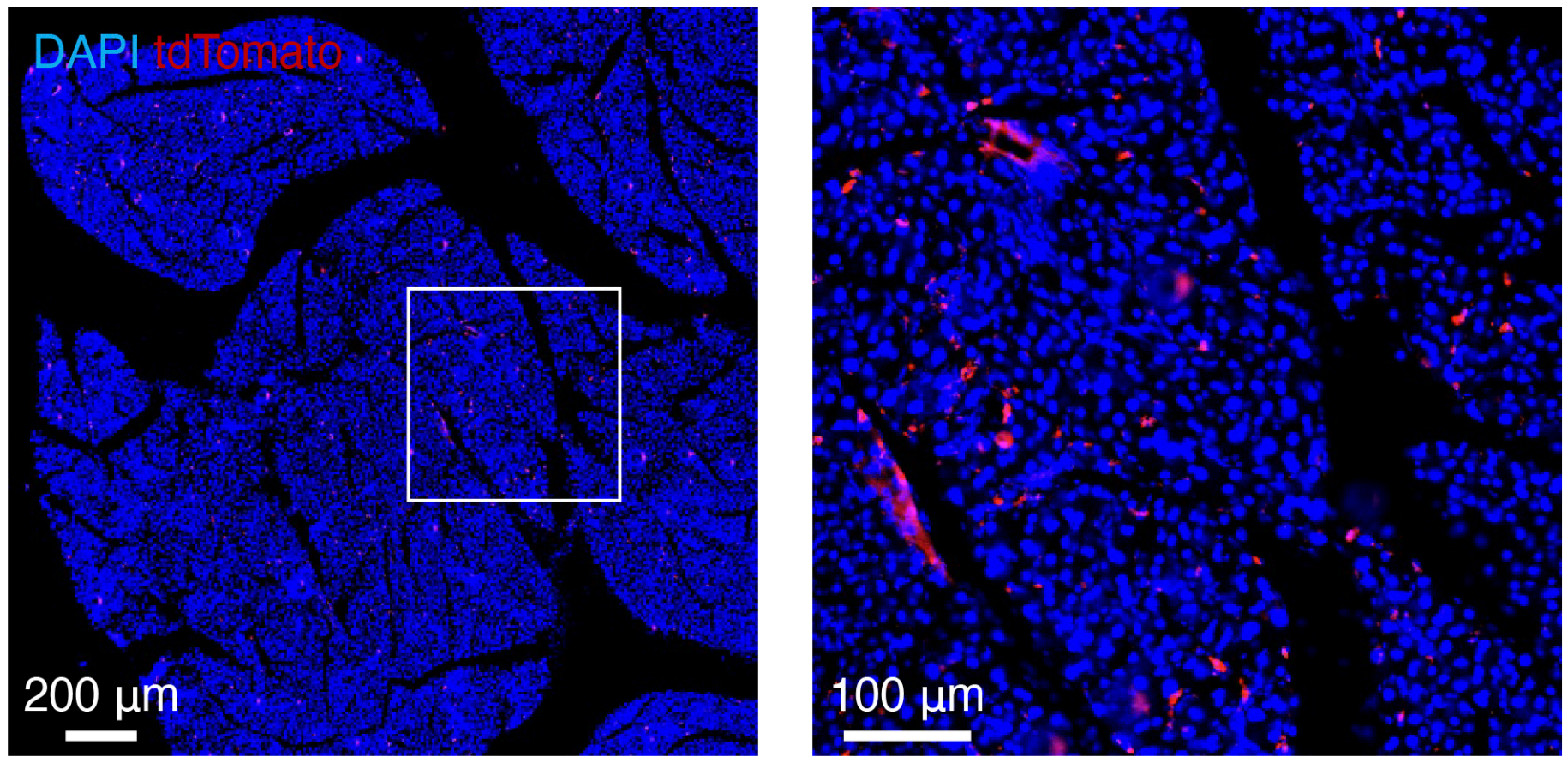

Supplementary Fig. 19 Histological validation of ChR2-tdTomato expression in pancreatic vagal cholinergic fibers in ChAT-ChR2 mice.

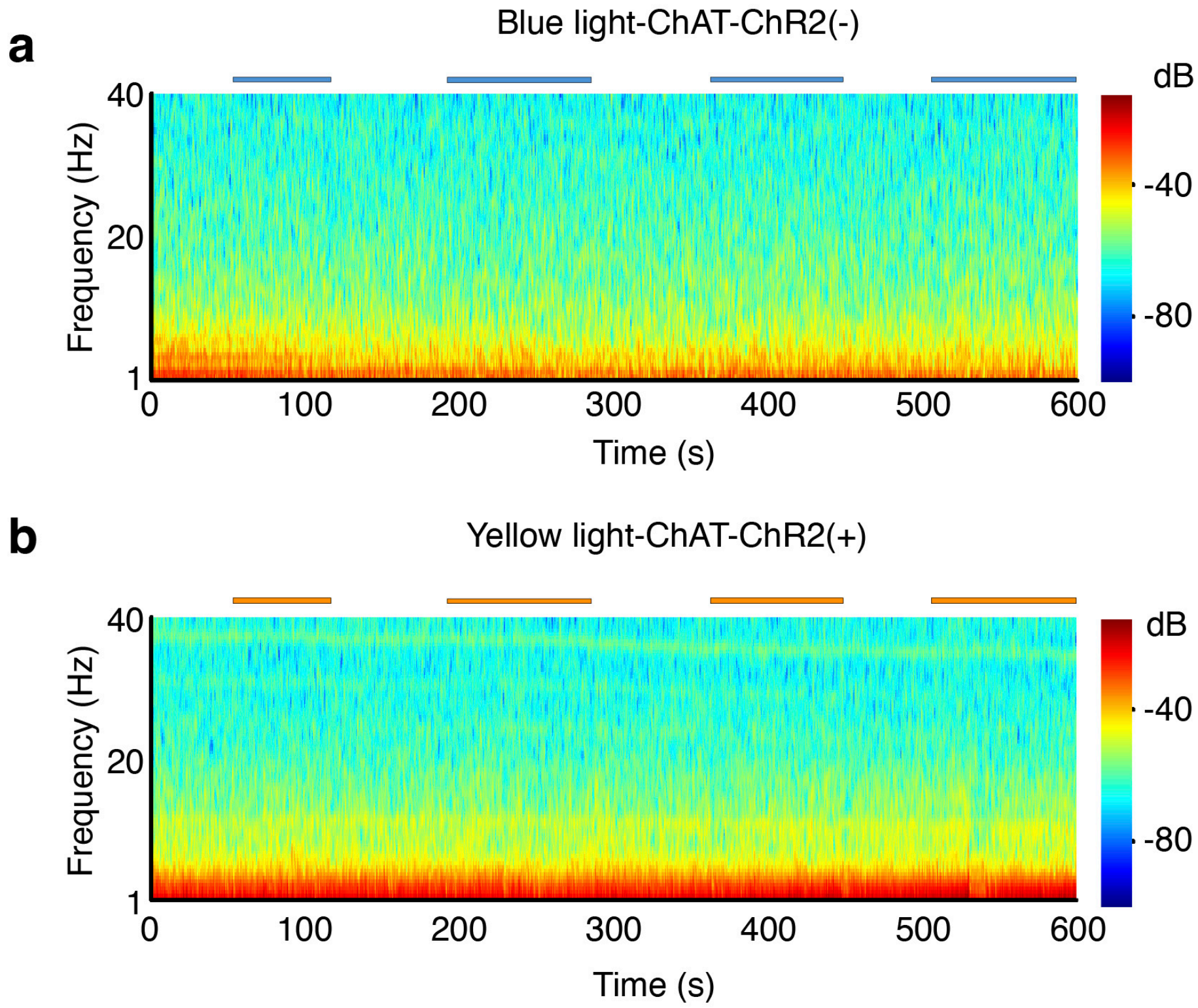

Supplementary Fig. 20 Spectrograms of local field potentials (LFPs) recorded from the subdiaphragmatic vagus nerve during optical stimulation. a, Spectrogram of LFPs recorded with a cuff electrode in ChR2-negative mice (ChAT-ChR2(-)) during pulsed blue-light stimulation (470 nm, 20 Hz, 5 ms) delivered through the TAHOF. **b,** Spectrogram of LFPs recorded with a cuff electrode in ChAT-ChR2(+) mice during pulsed yellow-light stimulation (589 nm, 20 Hz, 5 ms) delivered through the TAHOF.

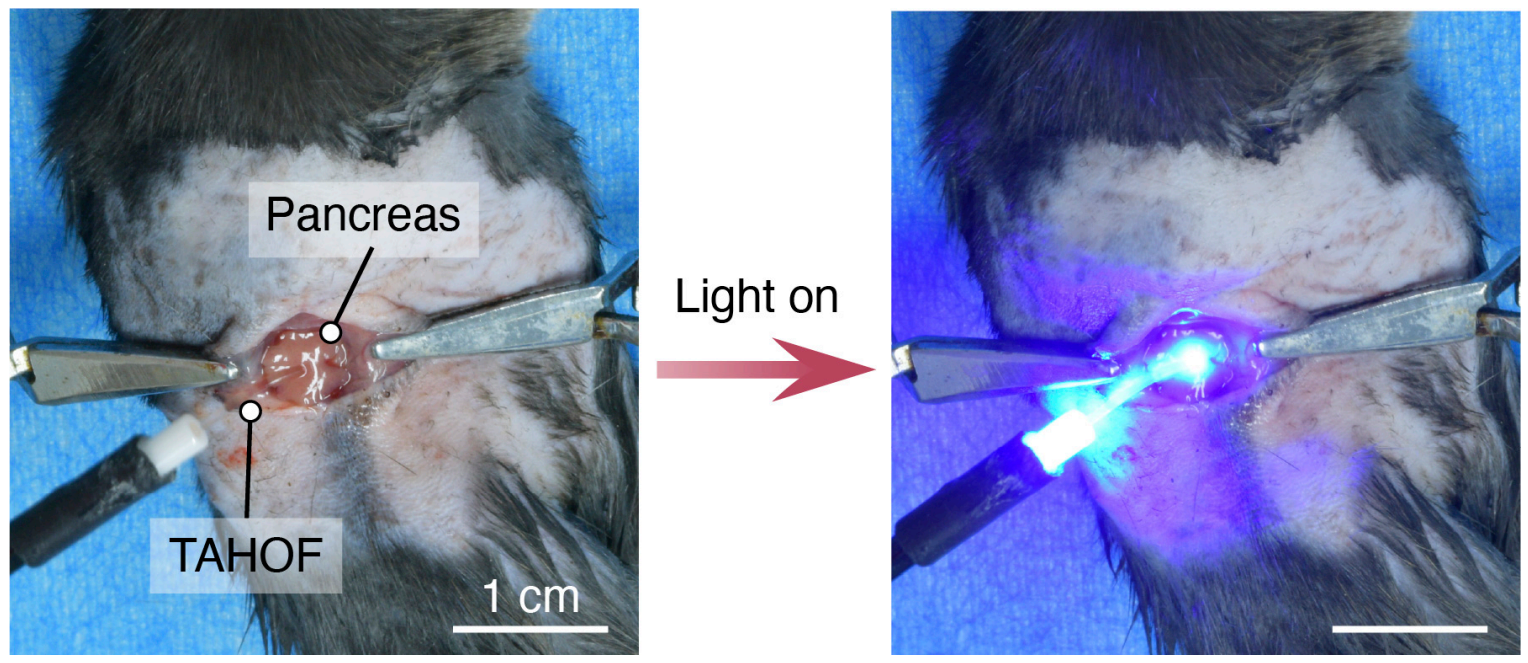

Supplementary Fig. 21 In situ optogenetic modulation through the TAHOF. Representative image of a TAHOF delivering 470 nm light to pancreatic tissue. Scale bar: 1 cm.

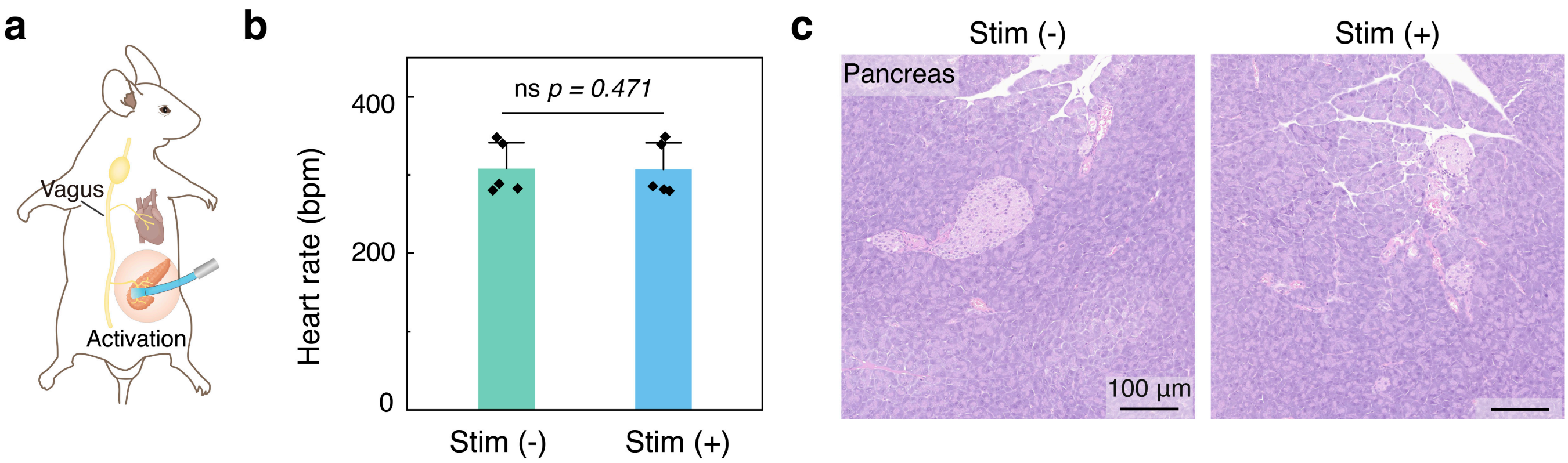

Supplementary Fig. 22 Phototoxicity evaluation of TAHOFs in ChAT-ChR2 mice. a Schematic illustration of light delivery from TAHOFs to the pancreatic branch of the vagus nerve. **b** Heart rate of ChAT-ChR2 mice before (Stim (–)) and during (Stim (+)) blue light stimulation (470 nm, 20 mW, 20 Hz, 5 ms) applied to the vagus nerve innervating the pancreas. **c** HE staining of pancreatic tissue collected before and after three consecutive days of light stimulation (Stim (+), 120 min per day). Scale bar: 100 µm. Data in **b** are presented as mean ± SD (n = 5 mice). Statistical significance was determined using a two-sided unpaired t-test; ns indicates no significant difference (*p* > 0.05).

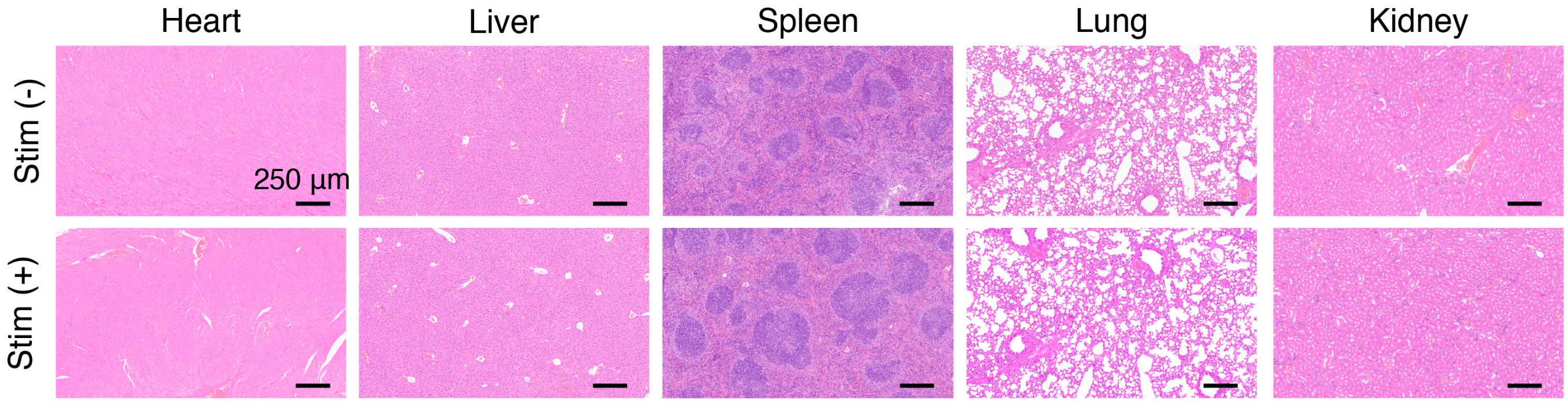

Supplementary Fig. 23 HE staining of major organs (heart, liver, spleen, lung, and kidney) from ChAT-ChR2 mice before (Stim (–)) and after three consecutive days of pancreatic-targeted optical stimulation (Stim (+), 470 nm, 20 mW, 20 Hz, 5 ms pulse, 120 min per day).

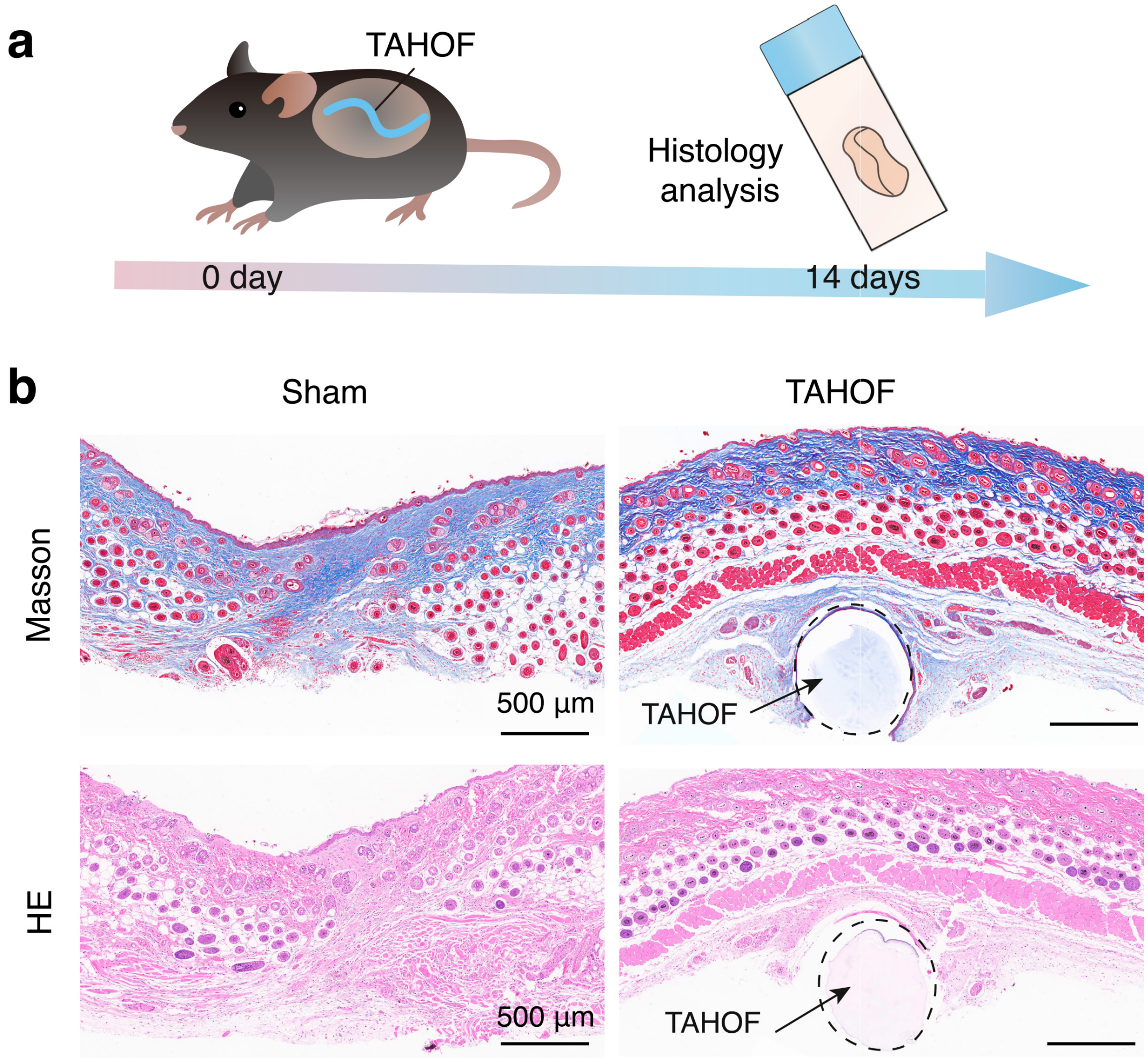

Supplementary Fig. 24 ***In vivo*** **biocompatibility of TAHOFs. a** Schematic illustration of the long-term subcutaneous implantation of TAHOFs in a mouse model. **b** Masson and HE staining of tissue sections surrounding the implanted TAHOFs on Day 14. Scale bar: 500 µm.

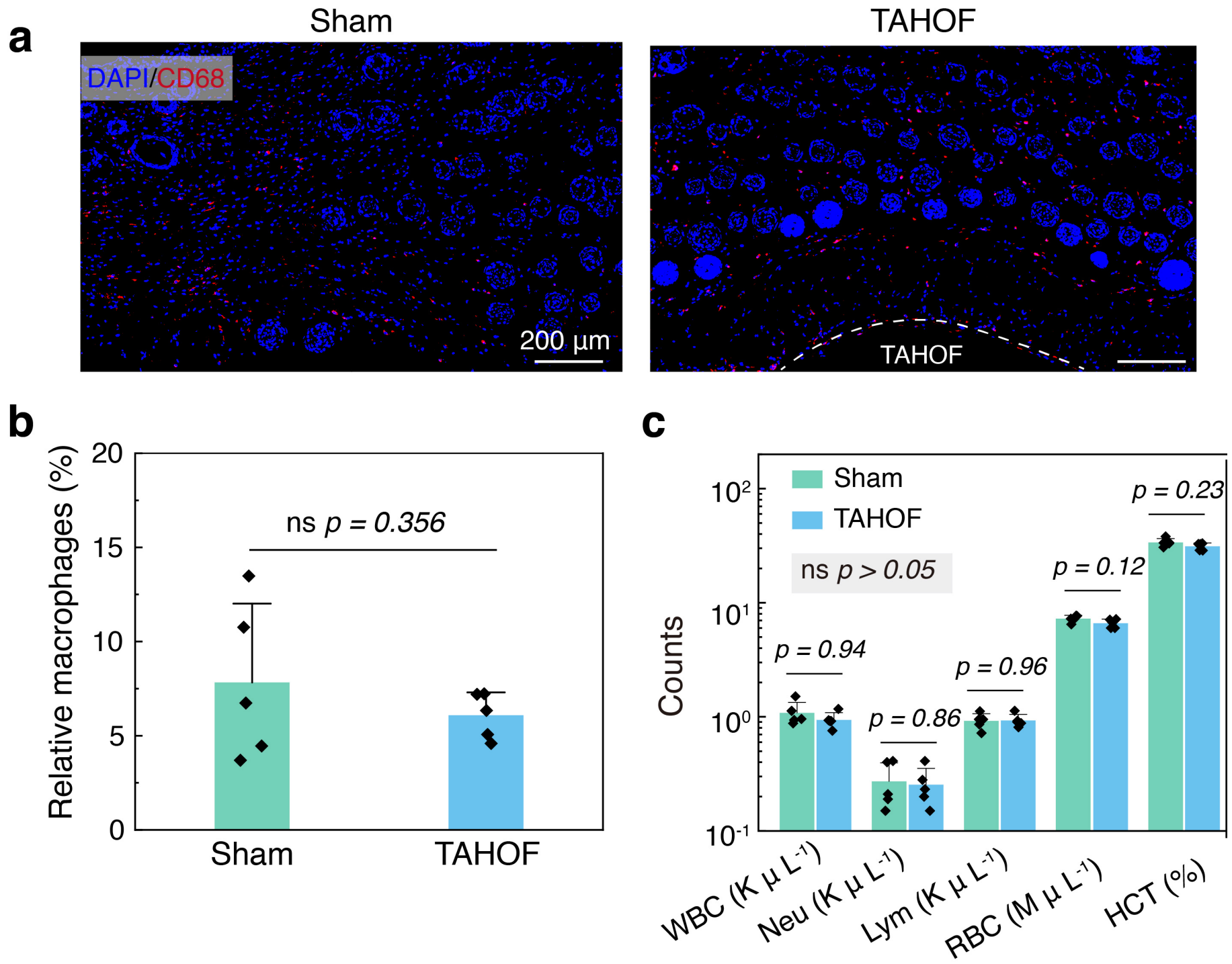

Supplementary Fig. 25 Inflammatory response to TAHOFs following subcutaneous implantation. **A** Immunostaining for CD68 (red, macrophage marker) in tissue sections surrounding to the implanted TAHOF on Day 14. Scale bar: 200 µm. **b** Quantification of $CD68^{+}$ macrophage area from the stained tissue sections on Day 14. **c** Complete blood count (CBC) analysis of sham and TAHOF-implanted mice 14 days post-implantation. WBC, white blood cell; Neu, neutrophil; Lym, lymphocyte; RBC, red blood cell; HCT, hematocrit. Data are presented as mean ± SD, $n = 5$ mice. p values were determined by two-sided t-test; ns, not significant; $^{*}p < 0.05$; $^{**}p < 0.01$; $^{***}p < 0.001$.

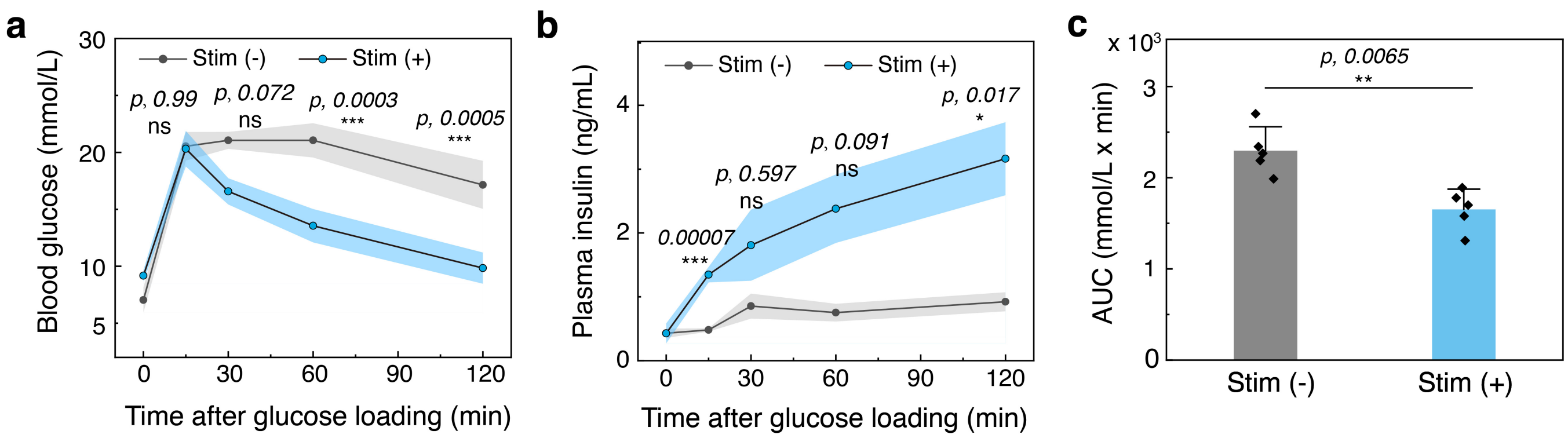

Supplementary Fig. 26 Optogenetic stimulation via TAHOF for modulating insulin secretion. a Blood glucose levels measured in ChAT-ChR2 mice during optogenetic stimulation (470 nm, 20 Hz, 5 ms) of the pancreatic cholinergic nerve fibers via TAHOFs, performed alFigurengside intraperitoneal glucose tolerance tests (2 g/kg). **b** Plasma insulin levels measured in the same animals during optical stimulation. **c** Area under the curve (AUC) of glucose concentrations over time corresponding to the data shown in **a**. Data in **a** and **b** are presented as mean ± SE; data in **c** as mean ± SD; *n* = 5 mice for all groups. Statistical significance was determined by two way repeated measures ANOVA followed by Bonferroni post hoc test (**a-b**) and two-sided unpaired t-tests (**c**). ns, not significant; **p* < 0.05; ***p* < 0.01; ****p* < 0.001.

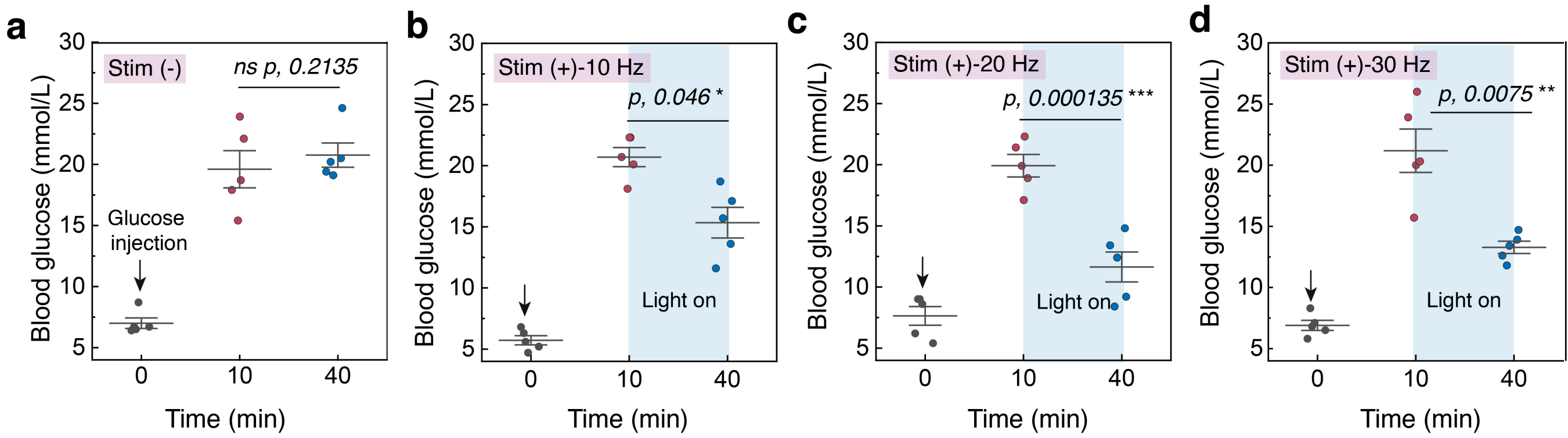

Supplementary Fig. 27 Optogenetic stimulation via TAHOF during glucose homeostasis experiments in ChAT-ChR2 mice. **a**, Blood glucose levels measured for 40 min following intraperitoneal glucose injection (2 g/kg). **b-d,** Blood glucose levels measured during optogenetic stimulation of vagal fibers innervating the pancreas via TAHOFs (470 nm, 5 ms) at 10 Hz (**b**), 20 Hz (**c**), and 30 Hz (**d**). Intraperitoneal glucose injection (2 g/kg) was performed at 0 min, and optical stimulation was applied from 10 to 40 min. Blood glucose measurements were obtained from 5 mice. Statistical significance and p-values were determined using two-sided paired t-test. ns, not significant; $^*p < 0.05$; $^{**}p < 0.01$; $^{***}p < 0.001$.

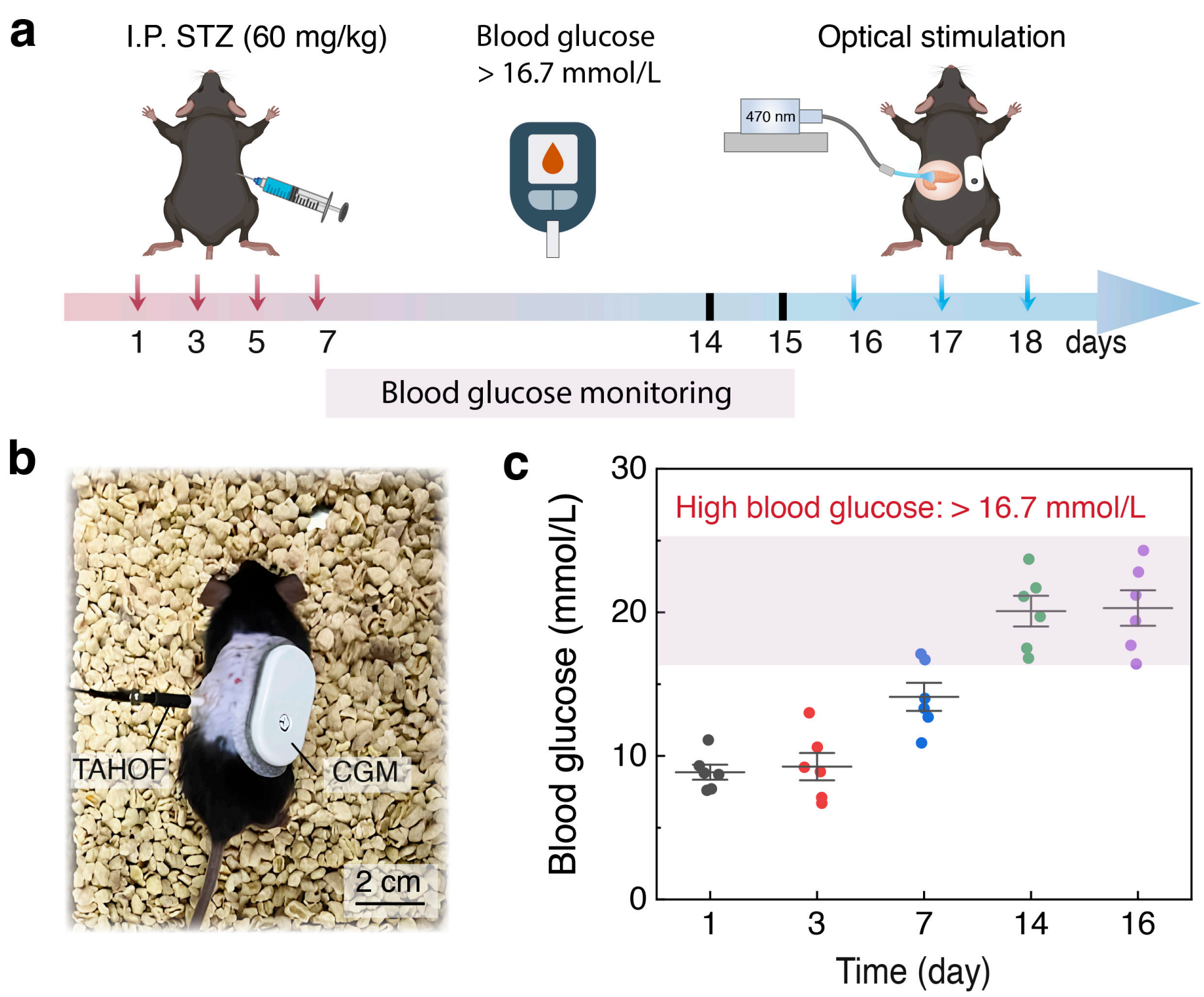

Supplementary Fig. 28 Real-time glycemic control using TAHOFs-mediated optogenetic therapy in diabetic ChAT-ChR2 mice. **a** Schematic illustration of the experimental timeline for assessing optogenetic stimulation via TAHOFs to modulate insulin secretion in streptozotocin (STZ)-induced hyperglycemic mice. **b** Photograph of a diabetic ChAT-ChR2 mouse implanted with a TAHOFs and monitored using a commercial CGM system (SIBIONICS®). Scale bar: 2 cm. **c** Fasted blood glucose levels recorded throughout the experimental period using a handheld glucometer (FreeStyle Libre, Abbott). Data are presented as mean ± SE (*n* = 6 mice). The mouse model schematic of (**a**) is created in BioRender. Chen, X. (2026) https://BioRender.com/9g29yc6.

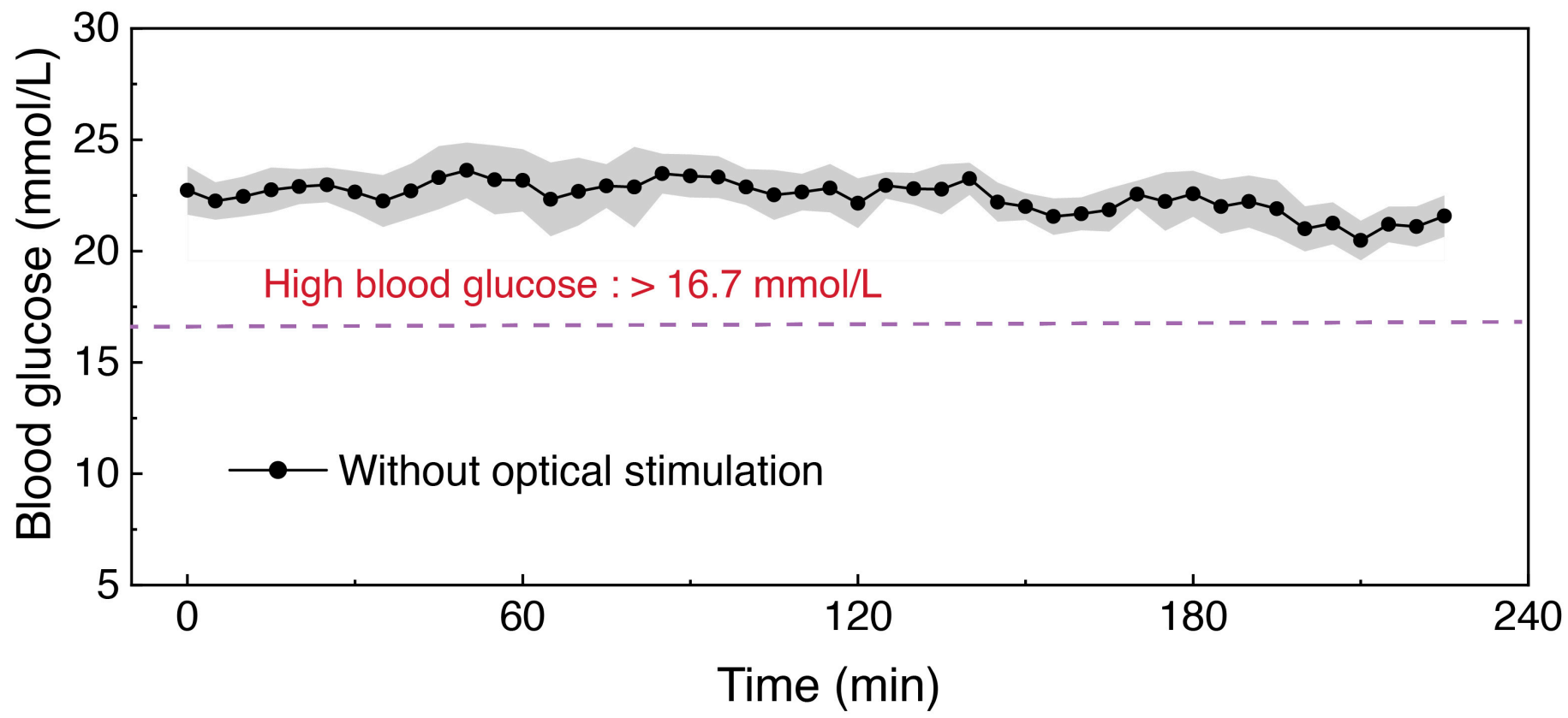

Supplementary Fig. 29 Blood glucose monitoring in diabetic mice following TAHOFs implantation without optical stimulation. Blood glucose levels were continuously recorded over 225 minutes using the implanted CGM system in ChAT-ChR2 diabetic mice that received TAHOFs implantation without optical stimulation. n = 4 mice per group. Data are presented as mean ± SE.

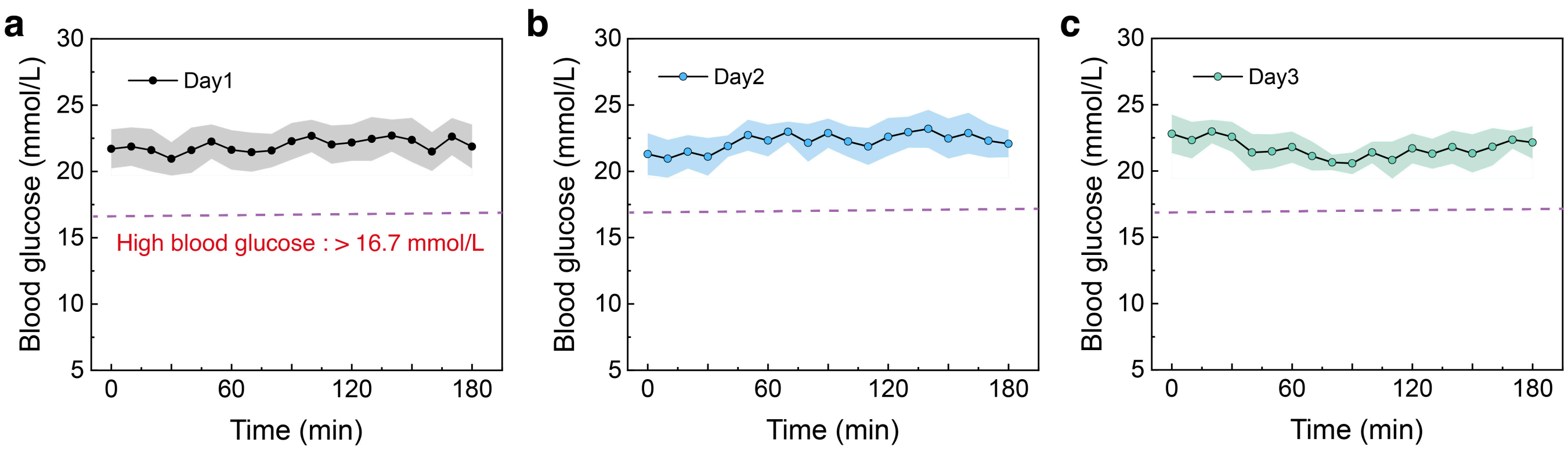

Supplementary Fig. 30 Short-term blood glucose monitoring after TAHOF implantation in diabetic mice. a-c Continuous blood glucose monitoring for 180 min per day over 3 consecutive days using a CGM system in ChAT-ChR2 diabetic mice implanted with TAHOFs but without optical stimulation. n = 4 mice per group. Data are presented as mean ± SE.

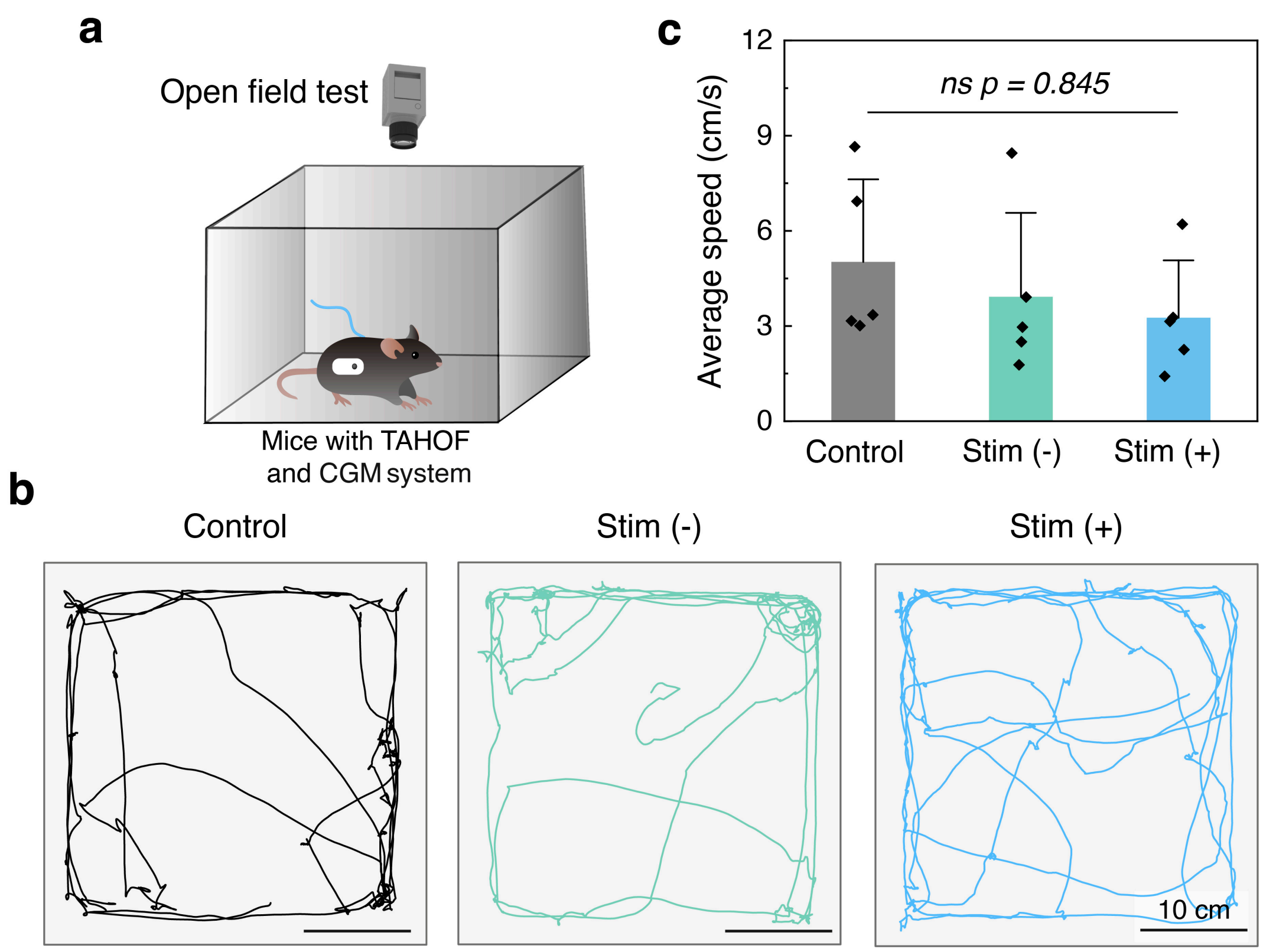

Supplementary Fig. 31 Behavioral evaluation of motor function upon implantation TAHOFs and sensors in ChAT-ChR2 mice. a Schematic of the open field test (OFT) used to assess locomotor activity following implantation of TAHOFs and sensors. **b** Representative locomotor traces with or without optical stimulation (470 nm, 20 Hz, 5 ms) via the TAHOFs; control mice received sham surgery without fiber implantation. **c** Quantification of the average locomotor speed of mice during 3min of optical stimulation in the OFT. Data are presented as mean ± SD (n = 5 mice). Statistical significance was determined using one-way ANOVA; ns, not significant ($p > 0.05$).

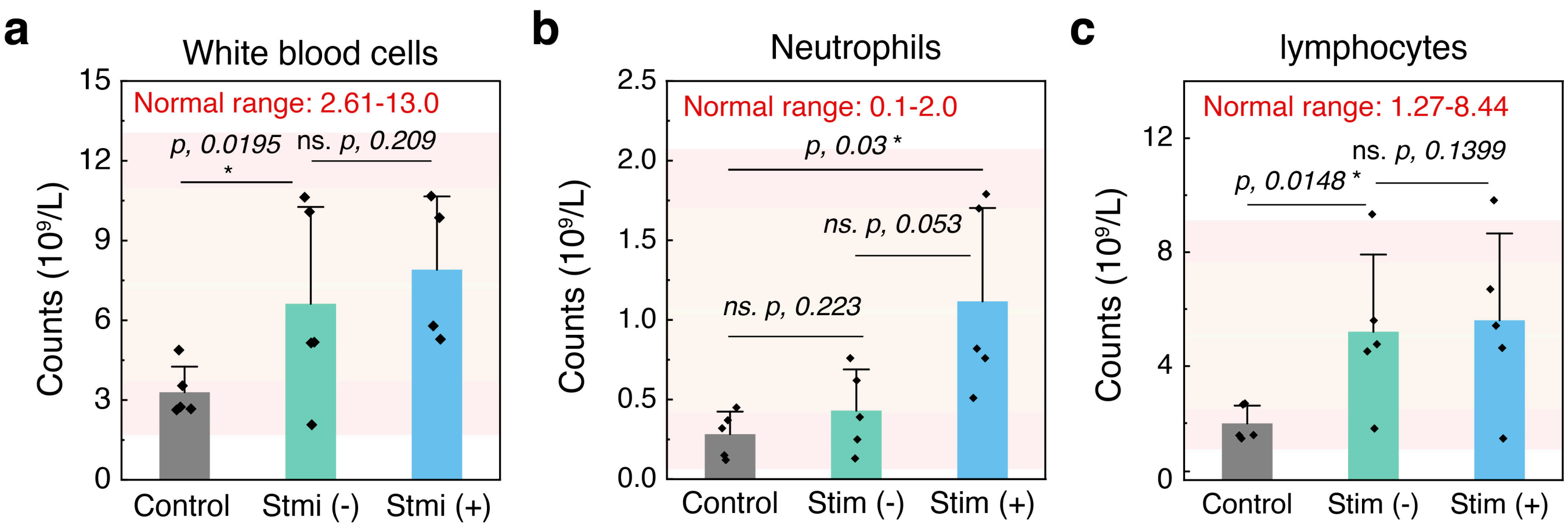

Supplementary Fig. 32 Routine blood parameters following implantation of TAHOFs in ChAT-ChR2 diabetic mice. a-c Quantification of white blood cells **a**, neutrophils **b**, and lymphocytes **c** in ChAT-ChR2 diabetic mice after TAHOFs implantation and daily optical stimulation (470 nm, 20 mW, 20 Hz, 5ms, 90 minutes per day) for three consecutive days. Rectangles indicate the physiological reference ranges for each parameter. Data are presented as mean ± SD ($n = 5$ mice). Statistical significance was determined by two-sided unpaired t-tests. ns, not significant; $*p < 0.05$.

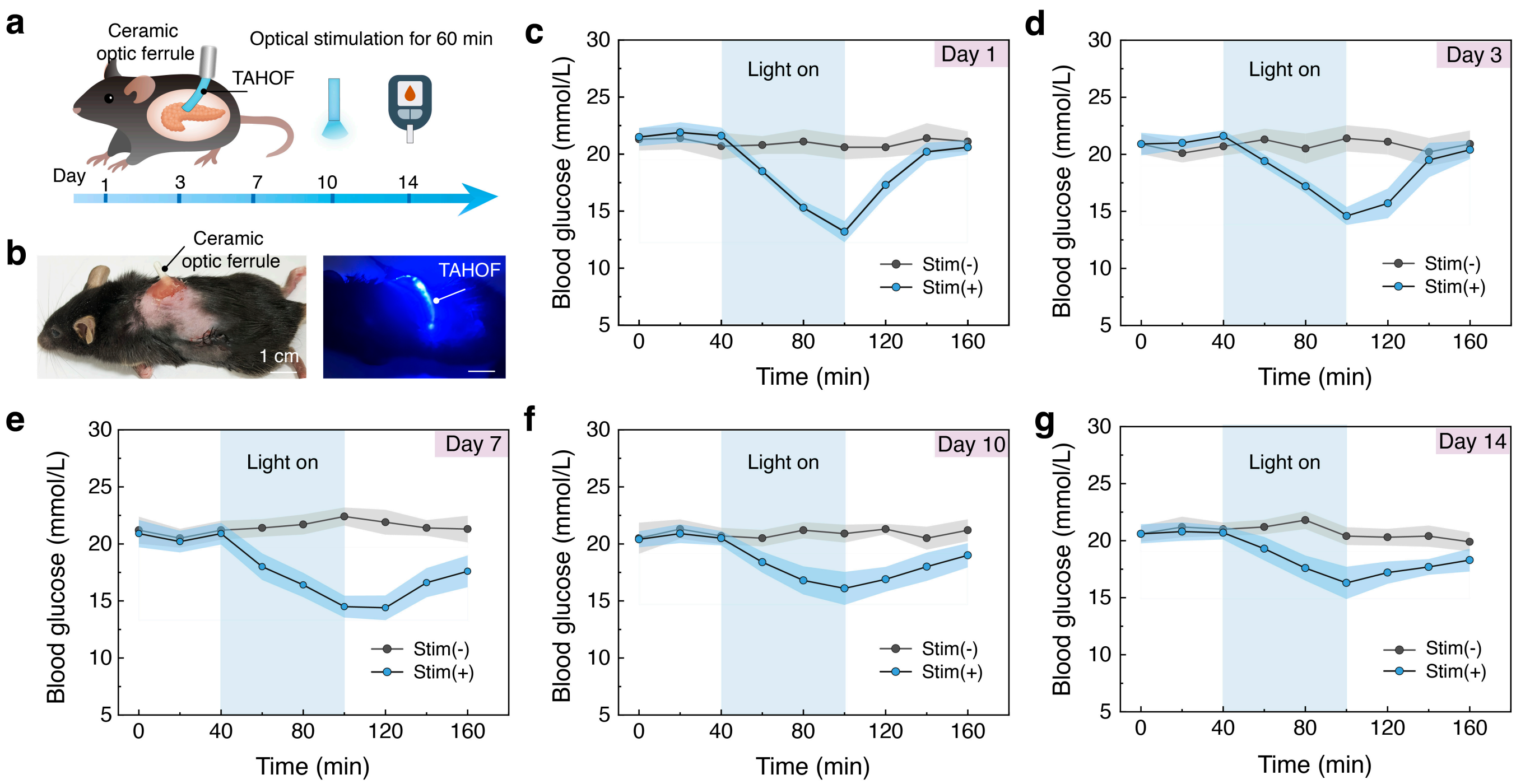

Supplementary Fig. 33 Chronic optogenetic stimulation via implanted TAHOF in diabetic ChAT-ChR2 mice. a Schematic of pancreatic TAHOF implantation and the chronic stimulation protocol. Optical stimulation was applied for 60 min at post-implantation day 1, 3, 7, 10, and 14. Blood glucose was measured every 20 min by tail-vein sampling using glucose test strips. **b** Photograph of a TAHOF-implanted mouse. The fiber was fixed along the subcutaneous implantation path by the adhesive cladding, and the proximal end was coupled to a ceramic ferrule secured to the dorsal side of the mouse with dental cement. Scale bar, 1 cm. **c-g** Blood glucose levels during 60-min optogenetic stimulation (470 nm, 20 mW, 20 Hz, 5 ms) via TAHOF at post-implantation day 1 (**c**), day 3 (**d**), day 7 (**e**), day 10 (**f**), and day 14 (**g**). Mice implanted with TAHOF but without optical stimulation served as controls. Data are presented as mean ± SE, with 5 mice per group.

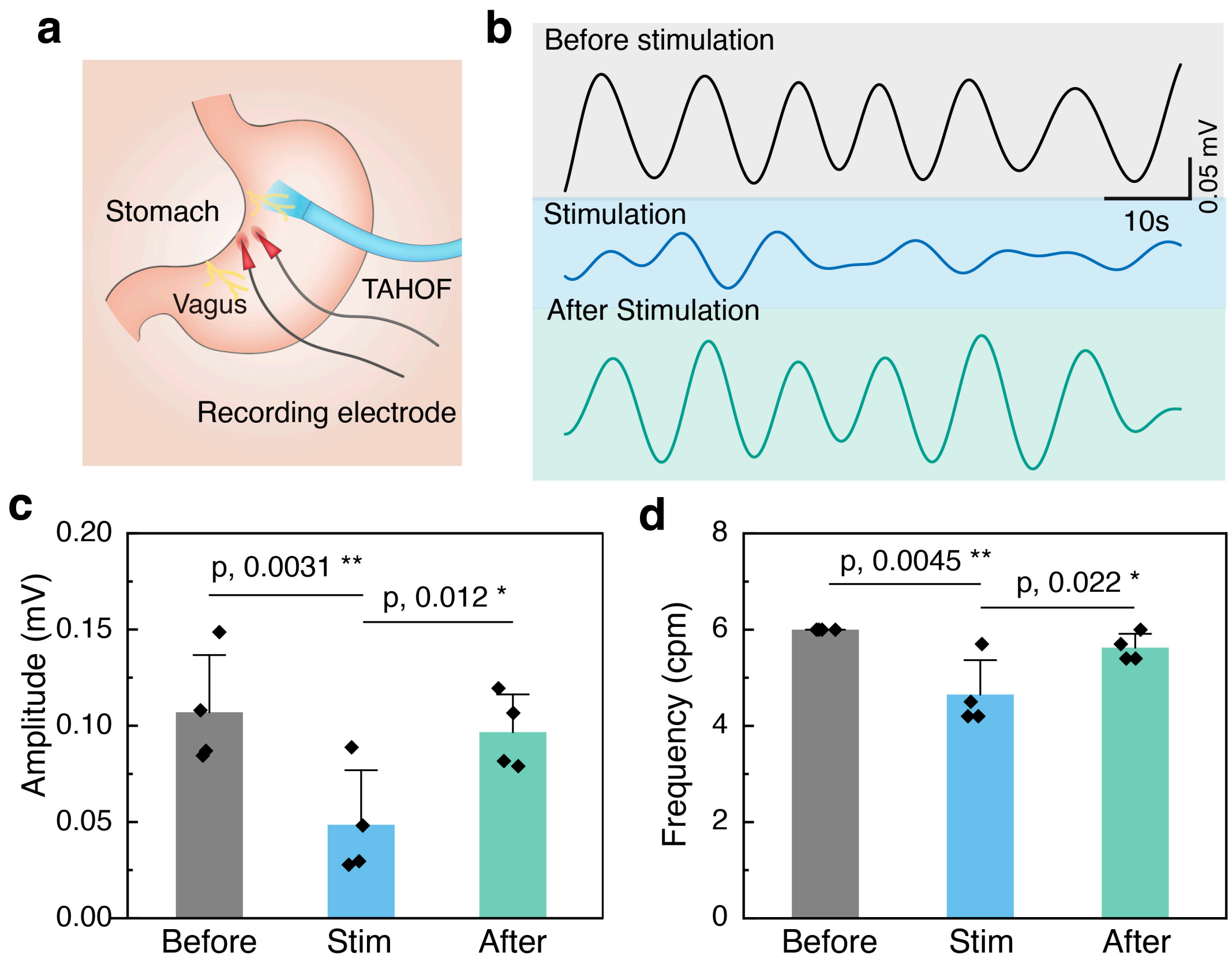

Supplementary Fig. 34 Gastric optogenetic modulation via TAHOF. a, Schematic illustration of TAHOF implantation on the stomach for optical stimulation and electrophysiological recording of gastric slow waves in ChAT-ChR2 mice. **b,** Representative ~70 s gastric slow-wave recording during optical stimulation (470 nm, 20 Hz, 5 ms), together with the continuous wavelet transform of the filtered signals (frequency passband: 0.08-0.13 Hz). **c,d,** Quantitative analysis of gastric slow-wave amplitude (**c**) and frequency (**d**) before, during, and after optical stimulation via TAHOF. Data are presented as mean ± SD, with 4 mice per group. Statistical significance and p-values were determined using two-sided unpaired t-test. ns, not significant; *$p < 0.05$, **$p < 0.01$.

Supplementary Table 1. Mechanical parameters for different optical fiber materials for the FEA models (HOF and TAHOF hydrogels are in the fully swollen state).

Material	Young's modulus (G)	Poisson's ratio (ν)	Shear modulus (E)	Diameter (μm)
Silica fiber	50000 MPa	0.17	21400 MPa	650
PC fiber	2380 MPa	0.37	868 MPa	650
HOF/TAHOF	0.485 MPa	0.46	0.166 MPa	650
Skin	0.12 MPa	0.5	0.04 MPa	/

Supplementary References

1. Wang, L., *et al.* Ultrasoft and Highly Stretchable Hydrogel Optical Fibers for In Vivo Optogenetic Modulations. *Adv. Opt. Mater.* **6**, 1800427 (2018).
2. Jacques, S.L. Optical properties of biological tissues: a review. *Phys. Med. Biol.* **58**, R37-61 (2013).
3. Chansoria, P., Etter, E.L. & Nguyen, J. Regenerating dynamic organs using biomimetic patches. *Trends. Biotechnol.* **40**, 338-353 (2022).
4. Park, S., *et al.* Adaptive and multifunctional hydrogel hybrid probes for long-term sensing and modulation of neural activity. *Nat. Commun.* **12**, 3435 (2021).
5. Yang, Y., *et al.* Mechanically-Compliant Magnetoelectric Sutures for Wound Management. *Adv Funct Mater* **35**, 2510353 (2025).
6. Kawana, Y., *et al.* Author Correction: Optogenetic stimulation of vagal nerves for enhanced glucose-stimulated insulin secretion and beta cell proliferation. *Nat Biomed Eng* **8**, 1501 (2024).
7. Fontaine, A.K., *et al.* Optogenetic stimulation of cholinergic fibers for the modulation of insulin and glycemia. *Sci Rep* **11**, 3670 (2021).
8. Lin, J., *et al.* Wireless Bioelectronics for In Vivo Pressure Monitoring with Mechanically-Compliant Hydrogel Biointerfaces. *Adv Mater* **36**, e2400181 (2024).
9. Liu, X., *et al.* Fatigue-resistant hydrogel optical fibers enable peripheral nerve optogenetics during locomotion. *Nat. Methods* **20**, 1802-1809 (2023).
10. Chen, X., *et al.* Hydrogel bioadhesives with extreme acid - tolerance for gastric perforation repairing. *Adv Funct Mater* **32**, 2202285 (2022).
