# Supplementary material for: Tissue-adhesive hydrogel optical fiber for peripheral optogenetic neuromodulation
Source: Nat Commun. 2026 Jun 29;17:8101. doi: 10.1038/s41467-026-74831-1 (PMC13458817; doi:10.1038/s41467-026-74831-1)
Supplement: Supplementary file 2 [file 41467_2026_74831_MOESM2_ESM.pdf]

Description of Additional Supplementary File

Supplementary Movie 1. Optical propagation in TAHOFs under uniaxial tensile strain. This video demonstrates light transmission through the TAHOFs during uniaxial stretching from 0% to 90% strain. While light output decreases with increasing strain, the fiber consistently maintains high transmission efficiency, highlighting its robust optical performance under mechanical deformation.

Supplementary Movie 2. Tissue adhesion performance of TAHOFs under cyclic tensile strain. This video shows a TAHOFs, stained with green food dye for visualization, conformally adhered to excised esophageal tissue. The fiber-tissue interface maintains robust adhesion during repeated 30% uniaxial tensile strain, demonstrating stable anchorage under dynamic deformation.

Supplementary Movie 3. Light delivery from TAHOFs during deformation in subcutaneous tissue of mice. This video compares light delivery from hydrogel fibers with and without tissue adhesion when implanted subcutaneously in mice and subjected to repeated tensile stretching. TAHOFs maintains near target illumination throughout deformation, as the light-emitting tip closely follows the initial alignment during strain. In contrast, non-adhesive hydrogel fiber (HOF) exhibits visible displacement relative to the tissue, resulting in off-target illumination under identical stretching conditions.

Supplementary Movie 4. In vivo implantation of TAHOFs on pancreatic tissue. This video shows TAHOFs conformally adhered to the pancreas in a ChAT-ChR2 mouse. When pulling force is applied to the ceramic ferrule coupled to the fiber, no detachment from the pancreatic surface is observed, demonstrating the robust tissue adhesion stability of TAHOFs under dynamic body environment.

Supplementary Movie 5. Behavioral assessment of ChAT-ChR2 diabetic mouse following TAHOF and CGM implantation. This video shows that the diabetic ChAT-ChR2 mouse does not exhibit any obvious locomotor impairment in the test cage after implantation of the TAHOF and the CGM system. Despite the CGM weighing approximately 4 g-equivalent to 15-20% of the mouse’s body weight-the animal maintains normal mobility. No noticeable changes in locomotor behavior are observed during optical stimulation (470 nm, 20 mW, 20 Hz, 5 ms) compared to the pre-stimulation condition.
