# Supplementary material for: Tissue-adhesive hydrogel optical fiber for peripheral optogenetic neuromodulation
Source: Nat Commun. 2026 Jun 29;17:8101. doi: 10.1038/s41467-026-74831-1 (PMC13458817; doi:10.1038/s41467-026-74831-1)
Supplement: Supplementary file 3 [file 41467_2026_74831_MOESM8_ESM.pdf]

nature portfolio

Corresponding author(s): Ji Liu; Yi Lu; Cunjiang Yu

Last updated by author(s): May 12, 2026

Reporting Summary

Nature Portfolio wishes to improve the reproducibility of the work that we publish. This form provides structure for consistency and transparency in reporting. For further information on Nature Portfolio policies, see our Editorial Policies and the Editorial Policy Checklist.

Statistics

For all statistical analyses, confirm that the following items are present in the figure legend, table legend, main text, or Methods section.

n/a	Confirmed	
☐	☒	The exact sample size (*n*) for each experimental group/condition, given as a discrete number and unit of measurement
☐	☒	A statement on whether measurements were taken from distinct samples or whether the same sample was measured repeatedly
☐	☒	The statistical test(s) used AND whether they are one- or two-sided<br>*Only common tests should be described solely by name; describe more complex techniques in the Methods section.*
☒	☐	A description of all covariates tested
☐	☒	A description of any assumptions or corrections, such as tests of normality and adjustment for multiple comparisons
☐	☒	A full description of the statistical parameters including central tendency (e.g. means) or other basic estimates (e.g. regression coefficient) AND variation (e.g. standard deviation) or associated estimates of uncertainty (e.g. confidence intervals)
☐	☒	For null hypothesis testing, the test statistic (e.g. F, t, r) with confidence intervals, effect sizes, degrees of freedom and P value noted<br>*Give P values as exact values whenever suitable.*
☒	☐	For Bayesian analysis, information on the choice of priors and Markov chain Monte Carlo settings
☒	☐	For hierarchical and complex designs, identification of the appropriate level for tests and full reporting of outcomes
☒	☐	Estimates of effect sizes (e.g. Cohen's *d*, Pearson's *r*), indicating how they were calculated

Our web collection on statistics for biologists contains articles on many of the points above.

Software and code

Policy information about availability of computer code

Data collection	Data were collected using instrument-associated acquisition software for optical, mechanical, imaging, MRI, electrophysiological, behavioral, and glucose-monitoring measurements. Optical output and propagation measurements were recorded using calibrated power meters, including an LP10 power meter (Sanwa, Japan) and an inline power meter (PM16-122, Thorlabs). FDTD simulations were performed in Ansys Lumerical FDTD (2020 r2). Mechanical testing, including tensile, cyclic tensile, and adhesion measurements, was performed using a horizontal tensile testing system equipped with a 4.4 N load cell (CellScale, Canada). Electrophysiological recordings were acquired using a Plexon multi-channel neural acquisition system (OmniPlex Release 1.20). Behavioral videos were recorded and analyzed using EthoVision XT software (version 16.0.1536, Noldus). MRI data were acquired using a 9.4 T preclinical MRI system. Blood glucose was monitored using FreeStyle Libre (Abbott) and SiBiONICS continuous glucose monitoring systems. No custom code was used for primary data collection.
Data analysis	Data were analyzed using Origin 2024, MATLAB 2018b (MathWorks), ABAQUS 2016, NeuroExplorer (Version 5.437, Plexon), EthoVision XT (version 16.0.1536, Noldus), and Olympus OlyVIA 3.3 and ImageJ (version 1.52p). FDTD (2020 r2) simulation analysis. No custom algorithm central to the conclusions of the study was developed.

For manuscripts utilizing custom algorithms or software that are central to the research but not yet described in published literature, software must be made available to editors and reviewers. We strongly encourage code deposition in a community repository (e.g. GitHub). See the Nature Portfolio guidelines for submitting code & software for further information.

Data

Policy information about availability of data

All manuscripts must include a data availability statement. This statement should provide the following information, where applicable:

- Accession codes, unique identifiers, or web links for publicly available datasets
- A description of any restrictions on data availability
- For clinical datasets or third party data, please ensure that the statement adheres to our policy

All data supporting the findings of this study are available within the article and its supplementary materials. The source data generated in this study have been deposited in the Zenodo database under accession code 10.5281/zenodo.20109627 (https://doi.org/10.5281/zenodo.20109627). Additional raw data are available from the corresponding author upon reasonable request.

Research involving human participants, their data, or biological material

Policy information about studies with human participants or human data. See also policy information about sex, gender (identity/presentation), and sexual orientation and race, ethnicity and racism.

Reporting on sex and gender	Not applicable. This study did not involve human participants, human data, or human biological material.
Reporting on race, ethnicity, or other socially relevant groupings	Not applicable. This study did not involve human participants, human data, or human biological material.
Population characteristics	Not applicable. This study did not involve human participants.
Recruitment	Not applicable. No human participants were recruited.
Ethics oversight	Not applicable. This study did not involve human participants, human data, or human biological material.

Note that full information on the approval of the study protocol must also be provided in the manuscript.

Field-specific reporting

Please select the one below that is the best fit for your research. If you are not sure, read the appropriate sections before making your selection.

☒ Life sciences ☐ Behavioural & social sciences ☐ Ecological, evolutionary & environmental sciences

For a reference copy of the document with all sections, see nature.com/documents/nr-reporting-summary-flat.pdf

Life sciences study design

All studies must disclose on these points even when the disclosure is negative.

Sample size	The statistical analysis was conducted at least four times (n≥4) for each sample. No statistical methods were used to predetermine sample sizes; however, our sample sizes are consistent with those reported in previous studies (Yohei K et al., 2023 Nature Biomedical Engineering; Siyuan Rao et al., 2024 Nature Communications). All sample sizes can be found in the figure legends.
Data exclusions	No data were excluded from the analyses.
Replication	More than four independent experiments were performed in every experiment to ensure that the experimental results were reliable. Detailed information can be found in the figure legends.
Randomization	Animals were randomly assigned to control and experimental groups.
Blinding	Experimenters were blinded to control and experimental groups during both data acquisition and analysis.

Reporting for specific materials, systems and methods

We require information from authors about some types of materials, experimental systems and methods used in many studies. Here, indicate whether each material, system or method listed is relevant to your study. If you are not sure if a list item applies to your research, read the appropriate section before selecting a response.

Materials & experimental systems

n/a	Involved in the study
☐	☒ Antibodies
☒	☐ Eukaryotic cell lines
☒	☐ Palaeontology and archaeology
☐	☒ Animals and other organisms
☒	☐ Clinical data
☒	☐ Dual use research of concern
☒	☐ Plants

Methods

n/a	Involved in the study
☒	☐ ChIP-seq
☒	☐ Flow cytometry
☒	☐ MRI-based neuroimaging

Antibodies

Antibodies used	1. Recombinant Anti-CD68 antibody (Rabbit mAb), Servicebio, Cat. No. GB153109-100, 1:200<br><br>2. Alexa Fluor 488-conjugated goat anti-rabbit secondary antibody, Jackson ImmunoResearch, Cat. No. 111-545-144, 1:1000
Validation	The antibodies were validated by the manufacturers.<br>Recombinant Anti-CD68 antibody (Rabbit mAb, Servicebio,GB153109-100), https://www.servicebio.cn/goodsdetail?id=24307<br>Alexa Fluor 488-conjugated goat anti-rabbit secondary antibody (Jackson ImmunoResearch,111-545-144), https://www.jacksonimmuno.com/catalog/products/111-545-144

Animals and other research organisms

Policy information about studies involving animals; ARRIVE guidelines recommended for reporting animal research, and Sex and Gender in Research

Laboratory animals	ChAT-ChR2 mice were generated by crossing ChAT-Cre mice (Jackson Laboratory, stock no. 018957) with Ai27D reporter mice (Jackson Laboratory, stock no. 012567). Male ChAT-Cre mice and ChAT-ChR2 mice (6-10 weeks old) and C57BL/6J wild-type mice (6-10 weeks old, Charles River Laboratories) were used for experiments.
Wild animals	Not applicable. This study did not involve wild animals.
Reporting on sex	Only male mice were used in this study. Sex was assigned based on external anatomical characteristics and supplier or breeding records. Sex-based analyses were not performed because the study was designed to evaluate the optical, mechanical, adhesive, and neuromodulatory performance of TAHOFs rather than sex-dependent biological effects. Male mice were selected to improve the stability of the STZ-induced hyperglycemic model and to reduce variability associated with estrous-cycle-related hormonal fluctuations in female mice.
Field-collected samples	Not applicable. This study did not involve field-collected samples.
Ethics oversight	All animal procedures were approved by the Ethics Committee for Animal Research of the Shenzhen Institutes of Advanced Technology, Chinese Academy of Sciences, and were conducted in accordance with institutional guidelines. The ethics approval number is SIAT-IACUC-221209-NS-LY-A2232.

Note that full information on the approval of the study protocol must also be provided in the manuscript.

Plants

Seed stocks	Not applicable. This study did not involve seed stocks or plant materials.
Novel plant genotypes	Not applicable. This study did not generate or use novel plant genotypes.
Authentication	Not applicable. This study did not involve plant materials requiring authentication.
