# Supplementary material for: Tissue-adhesive hydrogel optical fiber for peripheral optogenetic neuromodulation
Source: Nat Commun. 2026 Jun 29;17:8101. doi: 10.1038/s41467-026-74831-1 (PMC13458817; doi:10.1038/s41467-026-74831-1)
Supplement: Supplementary file 4 [file 41467_2026_74831_MOESM9_ESM.pdf]

nature portfolio

Peer Review File

Tissue-adhesive Hydrogel Optical Fiber for Peripheral Optogenetic Neuromodulation

Corresponding Author: Professor Ji Liu

This file contains all reviewer reports in order by version, followed by all author rebuttals in order by version.

Version 0:

Reviewer comments:

Reviewer #1

(Remarks to the Author)
1. While tissue adhesive light-delivery devices are an exciting concept, the novelty in TAHOFs presented in this work is rather low. There is abundant literature on hydrogel optical fibers as well as bio-adhesive hydrogel coatings. The authors have merged previously reported approaches in this work without introducing any new chemistry and/or materials approach.
2. How are the TAHOFs optically coupled to Silica fiber for light delivery? This is a high mechanical mismatch interface which can be prone to failure. The authors should describe in detail about this junction as well as its mechanical stability.
3. Experimental in-vivo validation of tissue adhesion of TAHOFs and control optical fibers is not convincing. The authors demonstrate the adhesiveness of TAHOFs in an acutely implanted animal by mechanically pulling on the light-coupling fiber [Fig. 3f-k]. This scenario is rather unusual, exaggerated, and very unlikely to occur in a freely moving animal. To convincingly prove the adhesive nature of TAHOFs compared to controls in a native physiological context, the authors should chronically implant these fibers (without silica coupling) for 2 weeks and then perform CT imaging on these mice to locate the fibers. The authors can add a CT contrast agent in their hydrogels to help visualize the fibers under CT. It is important to convincingly prove the readers that large displacement indeed takes place in control hydrogel optical fibers.
4. Validation of ChR2 expression in pancreatic vagal afferents is lacking.
5. “The implanted TAHOF delivered blue light pulses (473 nm, 20 mW, 20 Hz, 5 ms) to the subdiaphragmatic vagus nerve via an adjacent electrode array, inducing robust, timelocked local field potentials (LFPs) at 20 Hz”. This sentence is confusing, the authors should re-construct this sentence to remove ambiguity about light being delivered via electrode array.
6. The authors should specify which electrode were used for these recordings and how do they interface these electrodes with the subdiaphragmatic vagus?
7. More appropriate controls are necessary for optoelectrophysiology of the vagus [Fig. b]. It is well known that many electrode show optically induced artifacts. The authors should perform the following controls : (a) Green light stimulation and recording in ChAT-ChR2 mice; (b) Blue-light stimulation and recording in ChAT(-) littermate mice.
8. The authors should remove un-necessary use of superlatives/adjectives in the manuscript unless they are backed by comparative data from the literature: examples include “excellent biocompatibility”
9. In glucose homeostasis experiments how do the authors select 20Hz frequency? Have the authors tried other frequencies and decided to use 20Hz since it was producing highest effect? Is 20Hz stimulation of pancreatic vagus backed by literature references? What is the natural firing frequency of the pancreatic vagus to maintain glucose hemostasis?
10. Provide error bars and standard deviation for plots in Figure 5c and 5e. Specify number of mice used in these experiments.
11. Do the TAHOFs fail after 3 days? If yes specify the mode of failure.
12. The chronic validation of TAHOFs in the glucose homeostasis study is limited to only 3 days which significantly lowers the impact of this work. The broad motivation behind developing TAHOFs, according to authors, is to achieve stable optical interfacing with dynamical visceral organs. 3 days too short time to prove the utility of this technology. Most in-vivo biological studies in mice need at least 2 weeks of stable device performance to provide experimenters. If adding CGM module along with TAHOF is too much of an implant burden on the animal for 2 weeks, the authors can switch to blood draws to measure blood glucose for the chronic studies.
13. In the conclusion section the authors should refrain from making exaggerated claim about “therapeutic potential” of their technology. While these devices are very useful to decipher and understand fundamental neurobiology of autonomic and somatic peripheral neural circuits, their therapeutic value is not demonstrated in any meaningful way in this work.

Reviewer #2

(Remarks to the Author)
The article “Tissue-adhesive Hydrogel Optical Fiber for Peripheral Optogenetic Neuromodulation” reports a soft, biocompatible, and adhesive optical interface designed for stable light delivery to mechanically dynamic visceral organs. The device integrates a poly(HEMA) light-guiding core (ncore = 1.429) with a PAA-PVA bioadhesive cladding (ncladding = 1.342), yielding low propagation loss (0.529 dB/cm), robust interfacial adhesion (12 kPa), and high stretchability (176%). The authors further develop an integrated optogenetic platform and apply pulsed blue-light stimulation (470nm) to the pancreatic vagus nerve in freely moving ChAT-ChR2 mice, achieving real-time glycemic regulation and sustained blood glucose control over three days in diabetic models. Overall, the study demonstrates how the synergistic integration of optical confinement, mechanical compliance, and tissue adhesion can advance spatiotemporally precise neuromodulation for anatomically complex biological systems. However, several minor issues require clarification before the manuscript can be considered for publication in Nature Communications.

Comment #1: In the manuscript, the authors present several technical considerations relating to the use of 470 nm light, such as ChR2 activation, low propagation loss, and tissue-specific attenuation. However, these elements are currently scattered across the main text and supplementary materials, which weakens the clarity of the rationale behind optimising a blue-light system for deep-visceral applications where significant scattering occurs. To address this, the authors should compile these points into a consolidated 'design rationale' section that explicitly links the TAHOF architecture to the mitigation of blue-light-specific limitations.

Comment #2: In the manuscript, the authors emphasize the broad applicability of the TAHOF platform to diverse morphologically dynamic organs and chronic neuromodulation therapies beyond the pancreatic use case. However, the current biological demonstrations are limited to a single opsin (ChR2), a single wavelength band (~470 nm), and a single physiological axis (pancreatic nerve–glucose regulation). To further strengthen the generality of the platform claim, it would be valuable to include at least one additional proof-of-concept—such as stimulation using a different opsin class, operation at a red-shifted wavelength, or a second organ/readout model—or to explicitly clarify the current scope and limitations of the biological demonstrations.

Comment #3: The waveguiding performance of TAHOFs relies critically on refractive-index contrast between the core, cladding, and surrounding tissue. While the manuscript notes that biological tissues can span a relatively broad refractive-index range (RI = 1.33–1.51), the numerical analysis appears to adopt a single representative value for the surrounding tissue (ntissue = 1.500). It therefore remains unclear whether comparable confinement and leakage suppression are maintained across the full tissue refractive-index range encountered in vivo. A brief sensitivity analysis of waveguiding performance versus tissue refractive index, or a justification that the chosen tissue represents a conservative worst case, would strengthen the robustness and generality of the optical design.

Comment #4: While the study successfully demonstrates stable optical stimulation and glycemic regulation over 3 days, caution is warranted in extrapolating these findings to chronic or long-term applications. The current results are best interpreted as a short-term proof-of-concept; accordingly, the manuscript should either frame the outcomes explicitly as short-term achievements or extend the duration of the in vivo experiments to substantiate claims of longer-term applicability.

Comment #5: While the electrophysiological recordings in Fig. 4b demonstrate time-locked vagal nerve activity during optical stimulation, repeated light ON–OFF stimulation paradigms are needed to further establish a causal relationship between optical stimulation and neural activation.

Comment #6: Currently, quantitative lap-shear adhesion testing is limited to porcine skin, which represents a limitation given the emphasis on broad applicability in dynamic biological environments. To strengthen the generalizability of the TAHOF platform, simple lap-shear adhesion tests on multiple representative wet tissues should be included.

Comment #7: Please clarify the figure citation “f Supplementary Fig. 16c and 17,” on page 9, as the current notation is ambiguous.

Reviewer #3

(Remarks to the Author)
This work proposes a novel hydrogel-based optical fiber with enhanced tissue-adhesive properties for optogenetic neuromodulation in the peripheral nervous system. The concept is extremely relevant for the application of optogenetics in regions of the body where significant movement of organs and tissues (e.g. peristalsis) might cause a disconnection of the probe from the tissue, leading to a misplacement of the stimulated area. The methodology of the investigation is sound, and the conclusions are supported by extensive experimental validation which is also accurately described in the main text and supplementary information. Overall the work is novel, significant and well presented. Only a few minor comments should be, in my opinion, addressed:

1. In Figure 1 and Supplementary Figure 6, the word "porcine" is misspelled;

2. In the Introduction, in the paragraph following Figure 1, the authors refer to the cladding as "multifunctional". This term is typically used in the field for optical fibers that provide functions other than light delivery (e.g. drug delivery,

electrophysiology), which does not seem to be the case in this work. I therefore suggest rephrasing this sentence;

3. In the "Design and fabrication of TAHOFs" section, the discussion of the loss performance is made somewhat harder to read by the use of percentage values both for normalized transmission and transmission variation. As an example, the sentence "they maintained > 95% transmission integrity after 10,000 strain cycles" might be, at a first glance, interpreted as the fiber transmitting 95% of input light, which is inconsistent with the reported losses values. I suggest this part is rewritten in a clearer way, for example by providing absolute values of losses for the light propagation in tissue and clarifying the reference values for transmission variations;

5. In Supplementary Figure 6, the X-axis should be in linear scale;

6. In Figure 2g, the attenuation in the fiber appears to be actually decreasing with increasing numbers of stretch cycles, which appears to be contrary to what is written in the text;

7. In page 5, the authors write "[...] hydrogel fibers without adhesive cladding are prone to dislocate under dynamic loads, leading to off-target illumination (Fig. 2h)". However, Fig 2h does not show this, but rather that a TAHOF does not dislocate;

8. In the "Biomechanical integration and strain-resilient optical delivery" section, the authors mention wavelength-dependent attenuation profiles. However, all attenuation measurements seem to be performed at 470 nm, rather than showing wavelength dependencies;

9. In the "Neuromodulatory control of blood glucose via the vagus-pancreas axis enabled by TAHOFs" section, the authors have implanted an electrode array adjacent to the optical neuromodulation point to measure optically-induced LFPs. Have the authors considered or somehow mitigated the possibility of photoelectric artifacts affecting the LFP measurements? The paper would, in my opinion, benefit from a brief discussion of this issue.

Version 1:

Reviewer comments:

Reviewer #1

(Remarks to the Author)
The authors have addressed my major concerns. I have a few minor suggestions.

1. In the Introduction, the authors should clarify that the hydrogel chemistries are based on previously published literature, and that the primary contribution of this work lies in their application toward tool development.
2. Standard error or standard deviation should be reported for all experimentally measured and quantified parameters mentioned in the text. For example, values such as “low propagation loss (0.529 ± xx dB/cm)” should include the appropriate measure of variability.
3. When the device is implanted in mice, the ferrule will remain externalized. Over time, the hydrogel optical fiber threaded into the ferrule may dehydrate, which could lead to poorer coupling with the silica coupling fiber, increased optical loss, and possible mechanical failure. The authors should address this concern and discuss a potential mitigation strategy.
4. The authors use transgenic mice that express ChR2 in all ChAT+ neurons. It is important to note that ChAT vagal neurons innervate not only the pancreas, but also many other organs in the abdominal cavity, including the stomach, duodenum, ileum, and hepatic portal vein. This point is particularly relevant because the authors do not use a light-blocking cladding along the length of the fiber, which creates the possibility of light leakage into surrounding tissues. In several images, substantial light leakage along the fiber length is clearly visible. This could result in unintended optogenetic stimulation of other abdominal organs. The authors should address this issue and provide a mitigation strategy, either in the Discussion section or as a supplemental note in the Supporting Information.
5. In Fig. R5a, the title indicates “ChR2-Cre,” whereas the caption refers to “ChR2(-) littermates,” which would imply control mice that do not express ChR2 after breeding with the Ai27 line. Please ensure consistency.

Reviewer #2

(Remarks to the Author)
The authors have thoroughly addressed all previous comments by providing a consolidated design rationale for the blue-light system , expanding the biological validation to include gastric neuromodulation , and providing robust 14-day longitudinal data to support the chronic utility of the TAHOF platform. The comprehensive experimental and analytical improvements have brought the work to a high standard suitable for Nature Communications, and the manuscript is now recommended for publication in its current form.

Open Access This Peer Review File is licensed under a Creative Commons Attribution 4.0 International License, which permits use, sharing, adaptation, distribution and reproduction in any medium or format, as long as you give appropriate credit to the original author(s) and the source, provide a link to the Creative Commons license, and indicate if changes were made.
In cases where reviewers are anonymous, credit should be given to 'Anonymous Referee' and the source.
The images or other third party material in this Peer Review File are included in the article's Creative Commons license, unless indicated otherwise in a credit line to the material. If material is not included in the article's Creative Commons license and your intended use is not permitted by statutory regulation or exceeds the permitted use, you will need to obtain permission directly from the copyright holder.
To view a copy of this license, visit https://creativecommons.org/licenses/by/4.0/

RE: Our point-by-point response to the reviewers' comments (NCOMMS-26-003310). Response to Reviewer #1

Comment 1: While tissue adhesive light-delivery devices are an exciting concept, the novelty in TAHOFs presented in this work is rather low. There is abundant literature on hydrogel optical fibers as well as bio-adhesive hydrogel coatings. The authors have merged previously reported approaches in this work without introducing any new chemistry and/or materials approach.

Answer: We thank the reviewer for this important comment. We agree that hydrogel optical fibers and bioadhesive hydrogels are well established, with numerous high-quality examples reported (e.g., *Nature* 575, 169174 (2019); *Nat. Methods* 20, 1802-1809 (2023)). We emphasize, however, that the contribution of this work is **not intended as a new chemistry or material system, but rather as a previously unaddressed device-level solution to a specific and critical application constraint**.

Recent advances in peripheral optogenetics have enabled control over metabolic, immune, and secretory functions (e.g., *Science* 381, 1480-1487 (2023); *Nat. Biotechnol.* 42, 892-904 (2024); *Nat. Biomed. Eng.* 8, 1501 (2024)). Despite this progress, most studies remain confined to anesthetized settings, in large part because **stable, localized light delivery cannot be maintained in freely moving systems**. Rigid silica fibers fail under physiological motion, while hydrogel optical fibers, although mechanically compliant, **do not inherently prevent displacement-induced off-target illumination**, which directly compromises neuromodulation precision. We further note that existing fixation strategies, including hydrogel cuff-like structures (e.g., *Nat. Methods* 20, 18021809 (2023); *Nat. Commun.* 15, 3525 (2024)), are fundamentally limited to **geometrically regular and mechanically stable targets** such as peripheral nerves. In contrast, visceral organs (e.g., pancreas, gastrointestinal tract) are **irregular, highly deformable, and continuously moving**, creating a qualitatively different interface problem that current designs do not resolve. The lack of a strategy for maintaining *on-target optical coupling* under these conditions represents a clear and unmet need in the field.

Within this context, TAHOF introduces a **distinct architectural principle**, rather than a simple combination of prior elements. Specifically, it integrates **tissue adhesion and optical confinement into a single cladding layer**, enabling simultaneous mechanical fixation and light guidance within a unified fiber geometry. This coupling is non-trivial and functionally essential: by co-localizing adhesion and refractive index contrast, the design directly addresses the root cause of signal loss, relative motion at the tissue-device interface, thereby enabling **stable, on-target illumination in dynamic environments**.

The significance of this architecture is supported by both in vitro and in vivo results. Compared to nonadhesive HOFs, TAHOFs exhibit markedly reduced displacement and off-target illumination under deformation, while MRI tracking confirms stable adhesion on the dynamically moving pancreatic surface (**revised Fig. 3g**). This capability enables peripheral optogenetic neuromodulation in freely moving animals, yielding measurable physiological outcomes, including enhanced insulin secretion and improved glycemic control. We have further strengthened this

point with 14-day post-implantation validation in the revised manuscript (**revised Fig. 5f**). In response to the reviewer's comment, we have revised the text to more precisely frame this work as an **application-**

driven, architecture-level innovation, and moderated any statements that could be interpreted as claims of new material chemistry. The corresponding revisions are highlighted in yellow.

Comment 2: How are the TAHOFs optically coupled to Silica fiber for light delivery? This is a high mechanical mismatch interface which can be prone to failure. The authors should describe in detail about this junction as well as its mechanical stability.

Answer: We thank the reviewer for this important comment. We would like to clarify that the TAHOF is **not directly coupled to a silica fiber**, and therefore avoids the mechanically fragile silica-hydrogel interface highlighted by the reviewer. Instead, optical coupling is achieved via a custom ceramic ferrule, which serves as a mechanically stable intermediary between the light source and the hydrogel fiber (**Fig. R1a**).

Specifically, in the dry state, the TAHOF has a diameter of 528 ± 16 μm and can be readily inserted into a ceramic ferrule with an inner diameter of 650 μm. Upon immersion in PBS for 4 h, the hydrogel fiber swells to 666 ± 14 μm (**Fig. R1b-d**), reaching equilibrium and forming a **conformal, interference fit** within the ferrule. This swelling-assisted coupling eliminates interfacial gaps and enables stable optical transmission without requiring rigid bonding or adhesives. Similarly, the swollen PHEMA core (558 ± 11 μm) is well matched to a ferrule with an inner diameter of 550 μm, further supporting stable alignment and light injection.

This design is critical from a mechanical standpoint: by **decoupling the compliant hydrogel from the rigid silica fiber** and instead interfacing both through the ceramic ferrule, the system mitigates stress concentration and reduces the risk of interfacial failure under handling or operation. The resulting junction is mechanically robust under routine manipulation and maintains consistent optical coupling during use.

To address the reviewer's concern, we have now added a detailed description of the TAHOF–ferrule junction and the laser coupling configuration in the revised ***Methods section*** ("*Coupling of TAHOF to a custom ceramic ferrule for laser connection*") and **Supplementary Fig. 3**, as highlighted in yellow.

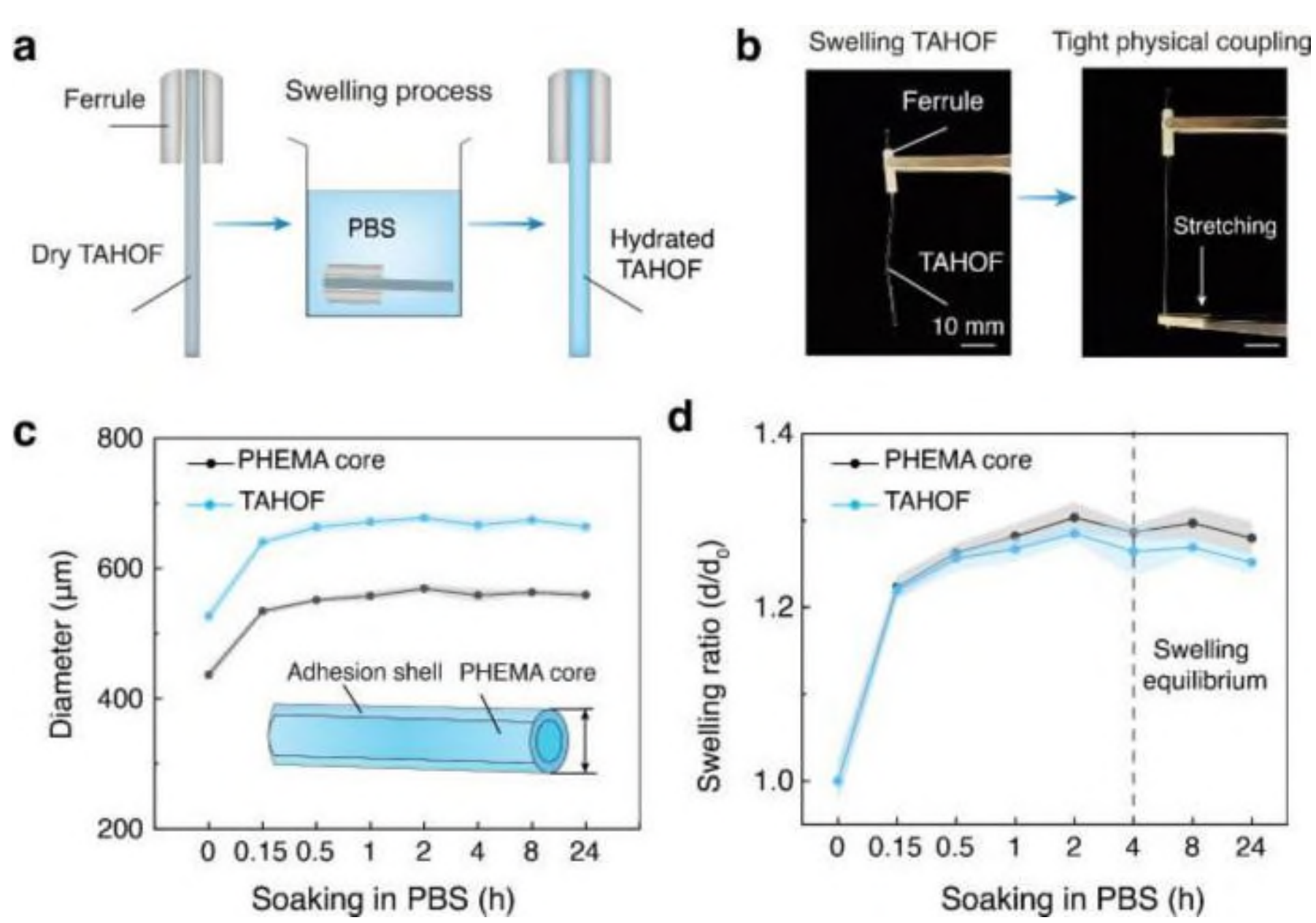

Fig. R1 Swelling-assisted coupling of the TAHOF with a ceramic ferrule for optical connection. a, Schematic illustration of the TAHOF-ceramic ferrule junction formed by swelling-assisted fitting. **b,** Photograph of the tightly coupled TAHOF-ceramic ferrule interface after the hydrogel fiber reached swelling equilibrium. Scale bar, 10 mm. **c,d,** Changes in the diameter (**b**) and swelling ratio (**c**) of the TAHOF and PHEMA core during immersion in PBS for 24 h. The data at 0 h correspond to the dry state. Data in (**c**) and (**d**) are presented as mean ± SD (n = 5).

Comment 3: Experimental in-vivo validation of tissue adhesion of TAHOFs and control optical fibers is not convincing. The authors demonstrate the adhesiveness of TAHOFs in an acutely implanted animal by mechanically pulling on the light-coupling fiber [Fig. 3f-k]. This scenario is rather unusual, exaggerated, and very unlikely to occur in a freely moving animal. To convincingly prove the adhesive nature of TAHOFs compared to controls in a native physiological context, the authors should chronically implant these fibers (without silica coupling) for 2 weeks and then perform CT imaging on these mice to locate the fibers. The authors can add a CT contrast agent in their hydrogels to help visualize the fibers under CT. It is important to convincingly prove the readers that large displacement indeed takes place in control hydrogel optical fibers.

Answer: We thank the reviewer for this comment. We thank the reviewer for this important and constructive comment. We agree that **chronic in vivo validation under native physiological conditions is essential** to rigorously establish the adhesive advantage of TAHOFs over control fibers. Following this suggestion, we have performed an additional **14-day chronic implantation study in freely moving animals**, with longitudinal tracking of fiber position at day 1, day 3, and day 14.

While the reviewer suggested CT imaging, we did not adopt this approach because hydrogel optical fibers are highly hydrated materials with intrinsically low X-ray attenuation, making them difficult to visualize without exogenous contrast agents. Importantly, incorporating such agents would require modification of the hydrogel composition and could alter its mechanical and adhesive properties, thereby confounding the interpretation. Instead, we employed **high-field (9.4 T) MRI**, which is well suited for visualizing soft, water-rich implants without altering the original material system (*Sci. Rep.* 8, 16490 (2018)).

As shown in **Fig. R2**, MRI enables clear and longitudinal visualization of both TAHOFs and control hydrogel optical fibers (HOFs). At day 1, both fibers are correctly positioned at the pancreatic target site. However, by day 3, a pronounced divergence emerges: the TAHOF remains stably anchored in close contact with the pancreas, whereas the control HOF exhibits substantial displacement, migrating away from the target region toward the subcutaneous space. This displacement becomes even more pronounced by day 14, with the control fiber further shifting along the kidney region. In contrast, the TAHOF consistently maintains **stable, on-target positioning throughout the entire observation period**, despite continuous physiological motion of the pancreas and surrounding tissues (**Fig. R2b**). Importantly, ex vivo anatomical validation at day 14 (**Fig. R2c**) directly confirms persistent adhesion of the TAHOF to the pancreatic surface.

These results provide **direct and physiologically relevant evidence** that significant displacement indeed occurs in non-adhesive hydrogel optical fibers under native conditions, and that this limitation is effectively mitigated by the adhesive cladding design of TAHOFs. In response to the reviewer's concern regarding the acute pulling experiment, we have repositioned this test to the Supplementary Information (**Supplementary Fig. 17**) as

a **stress-test scenario**, while elevating the chronic MRI study as the primary validation (**Fig. 3f,g** and **Supplementary Fig. 18**). All corresponding revisions have been highlighted in yellow.

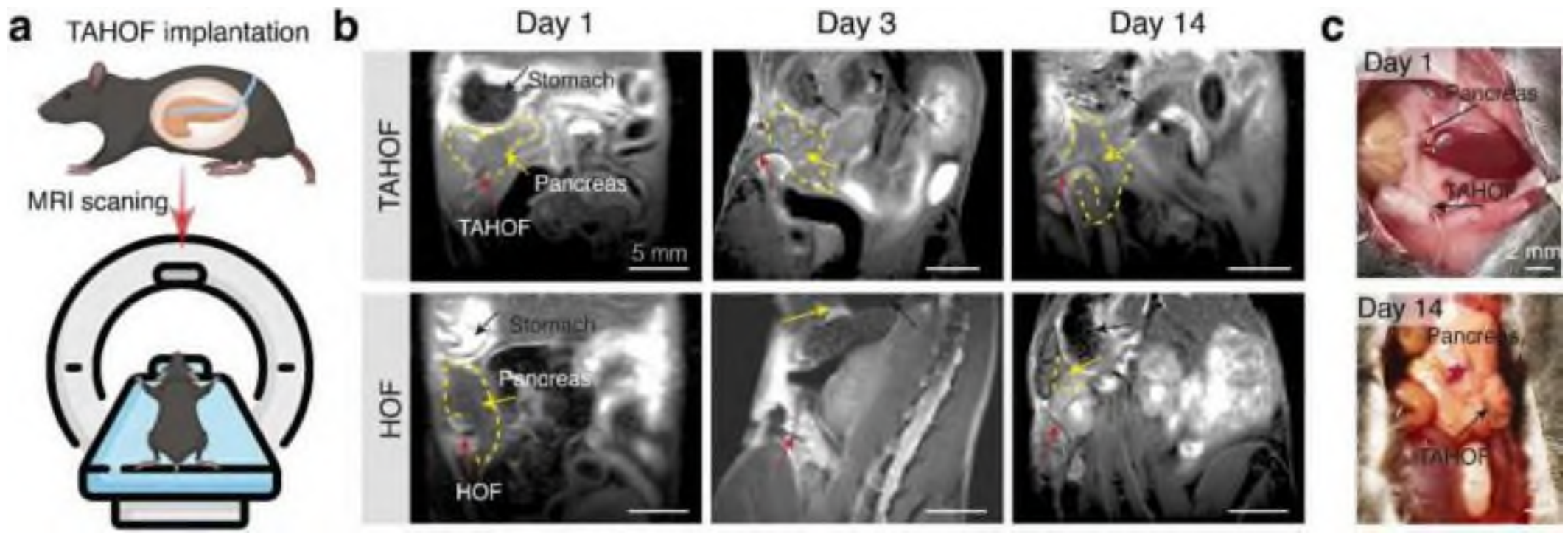

Fig. R2 In vivo validation of tissue adhesion of TAHOF after implantation on the pancreas. a, Schematic illustration of TAHOF implantation on the mouse pancreas and longitudinal tracking of the implanted fiber by MRI. **b,** Representative MRI images of implanted TAHOFs at day 1, day 3, and day 14. Red arrows indicate the TAHOF, yellow arrows indicate the pancreas, yellow dashed lines outline the pancreatic region, and black arrows indicate the stomach. Implantation of HOF served as the control. **c,** Photographs of TAHOF implantation on the pancreas at day 1 and day 14, showing maintained adhesion after implantation.

Comment 4: Validation of ChR2 expression in pancreatic vagal afferents is lacking.

Answer: We thank the reviewer for this valuable comment regarding the ChR2 expression in pancreatic vagal fibers. In this study, we used offspring generated by crossing ChAT-Cre mice with Ai27 reporter mice, in which ChR2 is selectively expressed in cholinergic neurons. In these mice, cholinergic vagal fibers innervating the pancreas are labeled with tdTomato, enabling direct visualization of their distribution. **Fig. R3** showed the tdTomato fluorescence was observed in vagal ChAT-positive fibers within the pancreas, confirming the expression of ChR2 in these projections.

Following the reviewer's suggestion, we have further included additional immunostaining data in our revised manuscript (**Supplementary Fig. 19**) and expanded the corresponding description and discussion in the manuscript to clarify this point.

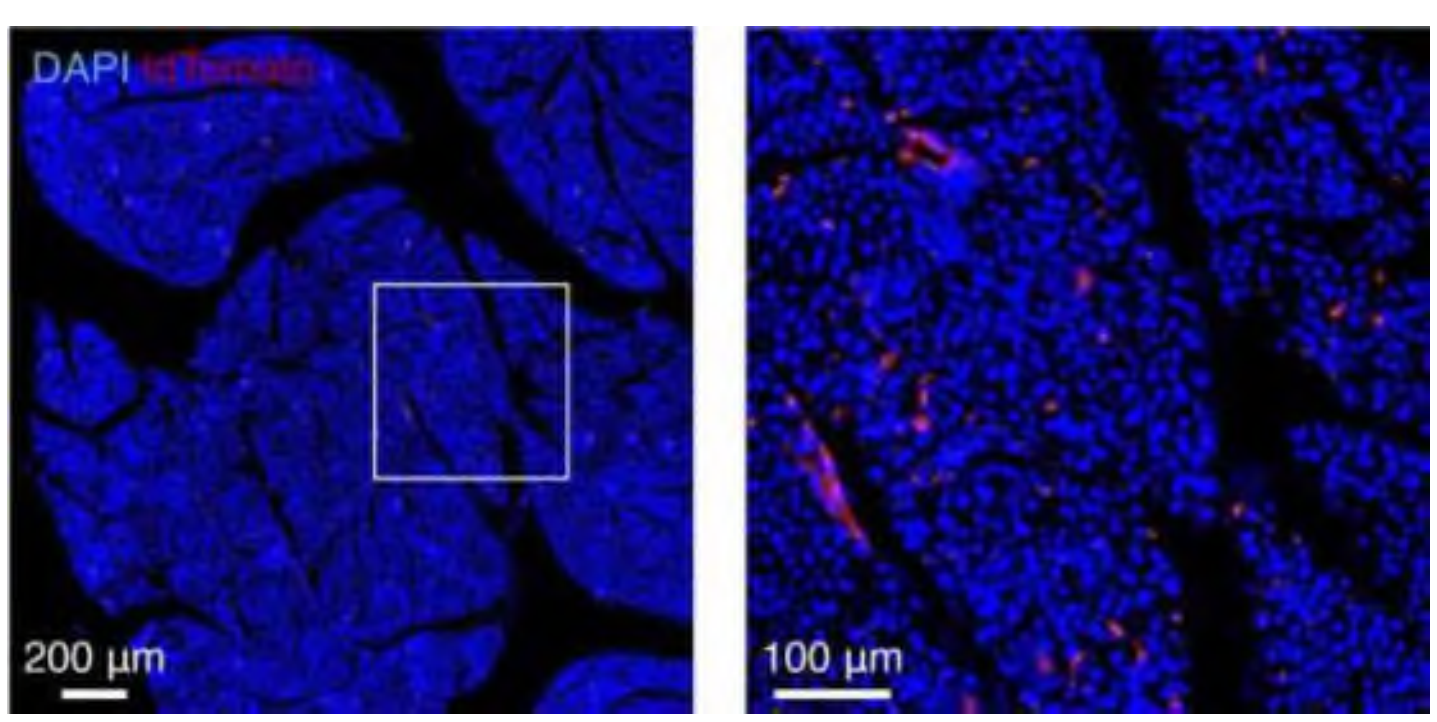

Fig.R3 ChR2-tdTomato was expressed in pancreatic vagal fibers of ChAT-ChR2 mice.

Comment 5: "The implanted TAHOF delivered blue light pulses (473 nm, 20 mW, 20 Hz, 5 ms) to the subdiaphragmatic vagus nerve via an adjacent electrode array, inducing robust, time locked local field potentials (LFPs) at 20 Hz". This sentence is confusing, the authors should re-construct this sentence to remove ambiguity about light being delivered via electrode array.

Answer: We thank the reviewer for pointing out this ambiguity. We agree that the original wording could be misinterpreted as implying that light was delivered via the electrode array. In fact, light delivery was achieved through the implanted TAHOF, while the adjacent cuff electrode array was used solely for LFP recording. To clarify this point, we have revised the sentence as follows: "*Blue light pulses (470 nm, 20 Hz, 5 ms) were delivered to the subdiaphragmatic vagus nerve via the implanted TAHOF, while local field potentials (LFPs) were recorded using an adjacent cuff electrode array, showing robust time-locked responses at 20 Hz.*" This has been corrected in the revised manuscript (**Fig. 4b-c**), as highlighted in yellow.

Comment 6: The authors should specify which electrode were used for these recordings and how do they interface these electrodes with the subdiaphragmatic vagus?

Answer: We thank the reviewer for this helpful suggestion. In the revised manuscript, we have added a detailed description of the electrode configuration and its interface with the subdiaphragmatic vagus in the Methods section. Specifically, **a cuff electrode (inner diameter: 0.2 mm, edge length: 0.5 mm, inter-electrode spacing: 1 mm)** was gently wrapped around the isolated subdiaphragmatic vagal trunk after separation from the subdiaphragmatic oesophagus, serving as the recording electrode (**Fig. R4**). For optical stimulation, the TAHOF was positioned adjacent to the vagal trunk and adhered to the oesophagus and surrounding tissue to maintain light delivery to the target nerve. Thus, the cuff electrode was used for electrophysiological recording, whereas the TAHOF served as the optical stimulation interface.

Following the reviewer's suggestion, these experimental details have been incorporated into the revised Methods section ("*Optogenetic stimulation and electrophysiological recording*"), and representative images of the stimulation/recording configuration have been added (revised **Fig. 4b**). All corresponding revisions are highlighted in yellow.

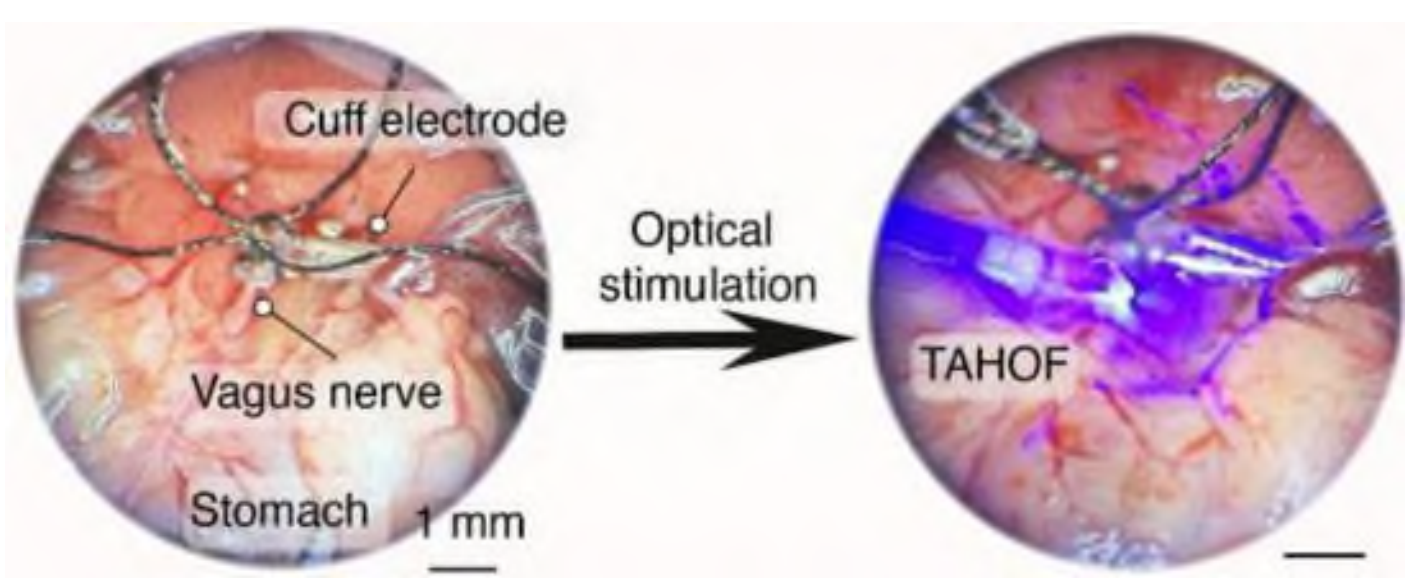

Fig. R4 Photograph of the experimental setup for optical stimulation of the dissected subdiaphragmatic vagus nerve in ChAT-ChR2 mice. The image shows electrophysiological recording using a cuff electrode and optical stimulation delivered by the TAHOF. Scale bar, 1 mm.

Comment 7: More appropriate controls are necessary for optoelectrophysiology of the vagus [Fig. 4b]. It is well known that many electrodes show optically induced artifacts. The authors should perform the following controls: (a) Green light stimulation and recording in ChAT-ChR2 mice; (b) Blue-light stimulation and recording in ChAT(-) littermate mice.

Answer: We thank the reviewer for this important suggestion. To address the concern regarding potential optically induced recording artifacts, we performed two additional control experiments under the same stimulation and recording configuration. First, we applied **470 nm blue-light stimulation in ChR2(-)** littermate mice implanted with the TAHOF and recording cuff electrode. Second, we applied **589 nm yellow-light stimulation in ChAT-ChR2 mice** as a wavelength control. In both conditions, LFP spectrogram analysis showed no discernible increase in spectral power around the stimulation frequency (**Fig. R5**). In contrast, clear time-locked LFP responses were observed only during blue-light stimulation in ChAT-ChR2 mice (**revised Fig. 4c**).

In addition, the TAHOF tip was positioned approximately 1-2 mm away from the recording cuff electrode to minimize direct illumination of the electrode (**revised Fig. 4b**). Together, these results indicate that the recorded responses arise from ChR2-dependent vagal activation rather than optical artifacts. Following the reviewer's suggestion, we have incorporated these control data (see new **Supplementary Fig. 20**) and the related discussion into the revised manuscript, as highlighted in yellow.

Fig. R5 Spectrograms of local field potentials (LFPs) recorded from the subdiaphragmatic vagus nerve during

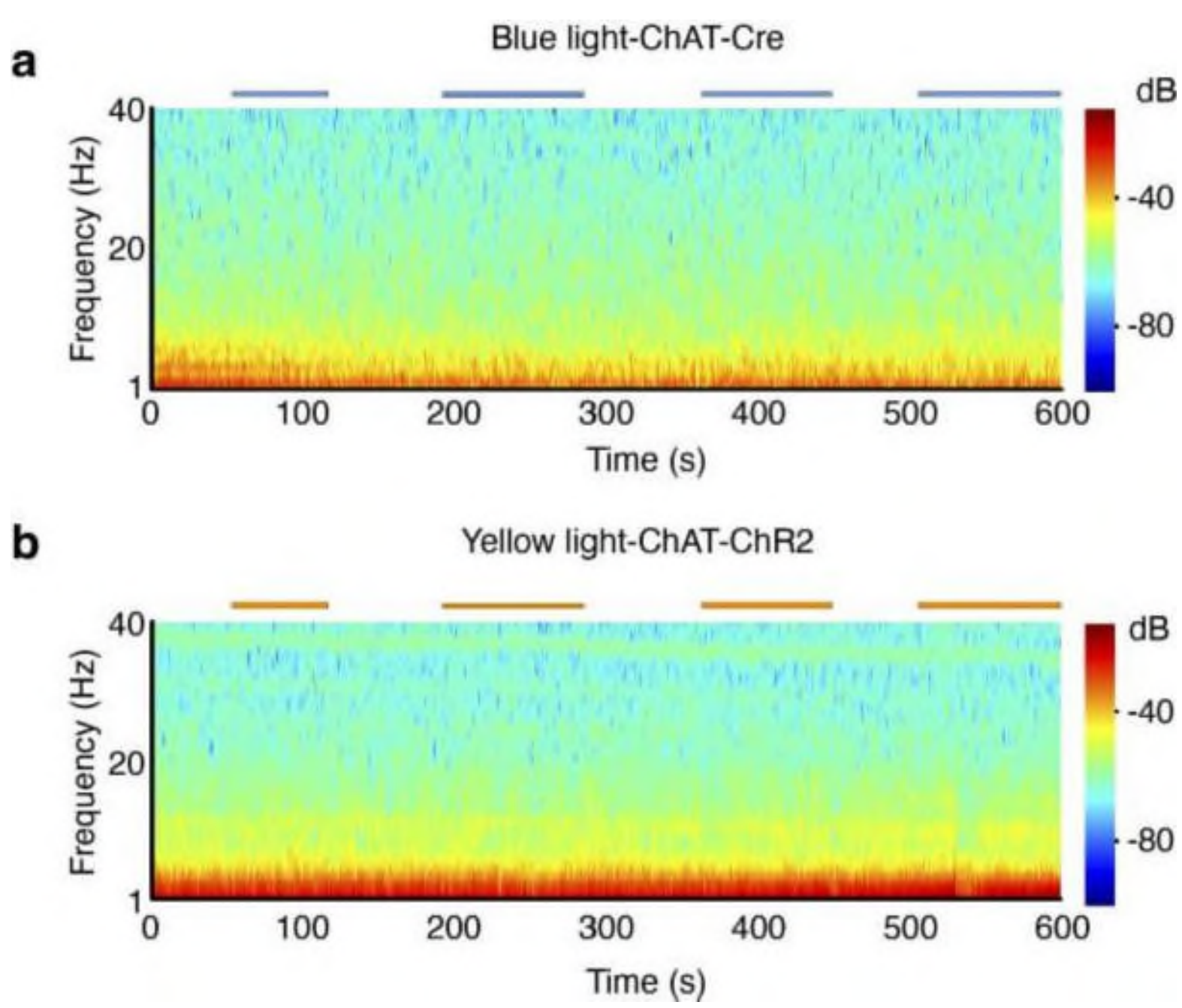

optical stimulation. a, Spectrogram of LFPs recorded with a cuff electrode in ChR2(-) littermate mice during pulsed blue-light stimulation (470 nm, 20 Hz, 5 ms) delivered through the TAHOF. **b,** Spectrogram of LFPs recorded with a cuff electrode in ChAT-ChR2 mice during pulsed yellow-light stimulation (589 nm, 20 Hz, 5 ms) delivered through the TAHOF.

Comment 8: The authors should remove un-necessary use of superlatives/adjectives in the manuscript unless they are backed by comparative data from the literature: examples include “excellent biocompatibility”

Answer: Thank you for this comment. We agree that superlative or strongly evaluative wording should be avoided unless it is directly supported by comparative data. Following this comment, we have carefully revised the manuscript throughout to remove or soften unnecessary superlatives and subjective adjectives. For example, “*ultra-low light loss*” has been revised to “*low light loss*”, “*exceptional strain tolerance*” to “*high strain tolerance*”, “*excellent biocompatibility*” to “*good biocompatibility*”. These revisions have been made throughout the revised manuscript and are highlighted in yellow.

Comment 9: In glucose homeostasis experiments how do the authors select 20Hz frequency? Have the authors tried other frequencies and decided to use 20 Hz since it was producing highest effect? Is 20Hz stimulation of pancreatic vagus backed by literature references? What is the natural firing frequency of the pancreatic vagus to maintain glucose hemostasis?

Answer: We thank the reviewer for this insightful comment regarding stimulation frequency. We first note that **20 Hz stimulation is a commonly used parameter in vagus nerve stimulation studies** and has been widely adopted in prior work investigating cholinergic and vagal modulation (*Nat. Biomed. Eng.* 8, 808-822 (**2024**), *Sci. Rep.* 11, 3670 (**2021**) and *Neuron* 110, 2867-2885 (**2022**)).

To further examine the effect of stimulation frequency in our glucose homeostasis experiments, we additionally tested 10 Hz, 20 Hz, and 30 Hz optical stimulation conditions in ChAT-ChR2 mice under otherwise identical parameters (5 ms, 20 mW) (**Fig. R6**). All tested frequencies were able to reduce blood glucose levels following intraperitoneal glucose injection, indicating that vagal activation itself is sufficient to induce glucose-lowering effects. Among them, 20 Hz produced a stronger reduction compared to 10 Hz, whereas increasing the frequency to 30 Hz did not result in a further significant improvement under our experimental conditions. Based on these results, we selected 20 Hz for subsequent experiments as a representative and effective stimulation frequency, rather than implying that it is uniquely required for eliciting the observed effects.

Regarding the physiological firing properties of pancreatic vagal fibers, their endogenous activity is generally sparse and occurs at low frequencies (*Am. J. Physiol. Gastrointest. Liver. Physiol.* 304, 437-448 (2012)). Therefore, we do not intend to suggest that 20 Hz reflects a natural firing frequency, but rather use it as an experimental parameter to reliably activate vagal cholinergic fibers. We have incorporated these results and the related discussion into the revised manuscript **Supplementary Fig. 27** and corresponding text), as highlighted in yellow.

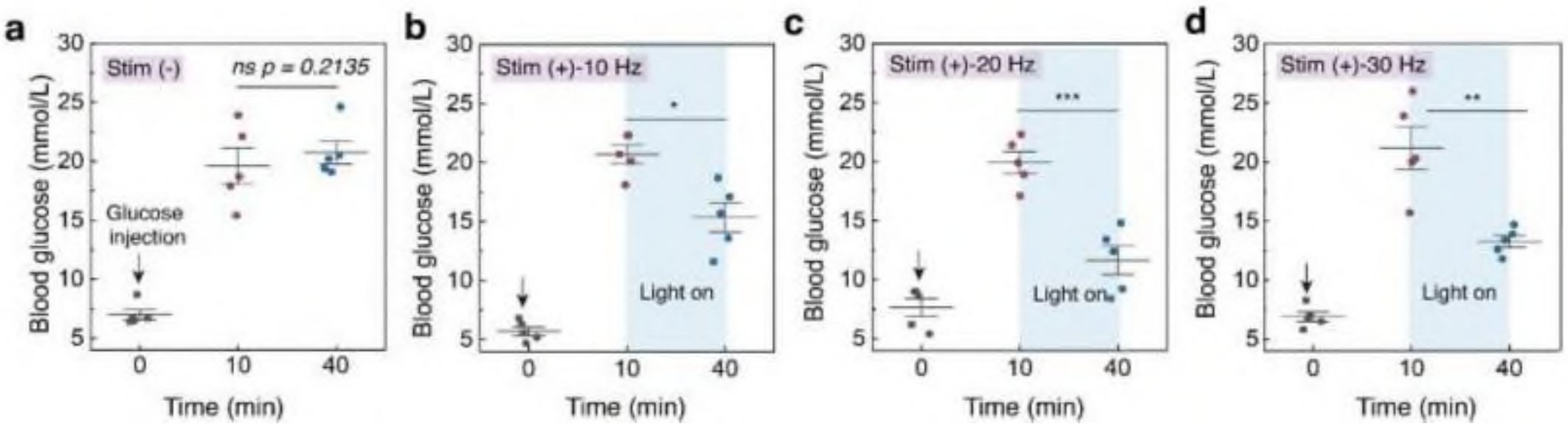

Fig. R6 Optogenetic stimulation via TAHOF during glucose homeostasis experiments in ChAT-ChR2 mice. **a**, Blood glucose levels measured for 40 min following intraperitoneal glucose injection (2 g/kg). **b-d,** Blood glucose levels measured during optogenetic stimulation of vagal fibers innervating the pancreas via TAHOFs (470 nm, 5 ms) at 10 Hz (**b**), 20 Hz (**c**), and 30 Hz (**d**). Intraperitoneal glucose injection (2 g/kg) was performed at 0 min, and optical stimulation was applied from 10 to 40 min. Blood glucose measurements were obtained from 5 mice. Statistical significance and p-values were determined using two-sided paired t-test. ns, not significant; *p < 0.05, ** p < 0.01, *** p < 0.001.

Comment 10: Provide error bars and standard deviation for plots in Figure 5c and 5e. Specify number of mice used in these experiments.

Answer: We thank the reviewer for this comment. As shown in **Figs. R7 and R8**, we have now added error bars to the plots for both the cyclic optical stimulation experiment and the sustained glycemic regulation experiment. The corresponding revisions have been incorporated into the manuscript in revised **Fig. 5c,** revised **Fig. 5e,** and new **Supplementary Figs. 29 and 30,** and these data are now presented as mean ± standard error (SE). We have also specified the number of animals used in these experiments in the revised figure legends (n = 4 mice per group). These changes have been incorporated into the revised manuscript and are highlighted in yellow.

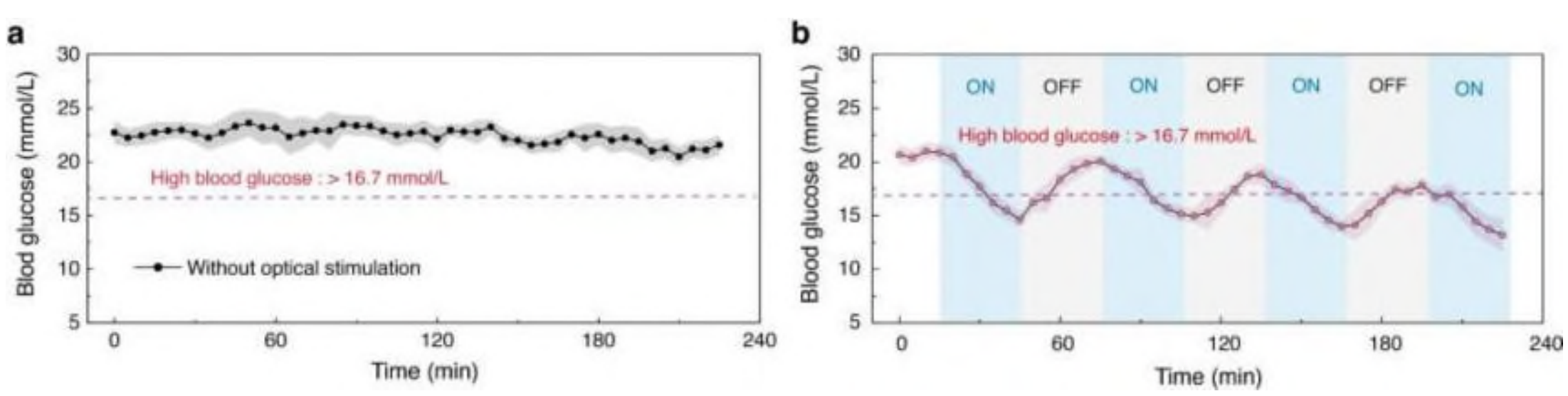

Fig. R7 Blood glucose monitoring in diabetic mice during cyclic optical stimulation via implanted TAHOF. a, Blood glucose levels continuously recorded over 240 min using an implanted continuous glucose monitoring system (CGM, SIBIONICS®) in ChAT-ChR2 diabetic mice implanted with TAHOFs but without optical stimulation. **b,** Blood glucose levels continuously recorded over 225 min using CGM in ChAT-ChR2 diabetic mice implanted with TAHOFs during cyclic optical stimulation (470 nm, 20 Hz, 5 ms) delivered through the fiber. n = 4 mice per group. Data are presented as mean ± SE.

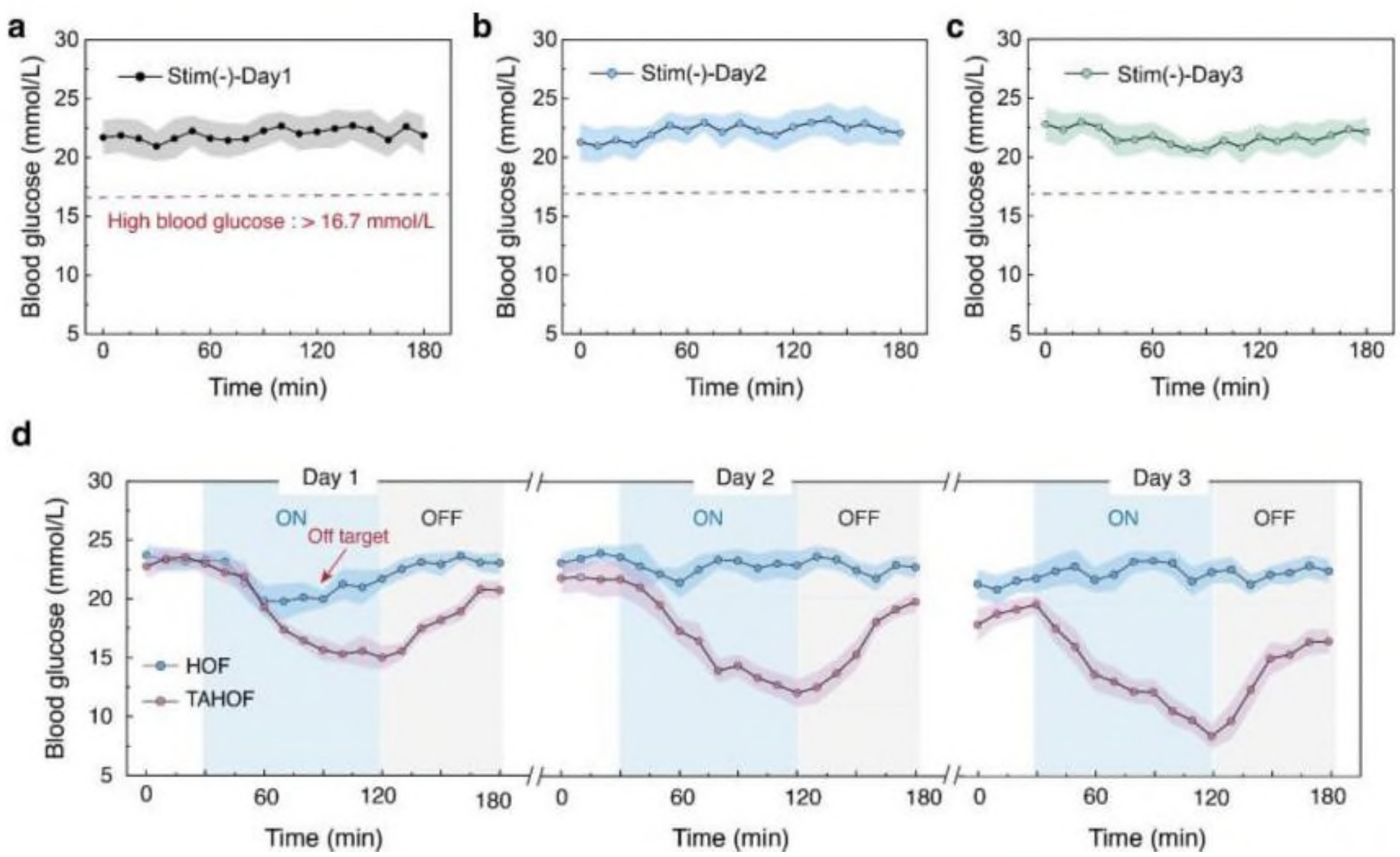

Fig. R8 Short-term glycemic modulation via optogenetic stimulation using TAHOFs in diabetic mice. a-c, Continuous blood glucose monitoring over 180 min per day on 3 consecutive days using a CGM system in ChAT-ChR2 diabetic mice implanted with TAHOFs but without optical stimulation. **d,** Sustained glycemic regulation over 3 days with daily 90-min optical stimulation (470 nm, 20 Hz, 5 ms) delivered via TAHOF, compared with fibers lacking adhesive cladding (HOF). n = 4 mice per group. Data are presented as mean ± SE.

Comment 11: Do the TAHOFs fail after 3 days? If yes specify the mode of failure.

Answer: We thank the reviewer for this comment. Under our experimental conditions, we **did not observe functional failure of the TAHOFs after 3 days, nor within the 14-day observation period**. In this study, we define functional failure based on two criteria: **(1) displacement from the target site and (2) loss of optical transmission sufficient to impair optogenetic stimulation**.

With respect to positional stability, longitudinal MRI tracking demonstrates that TAHOFs remain firmly adhered along the implantation path and maintain stable positioning on the pancreatic tissue for up to 14 days (**Fig. R2 and revised Fig. 3f-g**), indicating **no failure in adhesive function** over this period. In contrast, control hydrogel optical fibers exhibit pronounced displacement under the same conditions, further highlighting the robustness of the TAHOF interface. To evaluate implantation-associated changes in optical performance, we implanted TAHOFs (3 cm length) subcutaneously and retrieved them at 3, 7, 10, and 14 days for ex vivo transmission measurements. As shown in **Fig. R9**, the relative light transmission decreased to 79.1% ± 7.1% at day 3 and gradually declined to 58% ± 14% by day 14 (baseline = 100%). These results indicate a **progressive attenuation rather than abrupt device failure**. Importantly, the remaining optical output remained sufficient to elicit functional optogenetic responses (**Fig. R8 and revised Fig. 5e**), demonstrating that the device retains operational capability over this timeframe.

We attribute this gradual optical degradation primarily to **biofouling at the fiber interface**, including

adsorption of proteins and lipids (RI: 1.4-1.5), which can perturb the refractive index contrast of the core–cladding structure and increase light leakage (e.g., *Science* 385, eadm6869 (2024); *Nat. Protoc.* 21, 319–346 (2026)). Notably, this represents a **performance decay mechanism**, rather than a structural or mechanical failure of the device.

We have clarified this point in the revised manuscript (**Fig. 3h**) and added the corresponding data and discussion, as highlighted in yellow.

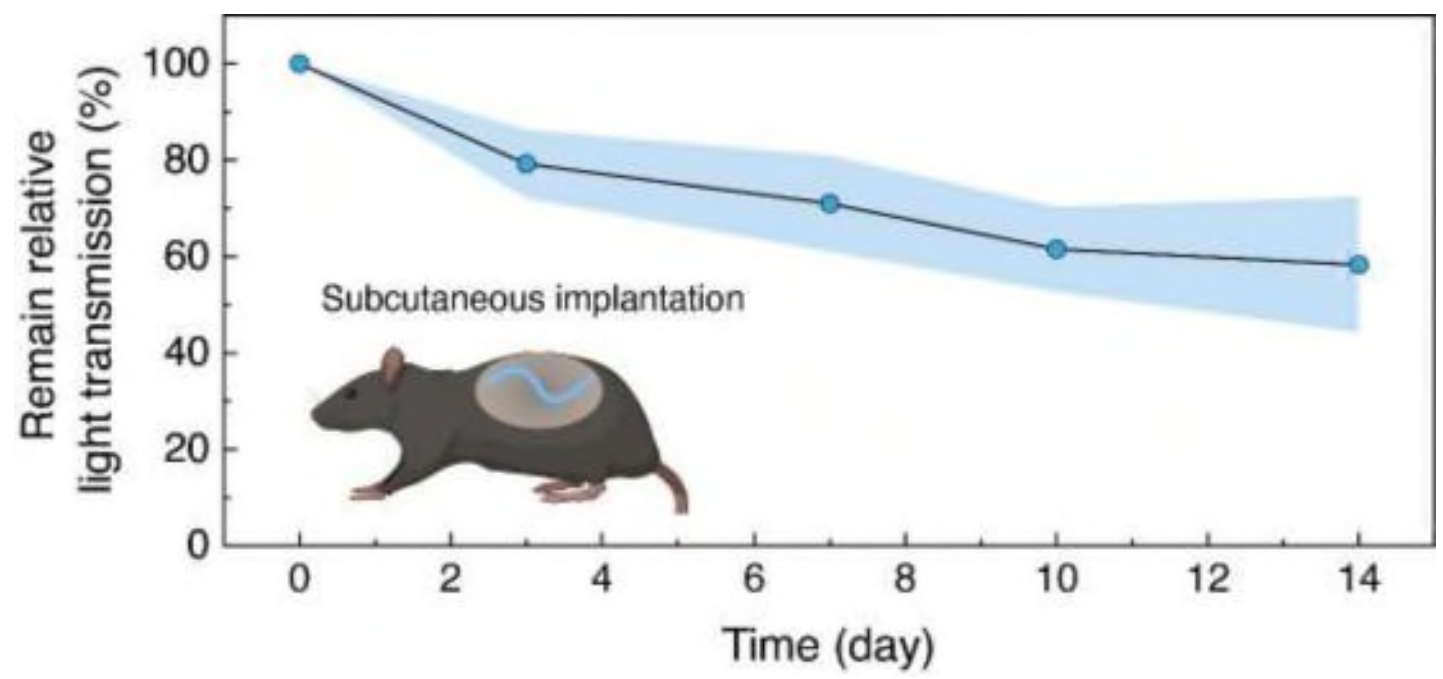

Fig. R9 Light transmission changes of TAHOF during in vivo implantation. Relative light transmission of TAHOFs after retrieval from the subcutaneous tissue on the mouse back at day 3, 7, 10, and 14. Output optical power was measured ex vivo and normalized to the pre-implantation level (day 0). Data are presented as mean ± SD, $n = 5$ samples.

Comment 12: The chronic validation of TAHOFs in the glucose homeostasis study is limited to only 3 days which significantly lowers the impact of this work. The broad motivation behind developing TAHOFs, according to authors, is to achieve stable optical interfacing with dynamical visceral organs. 3 days too short time to prove the utility of this technology. Most in-vivo biological studies in mice need at least 2 weeks of stable device performance to provide experimenters. If adding CGM module along with TAHOF is too much of an implant burden on the animal for 2 weeks, the authors can switch to blood draws to measure blood glucose for the chronic studies.

Answer: We thank the reviewer for this comment, and agree that the original 3-day study primarily demonstrates short-term functionality and is insufficient to establish the broader utility of TAHOFs for stable interfacing with dynamically moving visceral organs. Following this suggestion, we have performed an additional **14-day chronic in vivo validation study** to directly address this limitation. To minimize implant burden, we replaced the CGM module with tail-vein blood sampling for longitudinal glucose measurements, while maintaining optical stimulation via TAHOFs (**Fig. R10a,b**).

Across all examined time points (post-implantation days 1, 3, 7, 10, and 14), TAHOF-mediated optical stimulation (60 min per test) consistently induced **significant reductions in blood glucose levels** (**Fig. R10c-g and Fig. R11**). In each case, glucose levels decreased during stimulation and recovered after cessation, confirming that the effect is directly associated with optical neuromodulation. Quantitatively, blood glucose decreased by approximately 6-9 mmol L^{-1} at early time points (days 1-7, $^{**}p < 0.01$) and remained significantly reduced by ~4-5 mmol L^{-1} at later time points (days 10 and 14, $^{**}p < 0.01$). Although a gradual attenuation in response

magnitude is observed, the effect remains robust and statistically significant throughout the 14-day period.

Importantly, this attenuation is consistent with the progressive decrease in optical transmission (**Fig. R9**), rather than indicating device failure or loss of interface stability. Together, these results provide **direct functional evidence of sustained optogenetic neuromodulation over at least 14 days in vivo**, addressing the reviewer's concern regarding chronic performance. Accordingly, we have revised the manuscript to reposition the original 3-day study as short-term validation and to incorporate the new 14-day data and discussion (**Fig. 5f and Supplementary Fig. 33**), as highlighted in yellow.

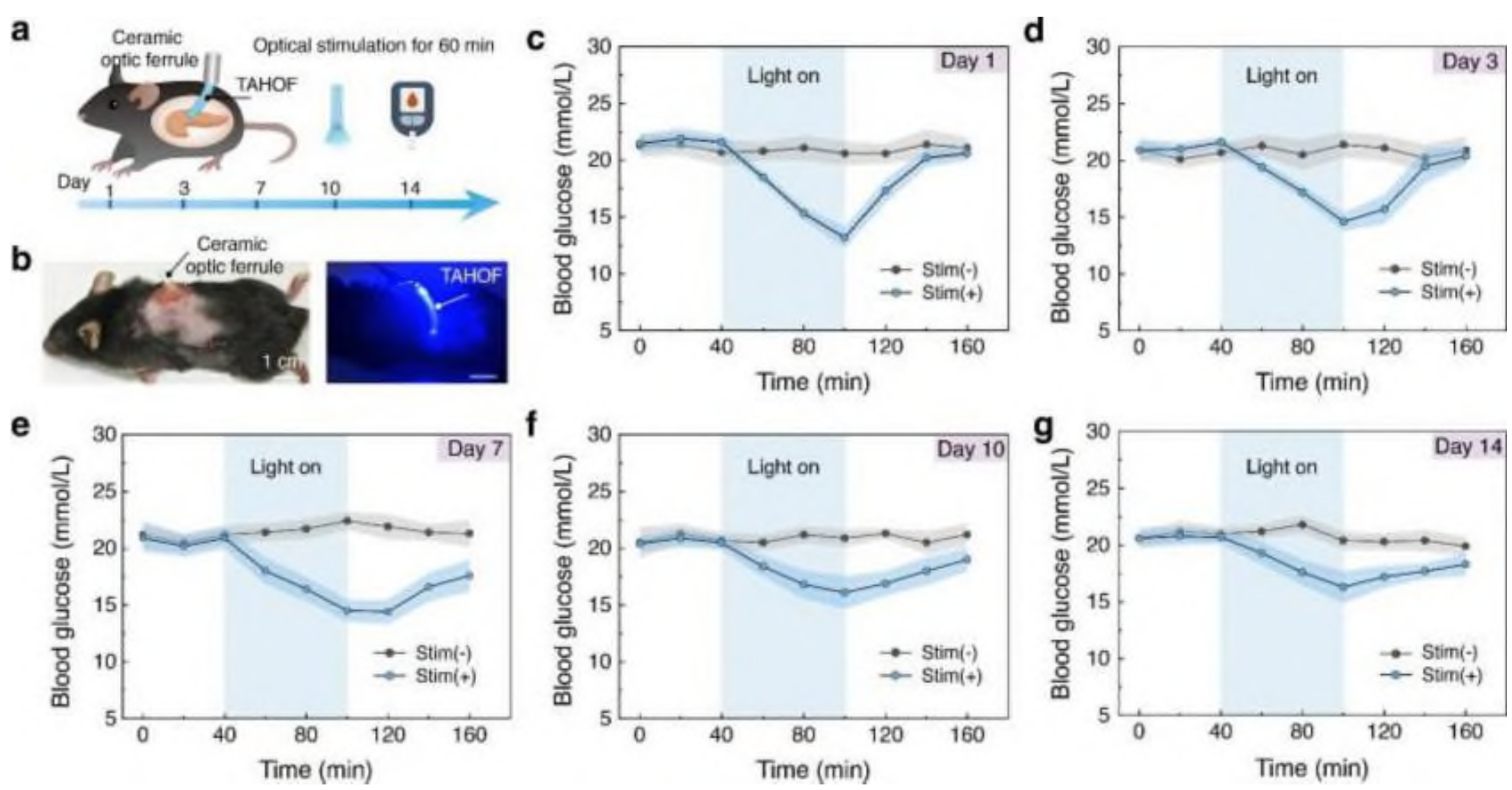

Fig. R10 Chronic optogenetic stimulation via implanted TAHOF in diabetic ChAT-ChR2 mice. a, Schematic of pancreatic TAHOF implantation and the chronic stimulation protocol. Optical stimulation was applied for 60 min at post-implantation day 1, 3, 7, 10, and 14. Blood glucose was measured every 20 min by tail-vein sampling using glucose test strips. **b,** Photograph of a TAHOF-implanted mouse. The fiber was fixed along the subcutaneous implantation path by the adhesive cladding, and the proximal end was coupled to a ceramic ferrule secured to the dorsal side of the mouse with dental cement. Scale bar, 1 cm. **c-g,** Blood glucose levels during 60-min optogenetic stimulation (470 nm, 20 Hz, 5 ms) via TAHOF at post-implantation day 1 (**c**), day 3 (**d**), day 7 (**e**), day 10 (**f**), and day 14 (**g**). Mice implanted with TAHOF but without optical stimulation served as controls. Data are presented as mean ± SE, with 5 mice per group.

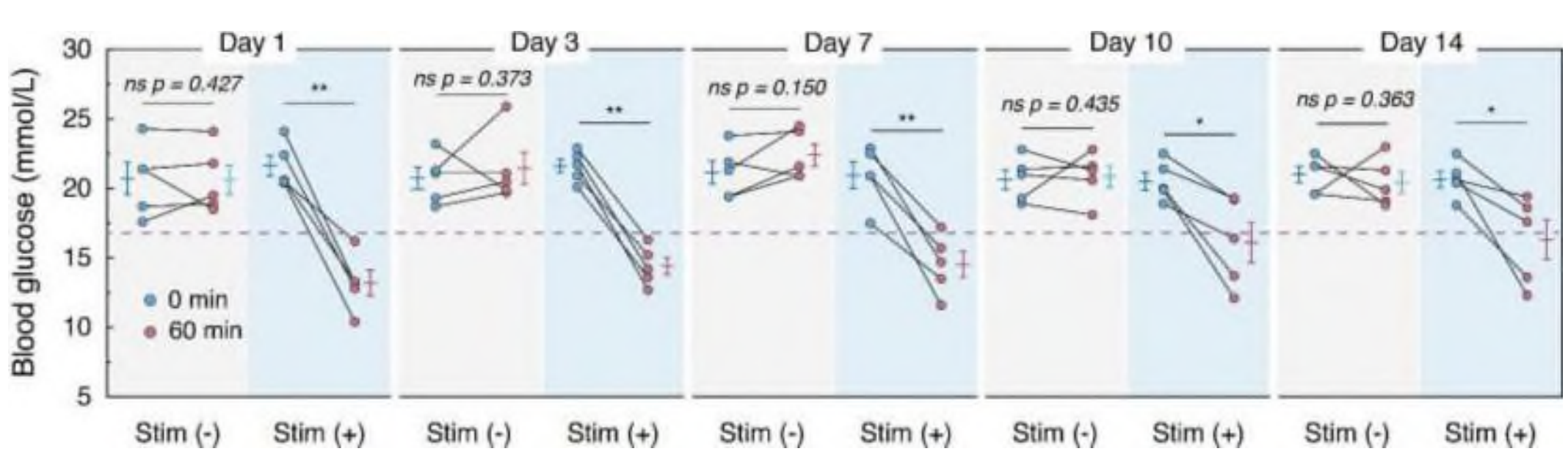

Fig. R11 Changes in blood glucose of individual treated mice before and after 60 min of optical stimulation at

day 1, 3, 7, 10 and 14 after TAHOF implantation. Data are presented as mean ± SE, with 5 mice per group. Statistical analyses were performed using two-tailed paired Student's *t*-tests. ns, not significant; *$p < 0.05$; **$p < 0.01$; ***$p < 0.001$.

Comment 13: In the conclusion section the authors should refrain from making exaggerated claim about "therapeutic potential" of their technology. While these devices are very useful to decipher and understand fundamental neurobiology of autonomic and somatic peripheral neural circuits, their therapeutic value is not demonstrated in any meaningful way in this work.

Answer: We thank the reviewer for this insightful comment and agree that the therapeutic potential of this technology should not be overstated within the current scope of the study. While our results demonstrate that TAHOFs enable stable optical interfacing with dynamic visceral organs and support functional optogenetic neuromodulation in vivo, we acknowledge that **direct therapeutic efficacy is not established here**. Accordingly, we have revised the Discussion and Conclusion sections to remove or moderate statements implying therapeutic relevance, and to more precisely position this work as an **application-driven, biointegrated optical interface** that addresses a key technical barrier, maintaining stable, on-target light delivery in mechanically dynamic environments, for investigating peripheral neural circuits. These revisions are highlighted in yellow in the manuscript.

Response to Reviewer #2

Comment 1: The article "Tissue-adhesive Hydrogel Optical Fiber for Peripheral OptogeneticNeuromodulation" reports a soft, biocompatible, and adhesive optical interface designed forstable light delivery to mechanically dynamic visceral organs. The device integrates apoly(HEMA) light-guiding core (ncore = 1.429) with a PAA-PVA bioadhesive cladding (ncladding= 1.342), yielding low propagation loss (0.529 dB/cm), robust interfacial adhesion (12 kPa),and high stretchability (176%). The authors further develop an integrated optogenetic platformand apply pulsed blue-light stimulation (470nm) to the pancreatic vagus nerve in freely movingChAT-ChR2 mice, achieving real-time glycemic regulation and sustained blood glucose control over three days in diabetic models. Overall, the study demonstrates how the synergistic integration of optical confinement, mechanical compliance, and tissue adhesion can advance spatiotemporally precise neuromodulation for anatomically complex biological systems. However, several minor issues require clarification before the manuscript can be considered for publication in Nature Communications.

Answer: Thank you for your positive and thoughtful assessment of our work. We appreciate your recognition of the significance of this study in developing a tissue-adhesive hydrogel optical interface for neuromodulation in mechanically dynamic visceral organs. Following your comments, we have carefully revised the manuscript to clarify the points you raised. A detailed point-by-point response is provided below, and all corresponding changes in the revised manuscript are highlighted in yellow

Comment 2: In the manuscript, the authors present several technical considerations relating to the use of 470 nm light, such as ChR2 activation, low propagation loss, and tissue-specific attenuation. However, these elements are currently scattered across the main text and supplementary materials, which weakens the clarity of the rationale behind optimising a blue-light system for deep-visceral applications where significant scattering occurs. To address this, the authors should compile these points into a consolidated 'design rationale' section that explicitly links the TAHOF architecture to the mitigation of blue-light-specific limitations.

Answer: We thank the reviewer for this valuable suggestion and agree that the rationale for using 470 nm light should be presented in a more unified and explicit manner. In this work, 470 nm illumination was chosen primarily because it matches the peak activation wavelength of ChR2, the most widely used excitatory opsin for peripheral optogenetics (e.g., *Nat. Methods* 20, 1802-1809 (**2023**), *Nat. Biotechnol.* 42, 892-904 (**2024**), *Nat. Commun.* 15, 3525 (**2024**)), while we fully acknowledge that blue light represents a challenging regime for deep-visceral applications due to strong tissue scattering and attenuation.

To clarify this point, we have reorganized these considerations into a consolidated "**design rationale**" section in the revised manuscript. In this section, we explicitly emphasize that the TAHOF architecture does not alter the intrinsic optical scattering properties of blue light, but instead mitigates delivery limitations through **(i) a core-cladding step-index structure that suppresses optical leakage, (ii) refractive-index engineering of the cladding to improve confinement, and (iii) tissue adhesion combined with mechanical compliance to minimize motion-induced misalignment and off-target illumination in dynamically deforming visceral organs**.

In addition, we have added new propagation-loss measurements at 589 nm and 644 nm (**Fig. R12**), showing that TAHOF maintains favorable optical performance beyond 470 nm, with even lower attenuation at longer wavelengths. These results further support that the device design is not inherently wavelength-restricted. All revisions have been incorporated into the revised manuscript (**Supplementary Fig. 5** and "*Design and fabrication of TAHOFs*" section), as highlighted in yellow.

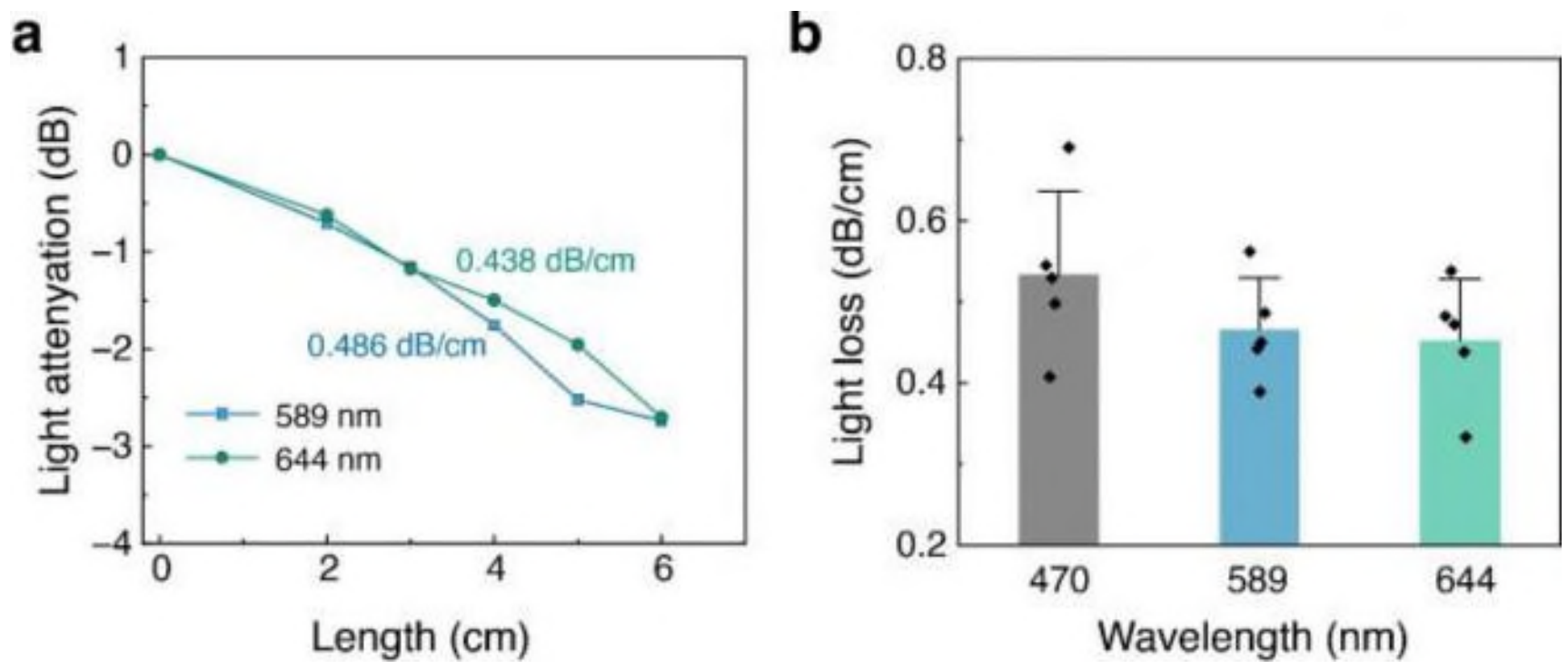

Fig. R12 Light propagation in TAHOF. a, Representative propagation loss of TAHOF hydrogel fibers at 589 nm and 644 nm, as measured by the cutback method. **b,** Quantitative summary of the propagation loss of TAHOF at λ = 470, 589, and 644 nm. Data are presented as mean ± SD, n = 5.

Comment 3: In the manuscript, the authors emphasize the broad applicability of the TAHOF platform to diverse morphologically dynamic organs and chronic neuromodulation therapies beyond the pancreatic use case. However, the current biological demonstrations are limited to a single opsin (ChR2), a single wavelength band (~470 nm), and a single physiological axis (pancreatic nerve–glucose regulation). To further strengthen the generality of the platform claim, it would be valuable to include at least one additional proof-of-concept-such as stimulation using a different opsin class, operation at a red-shifted wavelength, or a second organ/readout model-or to explicitly clarify the current scope and limitations of the biological demonstrations.

Answer: We thank the reviewer for this valuable comment. We agree that additional biological validation is important to support the general applicability of the TAHOF platform. In response, we addressed this point by incorporating a second proof-of-concept in a distinct mechanically dynamic peripheral organ, namely the stomach, to extend beyond the pancreatic neuromodulation paradigm.

Specifically, we performed **optogenetic stimulation experiments in the gastric vagal pathways**. Previous studies have shown that optogenetic and electrical manipulation of gastric vagal pathways can modulate gastric motility (*Nat. Commun.* 12, 157 (**2021**), *Neurogastroenterol Motil.* **2025**;37:e70030). Guided by this, we implanted the TAHOF on the lesser curvature of the stomach of ChAT-ChR2 mice, along the course of major gastric vagal branches (*Nat. Rev. Gastroenterol. Hepatol.* **2019**,16(2):89-105, *Am. J. Physiol. Gastrointest. Liver Physiol.* 284: G357-G366, **2003**), where it remained adhered to the target site and enabled stable optical stimulation in vivo (**Fig. R13a**). For functional readout, gastric slow-wave activity was recorded using one pair of Pt wires (diameter, 58 μm) inserted into the serosal surface of the proximal stomach, approximately 0.3-0.5 cm

away from the TAHOF implantation site.

As shown in **Fig. R13b-d**, optical stimulation (470 nm, 20 Hz, 5 ms) significantly decreased the average slow-wave amplitude from 0.107 mV to 0.048 mV (**p < 0.01), and reduced the average frequency from 6 cpm at baseline to 4.65 cpm (**p < 0.01). Both measures recovered after cessation of stimulation. Together, these results provide an additional biological demonstration that TAHOF enables functional optogenetic neuromodulation in a second, mechanically dynamic visceral organ, thereby strengthening the generality of the platform. We have added these new data in **Supplementary Fig. 34**, together with the corresponding experimental details in the Methods section "*Gastric optogenetic modulation via TAHOF*", and revised the related discussion accordingly, as highlighted in yellow.

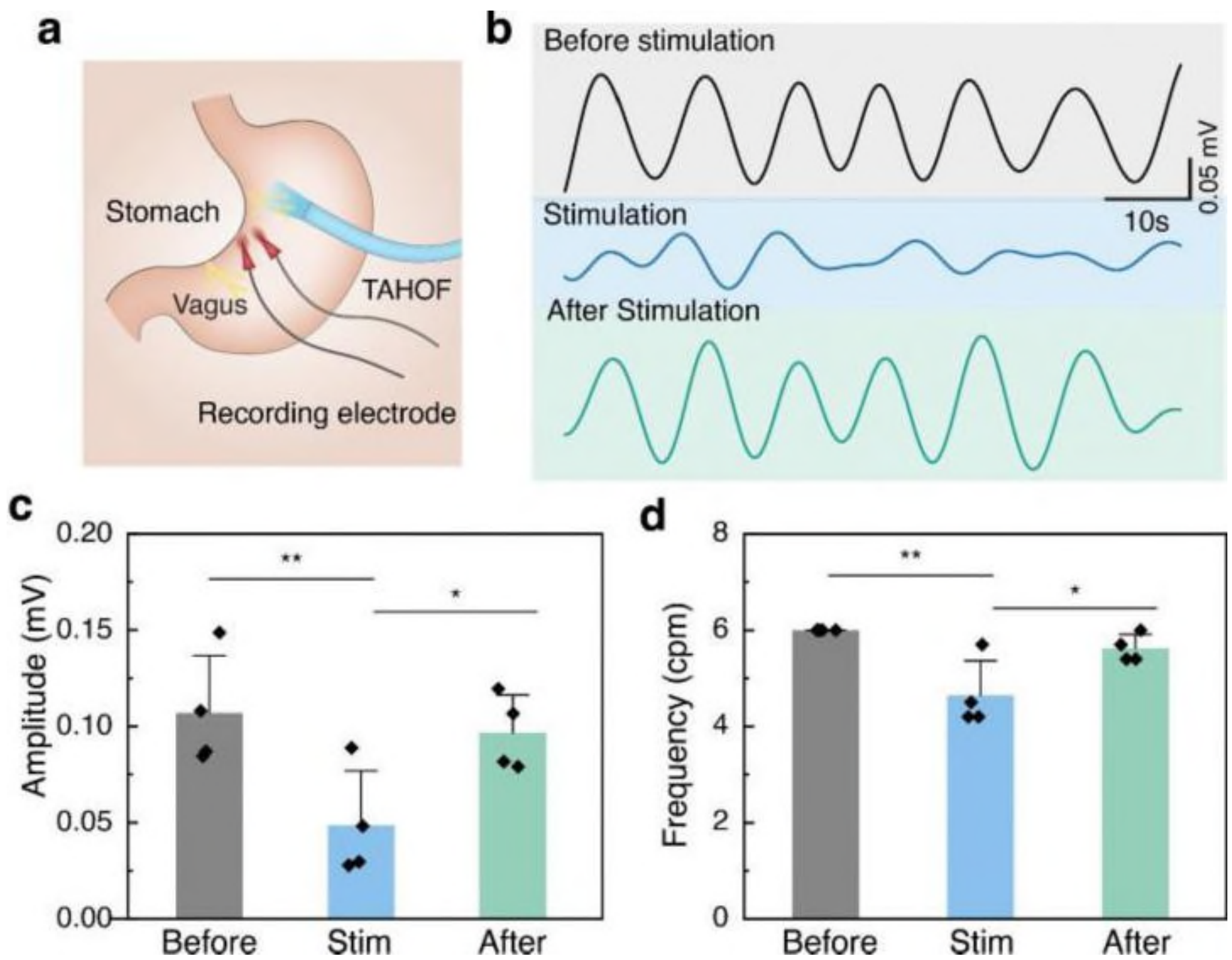

Fig.R13 Gastric optogenetic modulation via TAHOF. a, Schematic illustration of TAHOF implantation on the stomach for optical stimulation and electrophysiological recording of gastric slow waves in ChAT-ChR2 mice. **b,** Representative gastric slow wave recording during optical stimulation (470 nm, 20 Hz, 5 ms), together with the continuous wavelet transform of the filtered signals (frequency passband: 0.08-0.13 Hz). **c,d,** Quantitative analysis of gastric slow wave amplitude (**c**) and frequency (**d**) before, during, and after optical stimulation via TAHOF. Data are presented as mean ± SD, with 4 mice per group. Statistical significance and p-values were determined using two-sided unpaired t-test. *p < 0.05, ** p < 0.01, *** p < 0.001.

Comment 4: The waveguiding performance of TAHOFs relies critically on refractive-index contrast between the core, cladding, and surrounding tissue. While the manuscript notes that biological tissues can span a relatively broad refractive-index range (RI = 1.33–1.51), the numerical analysis appears to adopt a single representative value for the

surrounding tissue (ntissue = 1.500). It therefore remains unclear whether comparable confinement and leakage suppression are maintained across the full tissue refractive-index range encountered in vivo. A brief sensitivity analysis of waveguiding performance versus tissue refractive index, or ajustification that the chosen tissue represents a conservative worst case, would strengthen the robustness and generality of the optical design.

Answer: We thank the reviewer for this insightful suggestion. To address this concern, we performed an additional sensitivity analysis of waveguiding performance across the physiologically relevant tissue refractive-index range (n = 1.33-1.51). As shown in **Fig. R14**, FDTD simulations demonstrate that TAHOF consistently exhibits superior optical confinement and reduced leakage compared to the PHEMA core across all tested values. Over a propagation length of 400 μm, the leakage reduction is ~9% at n = 1.33 and increases to ~26% at n = 1.50, indicating progressively stronger relative performance under more optically challenging conditions.

We further clarify that n = 1.50 represents a conservative worst-case scenario, as increasing tissue refractive index reduces core-environment index contrast and therefore weakens confinement. The sustained performance under this condition confirms the robustness of the optical design across in vivo-relevant environments. We have added this sensitivity analysis and the corresponding discussion to the revised manuscript (**Supplementary Fig. 6**), as highlighted in yellow.

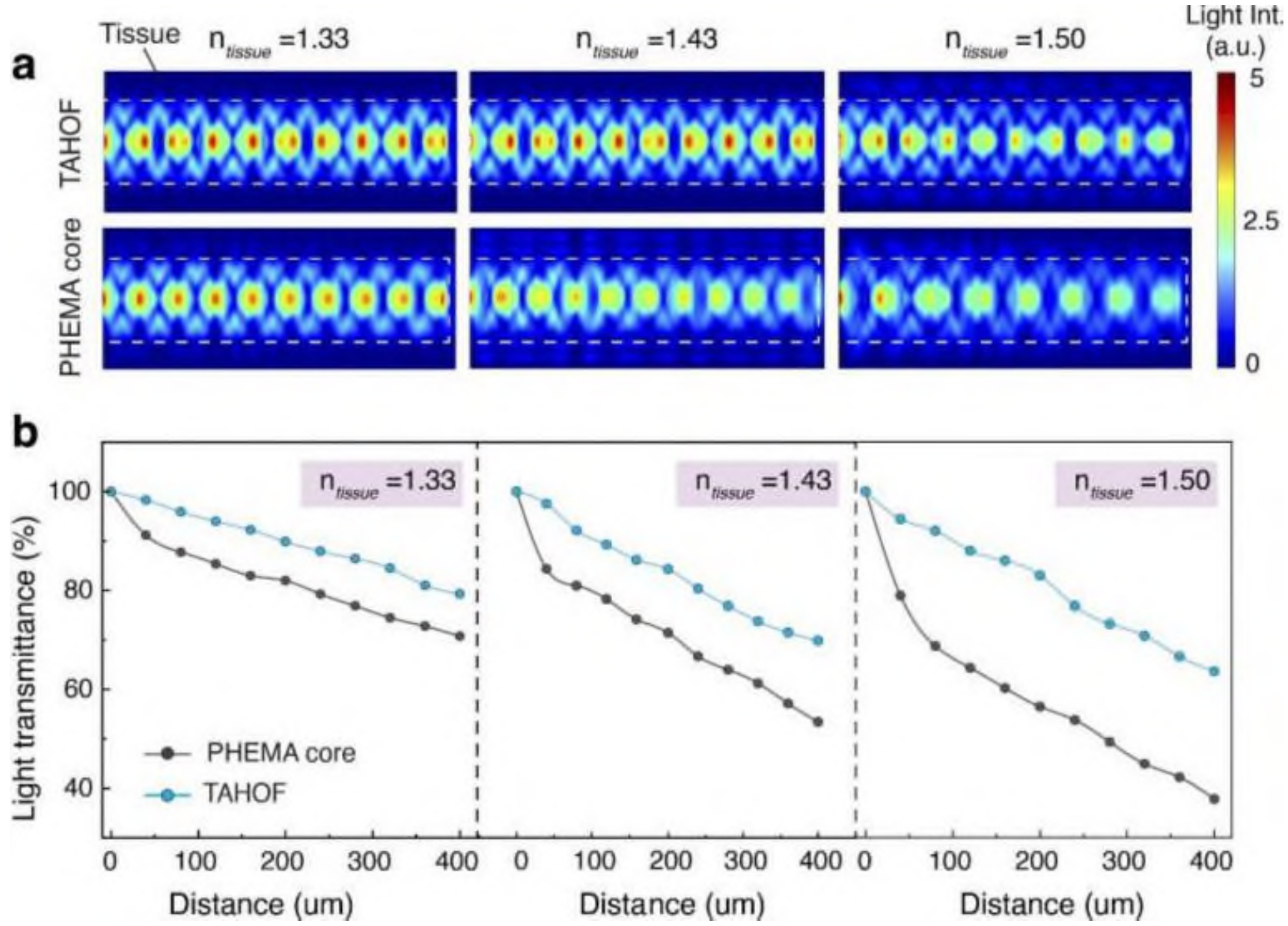

Fig. R14 FDTD simulation of optical transmission in TAHOF surrounded by biological tissues of different refractive indices. a, FDTD simulations of 470 nm light propagation showing enhanced optical confinement in TAHOF with adhesive cladding compared with the core-only fiber (PHEMA core) under surrounding refractive indices of 1.33, 1.43, and 1.50. The scaled structures used in the simulations were 5.5/1 μm for the TAHOF core/cladding and 6.5 μm for the core-only configuration. **b,** Quantitative analysis of axial light distribution along 400 μm fiber segments, showing improved transmission preservation in TAHOF compared with the core-only fiber

model under surrounding refractive indices of 1.33, 1.43, and 1.50.

Comment 5: While the study successfully demonstrates stable optical stimulation and glycemic regulation over 3 days, caution is warranted in extrapolating these findings to chronic or long-term applications. The current results are best interpreted as a short-term proof-of-concept; accordingly, the manuscript should either frame the outcomes explicitly as short-term achievements or extend the duration of the in vivo experiments to substantiate claims of longer-term applicability.

Answer: We thank the reviewer for this comment. We have revised the manuscript to explicitly frame the original 3-day glucose homeostasis experiment as a short-term proof-of-concept, rather than evidence of chronic or long-term applicability, and updated the relevant descriptions throughout the text accordingly.

To further address this concern, we performed an additional 14-day in vivo validation study using tail-vein blood sampling to minimize animal burden while maintaining optical stimulation via TAHOFs (**Fig. R10a-b**). Across all time points (days 1, 3, 7, 10, and 14), TAHOF-mediated stimulation (60 min per session) consistently induced significant reductions in blood glucose (**Fig. R10c-g and Fig. R11**), with decreases of ~6-9 mmol L^{-1} at early stages (days 1-7, $**p < 0.01$) and ~4-5 mmol L^{-1} at later stages (days 10 and 14, $**p < 0.01$). Although a gradual attenuation in response magnitude was observed, the glucose-lowering effect remained robust and statistically significant throughout the 14-day period, supporting sustained functional performance over an extended timeframe. All corresponding results have been incorporated into the revised manuscript (**Fig. 5f and Supplementary Fig. 33**) and highlighted in yellow.

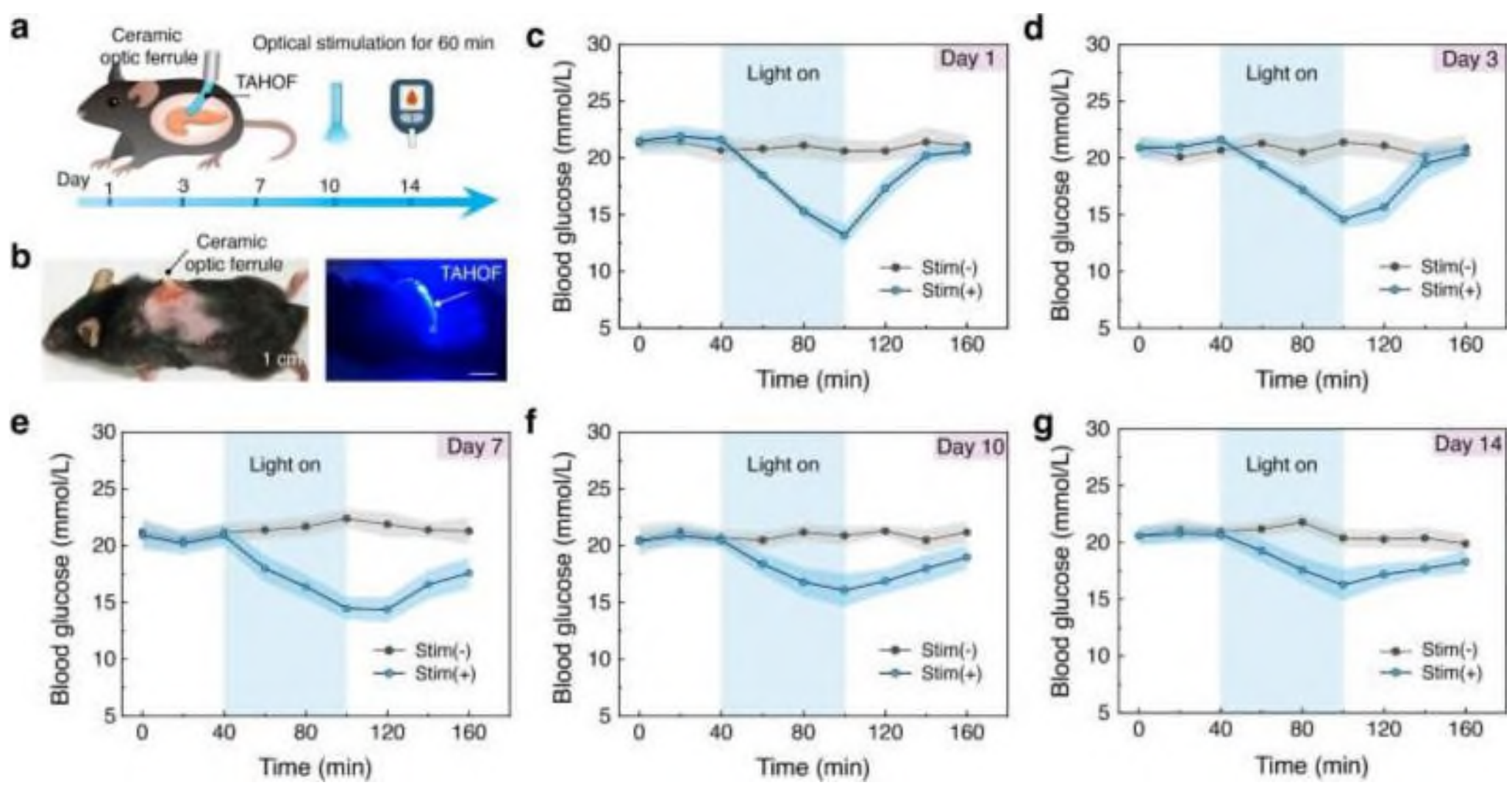

Fig. R10 Chronic optogenetic stimulation via implanted TAHOF in diabetic ChAT-ChR2 mice. a, Schematic of pancreatic TAHOF implantation and the chronic stimulation protocol. Optical stimulation was applied for 60 min at post-implantation day 1, 3, 7, 10, and 14. Blood glucose was measured every 20 min by tail-vein sampling using glucose test strips. **b,** Photograph of a TAHOF-implanted mouse. The fiber was fixed along the subcutaneous implantation path by the adhesive cladding, and the proximal end was coupled to a ceramic ferrule secured to the dorsal side of the mouse with dental cement. Scale bar, 1 cm. **c-g,** Blood glucose levels during 60-min optogenetic

stimulation (470 nm, 20 mW, 20 Hz, 5 ms) via TAHOF at post-implantation day 1 (**c**), day 3 (**d**), day 7 (**e**), day 10 (**f**), and day 14 (**g**). Mice implanted with TAHOF but without optical stimulation served as controls. Data are presented as mean ± SE, with 5 mice per group.

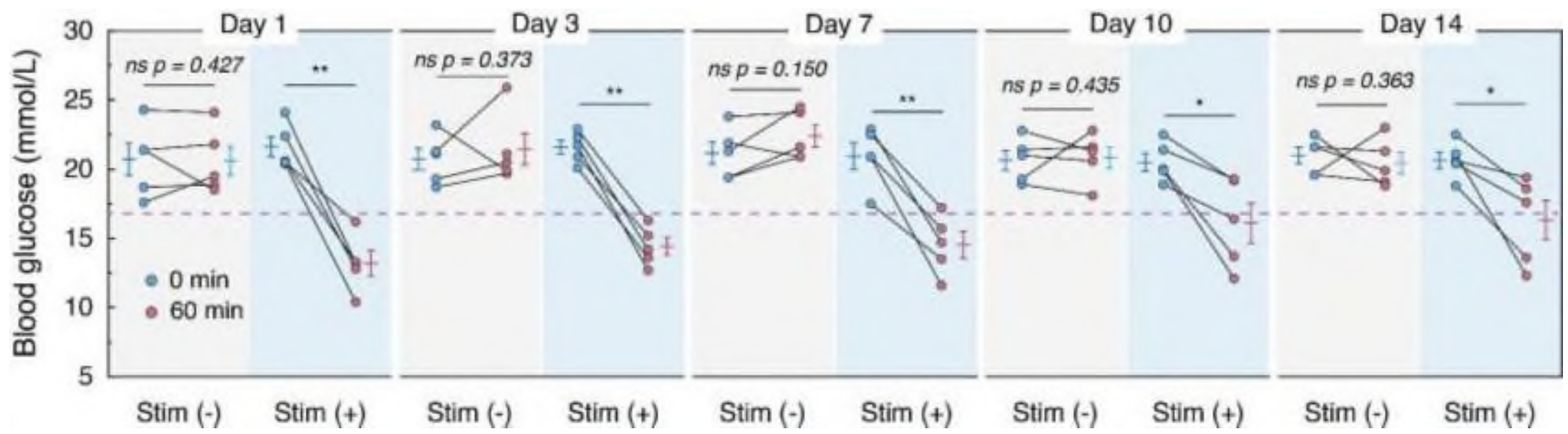

Fig. R11 Changes in blood glucose of individual treated mice before and after 60 min of optical stimulation at day 1, 3, 7, and 14 after TAHOF implantation. Data are presented as mean ± SE, with 5 mice per group. Statistical analyses were performed using two-tailed paired Student's *t*-tests. *$p < 0.05$; **$p < 0.01$; ***$p < 0.001$.

Comment 6: While the electrophysiological recordings in Fig. 4b demonstrate time-locked vagal nerve activity during optical stimulation, repeated light ON-OFF stimulation paradigms are needed to further establish a causal relationship between optical stimulation and neural activation.

Answer: We thank the reviewer for this helpful comment. As suggested, we implemented repeated light ON-OFF stimulation paradigms in ChR2-ChAT mice during electrophysiological recordings. As shown in **Fig. R15**, time-locked subdiaphragmatic vagus nerve activity was consistently observed during each light ON period (470 nm, 20 Hz, 5 ms), while no such activity was detected during the corresponding light OFF periods. This ON-OFF response pattern was reproducibly observed across four stimulation cycles and remained stable over a total recording duration of 600s. Following the reviewer's suggestion, we have replaced the original data in **Fig. 4b** with these new results and revised the related description in the manuscript (**Fig. 4c**), as highlighted in yellow.

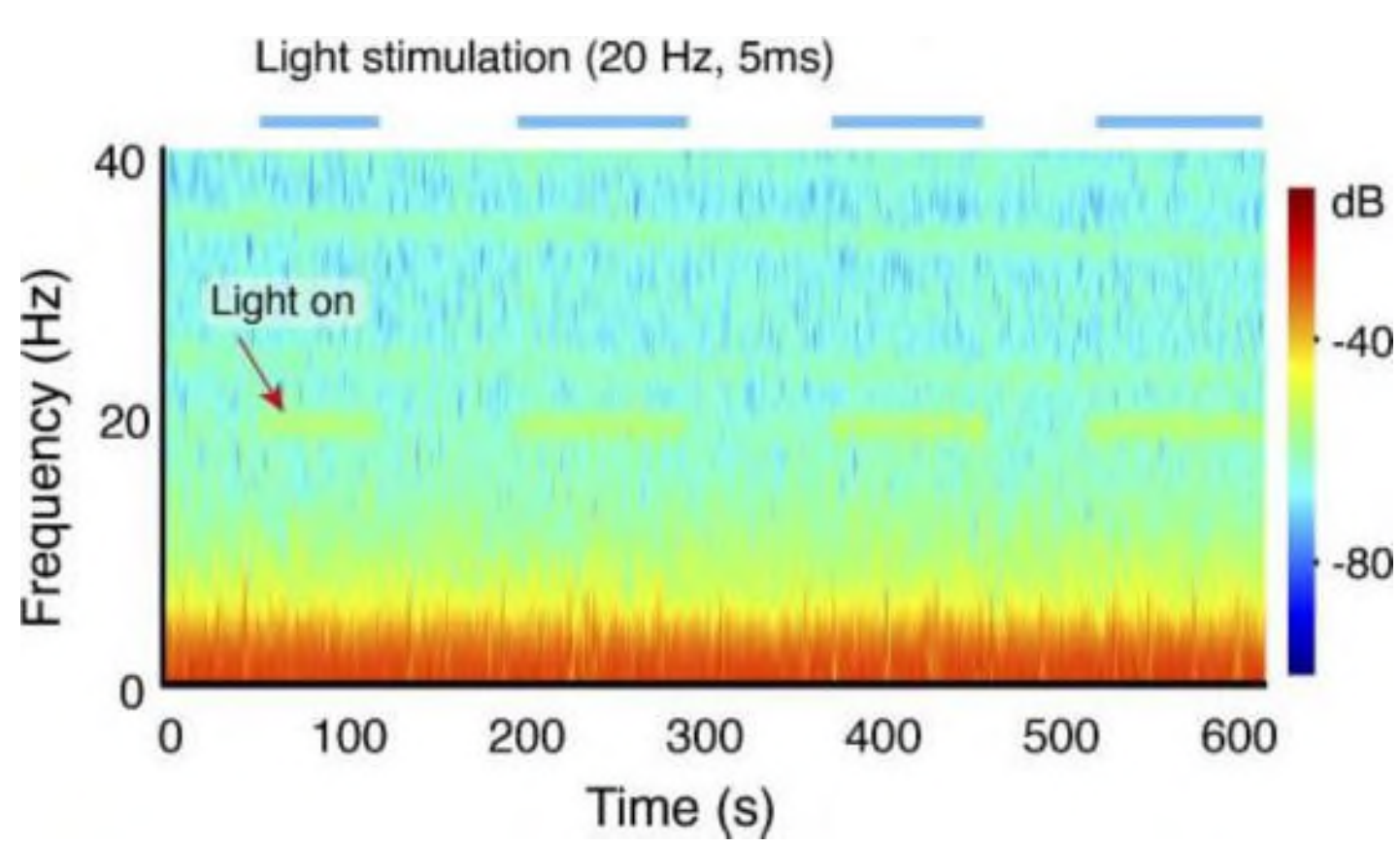

Fig. R15 Spectrogram of subdiaphragmatic vagus nerve local field potentials (LFPs) during four cycles of pulsed blue-light stimulation (470 nm, 20 Hz, 5 ms) delivered through the TAHOF.

Comment 7: Currently, quantitative lap-shear adhesion testing is limited to porcine skin, which represents a limitation given the emphasis on broad applicability in dynamic biological environments. To strengthen the generalizability of the TAHOF platform, simple lap-shear adhesion tests on multiple representative wet tissues should be included.

Answer: We thank the reviewer for this important comment. To further evaluate the generalizability of the adhesive performance across different wet tissues, we performed additional lap-shear adhesion tests on multiple representative biological tissues. As shown in **Fig. R16**, in addition to porcine skin, the adhesive cladding of TAHOF also exhibited measurable adhesion to intestine (13.1 ± 4.5 kPa), pork fillet (14.7 ± 1.4 kPa), heart (7.8 ± 1.9 kPa), and liver (4.8 ± 1.2 kPa). These results support that the adhesive interface of TAHOF is not limited to a single tissue type but can maintain adhesion across multiple representative wet biological tissues. We have added these new data and the corresponding discussion to the revised manuscript (**Supplementary Fig. 13**), as highlighted in yellow.

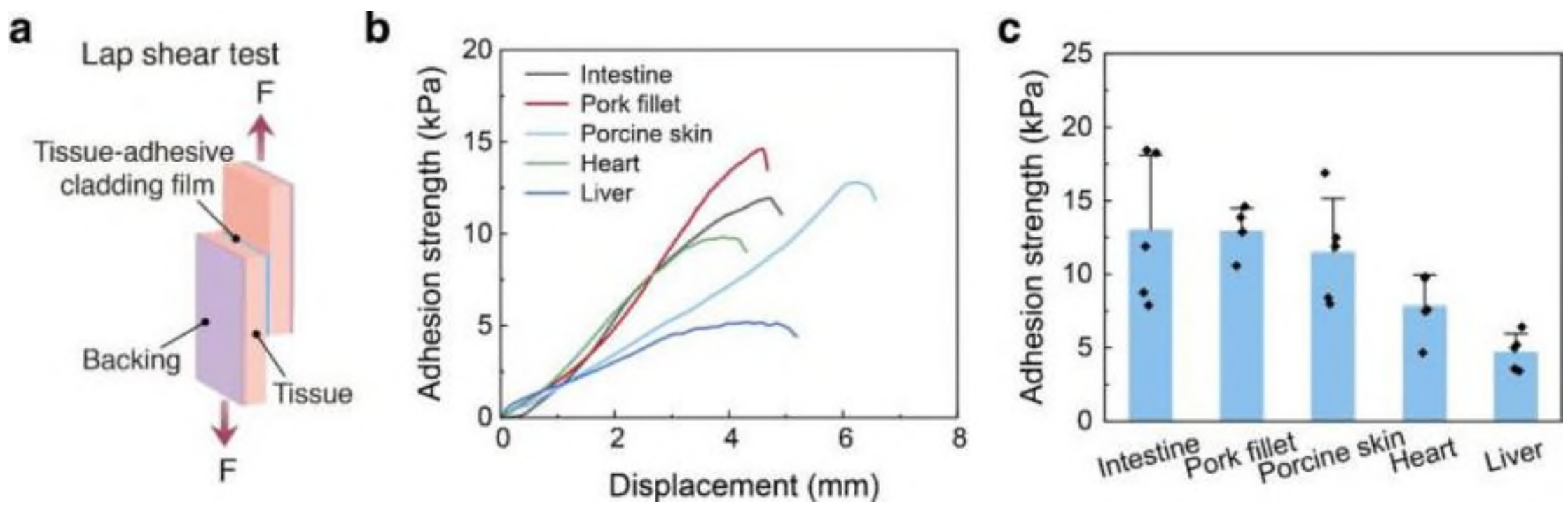

Fig. R16 Adhesion performance of the adhesive cladding layer of TAHOF. a, Schematic illustration of the lap-shear adhesion test. **b,c,** Representative lap-shear curves of shear stress versus displacement (**b**) and quantitative summary of the adhesion strength (**c**) of the adhesive cladding layer on various biological tissues, including porcine intestine, fillet, skin, heart, and liver. Data are presented as mean ± SD, n = 5.

Comment 8: Please clarify the figure citation "f Supplementary Fig. 16c and 17," on page 9, as the current notation is ambiguous.

Answer: We thank the reviewer for pointing out this ambiguity. The notation "*f Supplementary Fig.]6c and]7*" was incorrect. We have removed the extra "*f*" and clarified the corresponding figure citation in the revised manuscript. The correction is marked in the revised version (see **new Supplementary Figs. 22c** and **23**), as highlighted in yellow.

Response to Reviewer #3

Comment 1: This work proposes a novel hydrogel-based optical fiber with enhanced tissue-adhesive properties for optogenetic neuromodulation in the peripheral nervous system. The concept is extremely relevant for the application of optogenetics in regions of the body where significant movement of organs and tissues (e.g. peristalsis) might cause a disconnection of the probe from the tissue, leading to a misplacement of the stimulated area. The methodology of the investigation is sound, and the conclusions are supported by extensive experimental validation which is also accurately described in the main text and supplementary information. Overall,the work is novel, significant and well presented. Only a few minor comments should be, in my opinion, addressed:

Answer: Thank you for your positive and thoughtful assessment of our work. We appreciate your recognition of the relevance, novelty, and significance of this study, as well as your acknowledgment of the sound methodology and extensive experimental validation. We have carefully revised the manuscript in response to your comments. A detailed point-by-point response is provided below, and all corresponding changes in the revised manuscript are highlighted in yellow.

Comment 2: In Figure 1 and Supplementary Figure 6, the word "porcine" is misspelled;

Answer: We thank the reviewer for pointing out this error. We have corrected the misspelling by replacing *"Procine"* with *"porcine"* in **Figure 1b** and **original Supplementary Figure 6** (see new **Supplementary Figure 7**). In addition, we carefully checked the revised manuscript and corrected several other typographical errors, including "dehydrate TAHOFs sample" to "*dehydrated TAHOF sample*", "it swelling equilibrium state" to "*its swelling equilibrium state*". These corrections have been incorporated into the revised manuscript and are highlighted in yellow.

Comment 3: In the Introduction, in the paragraph following Figure 1, the authors refer to the cladding as "multifunctional". This term is typically used in the field for optical fibers that provide functions other than light delivery (e.g. drug delivery, electrophysiology), which does not seem to be the case in this work. I therefore suggest rephrasing this sentence;

Answer: We thank the reviewer for this helpful comment and agree that the term "multifunctional" may be misleading in this context, as the cladding does not introduce additional modalities beyond optical guidance. We have therefore revised the description to clarify that the cladding is designed to provide two coupled properties, tissue adhesion and refractive-index contrast for light confinement, within a single structural component. Accordingly, the sentence has been changed from "multifunctional cladding" to "*tissue-adhesive cladding*," and rewritten as "*Our TAHOF is composed of a poly(HEMA) optical core (n = 1.429) with a tissue-adhesive cladding (n = 1.342) that simultaneously provides tissue adhesion and the refractive-index contrast required for light confinement*," as updated in the *revised Introduction* and highlighted in yellow.

Comment 4: In the "Design and fabrication of TAHOFs" section, the discussion of the loss performance is made somewhat harder to read by the use of percentage values both for normalized transmission and transmission variation. As an example, the sentence "they maintained > 95%transmission integrity after 10,000 strain cycles" might be, at a first glance, interpreted as the fiber transmitting 95% of input light, which is inconsistent with the reported losses values. I suggest this part is rewritten in a clearer way, for example by providing absolute values of losses for the light propagation in tissue and clarifying the reference values for transmission variations;

Answer: We thank the reviewer for this comment. In the revised manuscript, we have clarified the distinction between absolute optical loss (reported in dB) and normalized transmission (reported relative to the initial prestress condition), and explicitly defined the corresponding reference points to avoid ambiguity in interpretation. This clarification has been incorporated throughout the "*Design and fabrication of TAHOFs*" section to ensure consistent reporting of optical performance metrics.

Accordingly, the sentence "they maintained > 95% transmission integrity after 10,000 strain cycles" has been revised to "*after 10,000 strain cycles, the normalized transmitted intensity remained above 95% of the initial value measured before cyclic loading, while the optical attenuation remained nearly constant, varying from 6.0 + 0.74 dB to 5.8 + 0.82 dB.*" These revisions improve clarity by separating relative transmission stability from absolute propagation loss, and have been updated in the manuscript (**Fig. 2g,h**) and highlighted in yellow.

Comment 5: In Supplementary Figure 6, the X-axis should be in linear scale;

Answer: We thank the reviewer for this comment. As shown in **Fig. R17**, we have revised the **X-axis** in **Supplementary Figure 6** to a linear scale. The corrected version is provided in the revised manuscript (see new **Supplementary Fig. 7**), as highlighted in yellow.

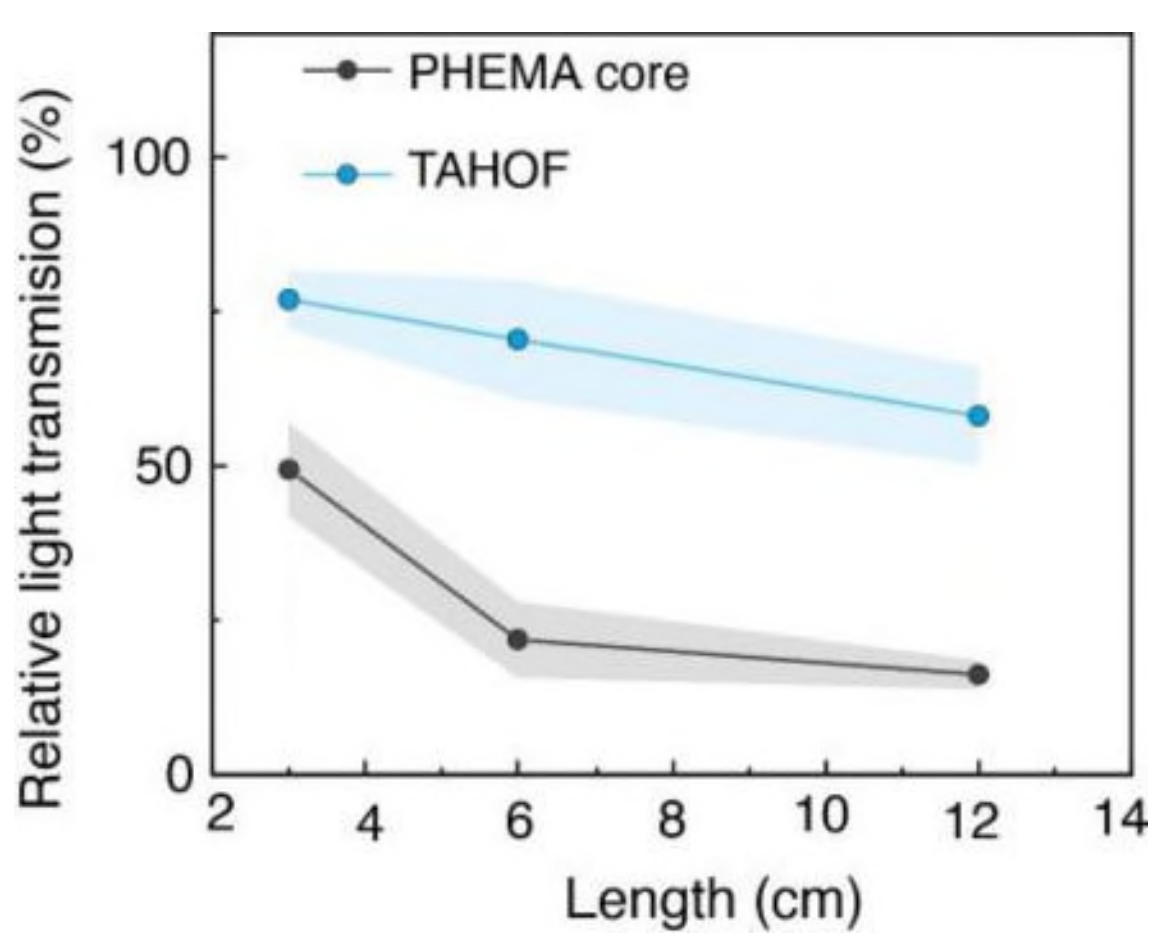

Fig. R17 Normalized light intensity as a function of transmission distance for TAHOF and PHEMA core hydrogel opticalfiber upon adhesion onto porcine tissues.

Comment 6: In Figure 2g, the attenuation in the fiber appears to be actually decreasing with increasing numbers of stretch cycles, which appears to be contrary to what is written in the text;

Answer: We thank the reviewer for pointing out this issue. The reviewer is correct that the attenuation in **Fig. 2g**

does not increase with increasing stretch cycle number. Rather, the data indicate that the optical performance remained stable during cyclic stretching. Although the mean attenuation showed a slight decrease after 10,000 cycles, this change was **not statistically significant** (***p* = 0.705**).

To avoid inconsistency between the figure and the text, we have revised the corresponding description in the manuscript to: *"after 10,000 strain cycles, the normalized transmitted intensity remained above 95% of the initial value measured before cyclic loading, while the optical attenuation remained nearly constant, varying from 6.0 + 0.74 dB to 5.8 + 0.82 dB."* We have also revised **Fig. 2g** and added the corresponding statistical comparison. These changes have been incorporated into the revised manuscript and are highlighted in yellow.

Comment 7: In page 5, the authors write "[...] hydrogel fibers without adhesive cladding are prone to dislocate under dynamic loads, leading to off-target illumination (Fig. 2h)". However, Fig 2h does not show this, but rather that a TAHOF does not dislocate;

Answer: We thank the reviewer for pointing out this oversight. The cited figure in this sentence was incorrect. This description refers to **Fig. 1b**, rather than **Fig. 2h**. We have corrected the figure citation accordingly in the revised manuscript, as highlighted in yellow.

Comment 8: In the "Biomechanical integration and strain-resilient optical delivery" section, the authors mention wavelength-dependent attenuation profiles. However, all attenuation measurements seem to be performed at 470 nm, rather than showing wavelength dependencies;

Answer: We thank the reviewer for this insightful comment. We agree that the original discussion of wavelength-dependent attenuation was based solely on 470 nm measurements and therefore required further experimental support. To address this point, we have additionally measured the propagation loss of TAHOFs at 589 nm and 644 nm.

As shown in **Fig. R18c,d**, the optical loss at 589 nm and 644 nm is 0.465 ± 0.057 dB/cm and 0.452 ± 0.067 dB/cm, respectively, both lower than that at 470 nm (0.534 ± 0.092 dB/cm). These results confirm a wavelength-dependent attenuation profile and indicate reduced propagation loss at longer wavelengths, consistent with decreased scattering and improved transmission. We have incorporated these new data and revised the corresponding description in the manuscript accordingly (**Supplementary Fig. 5**), as highlighted in yellow.

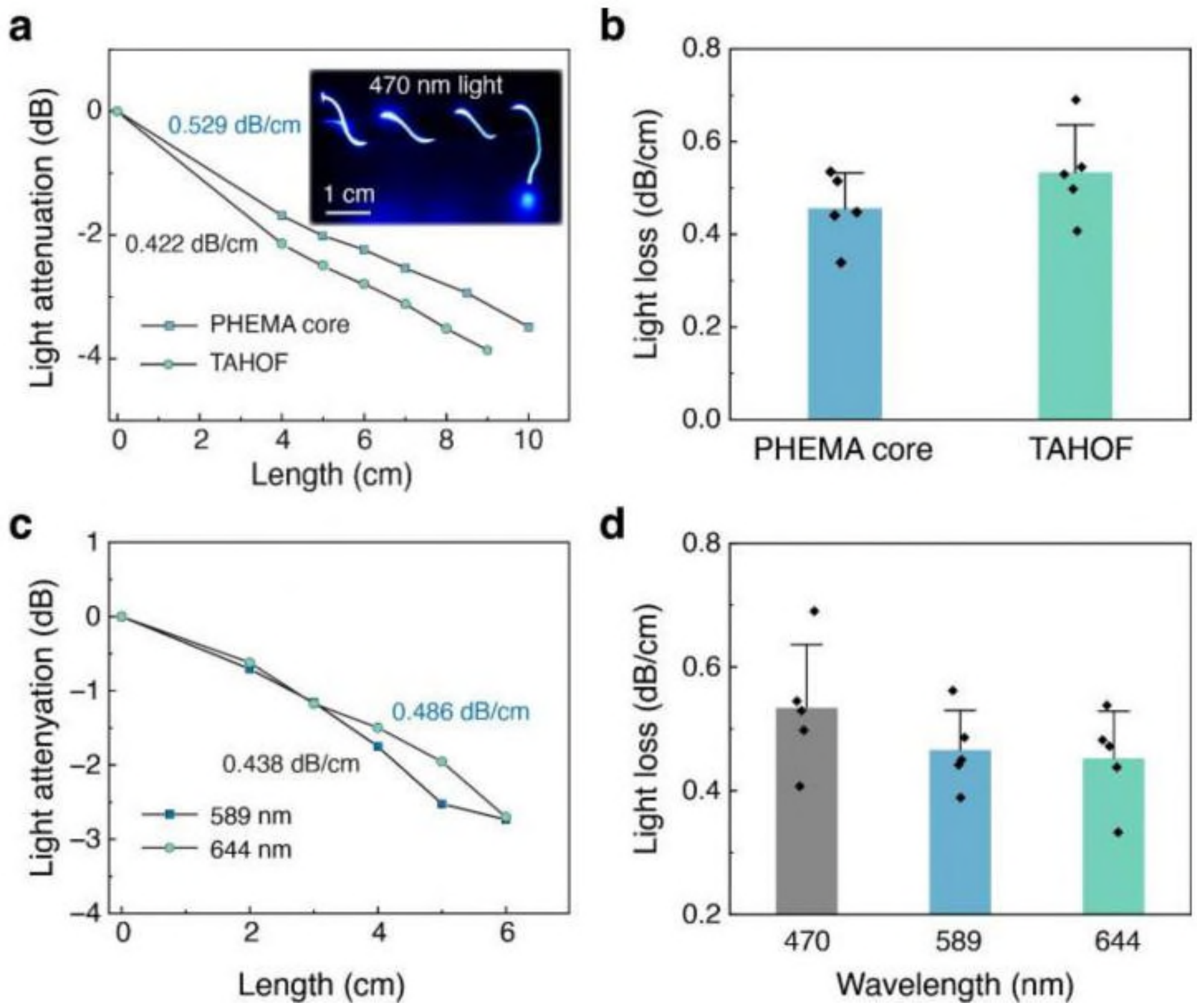

Fig. R18 Light propagation in TAHOF. a Light propagation in TAHOF. a, Representative propagation loss of TAHOF and PHEMA core fibers at **470 nm**, measured by the cutback method. Inset, maintained light guiding of TAHOF during mechanical deformation (rod-wrapping configuration). Scale bar, 1 cm. **b,** Quantitative summary of the propagation loss of TAHOF and HOF measured by the cutback method. **c,** Representative propagation loss of TAHOF hydrogel fibers at 589 nm and 644 nm, measured by the cutback method. **d,** Quantitative summary of the propagation loss of TAHOF at **λ = 470, 589, and 644 nm.** Data are presented as mean ± SD, n = 5.

Comment 9: In the "Neuromodulatory control of blood glucose via the vagus-pancreas axis enabled by TAHOFs" section, the authors have implanted an electrode array adjacent to the optical neuromodulation point to measure optically-induced LFPs. Have the authors considered or somehow mitigated the possibility of photoelectric artifacts affecting the LFP.

Answer: We thank the reviewer for this important comment. To address the possibility of photoelectric artifacts in the LFP recordings, we performed additional control experiments using **ChR2(-) littermate mice** implanted with the same TAHOF-cuff electrode configuration. In these experiments, the TAHOF tip was positioned ~1 mm away from the recording cuff to minimize direct optical exposure to the electrode interface (**Fig. R19a**).

Importantly, in **ChR2(-) mice**, spectrogram analysis showed no detectable increase in spectral power or time-locked signal components during optical stimulation (**Fig. R19b**). In contrast, robust, stimulation-locked vagal responses were observed only in **ChAT-ChR2 mice** under identical conditions (**Fig. R19c**). These results confirm that the recorded LFP signals are attributable to optogenetic activation of vagal fibers rather than photoelectric

artifacts. We have incorporated these control experiments and the corresponding discussion into the revised manuscript (**Fig. 4b-c and Supplementary Fig. 20**), as highlighted in yellow.

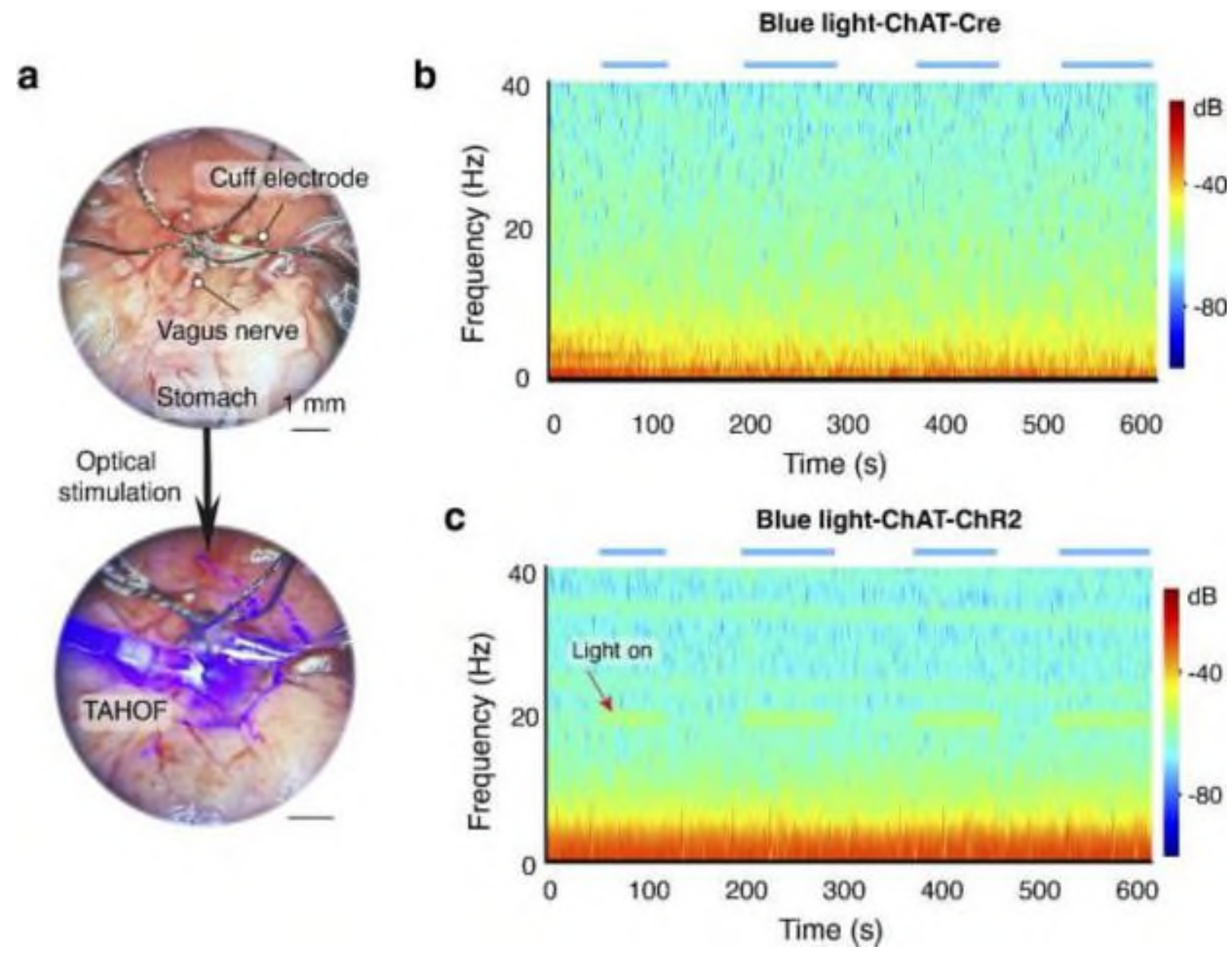

Fig. R19 Electrophysiological recording of subdiaphragmatic vagus nerve during optical stimulation via TAHOF. a, Photograph of the experimental setup for electrophysiological recording with a cuff electrode and optical stimulation delivered by the TAHOF. Scale bar, 1 mm. **b,c,** Spectrograms of local field potentials (LFPs) recorded from the subdiaphragmatic vagus nerve in ChR2(-) littermate mice (**b**) and ChAT-ChR2 mice (**c**) during pulsed blue-light stimulation (470 nm, 20 Hz, 5 ms) delivered through the TAHOF.

RE: Our point-by-point response to the reviewers' comments (NCOMMS-26-003310A).

Response to Reviewer #1

Comment 1: In the Introduction, the authors should clarify that the hydrogel chemistries are based on previously published literature, and that the primary contribution of this work lies in their application toward tool development.

Answer: We thank the reviewer for this comment. Following this suggestion, we have revised the **Introduction** to clarify that the hydrogel chemistries used in this work are based on previously reported literature. Specifically, we revised *"Here, we report a tissue-adhesive hydrogel optical fiber (TAHOF) that enables on-target light delivery for peripheral optogenetic neuromodulation of visceral nerve in freely moving animals."* to *"Building on recent advances in hydrogel optical fiber design and hydrogel chemistries, we report a tissue-adhesive hydrogel optical fiber (TAHOF) as a light-delivery tool for peripheral optogenetics, enabling on-target optogenetic neuromodulation of visceral nerves in freely moving animals."* This revision is highlighted in yellow.

Comment 2: Standard error or standard deviation should be reported for all experimentally measured and quantified parameters mentioned in the text. For example, values such as "low propagation loss (0.529 ± xx dB/cm)" should include the appropriate measure of variability.

Answer: We thank the reviewer for this important comment. Following this comment, we have added the corresponding standard deviation (SD) or standard error (SE) values for experimentally measured and quantified parameters throughout the revised text, as highlighted in yellow. For example, in the **Abstract**, we have revised the relevant values to include the corresponding variability, including "*poly(HEMA) light-guiding core (ncore = 1.429 ± 0.004) with a bioadhesive cladding (ncladding = 1.342 ± 0.003)*", "*low propagation loss (0.534 ± 0.092 dB/cm)*", and "*robust tissue integration (11.5 ± 1.8 kPa)*".

Comment 3: When the device is implanted in mice, the ferrule will remain externalized. Over time, the hydrogel optical fiber threaded into the ferrule may dehydrate, which could lead to poorer coupling with the silica coupling fiber, increased optical loss, and possible mechanical failure. The authors should address this concern and discuss a potential mitigation strategy.

Answer: We thank the reviewer for this valuable comment. To mitigate dehydration of the hydrogel fiber at the externalized ferrule interface, we maintained a locally hydrated environment in the ferrule when optical stimulation was not being applied. Specifically, **the ceramic ferrule was covered with its matching protective cap, and the ferrule cavity was filled with PBS (~300-400 μL) to maintain hydration.** The PBS was replenished every 12 h to reduce dehydration of the hydrogel fiber and thereby minimize potential coupling deterioration, increased optical loss, and mechanical instability at the fiber-ferrule interface (**Fig. R1**). Following

this comment, we have added the corresponding experimental details to the revised **Methods**, "*In vivo implantation of TAHOFs*" as highlighted in yellow.

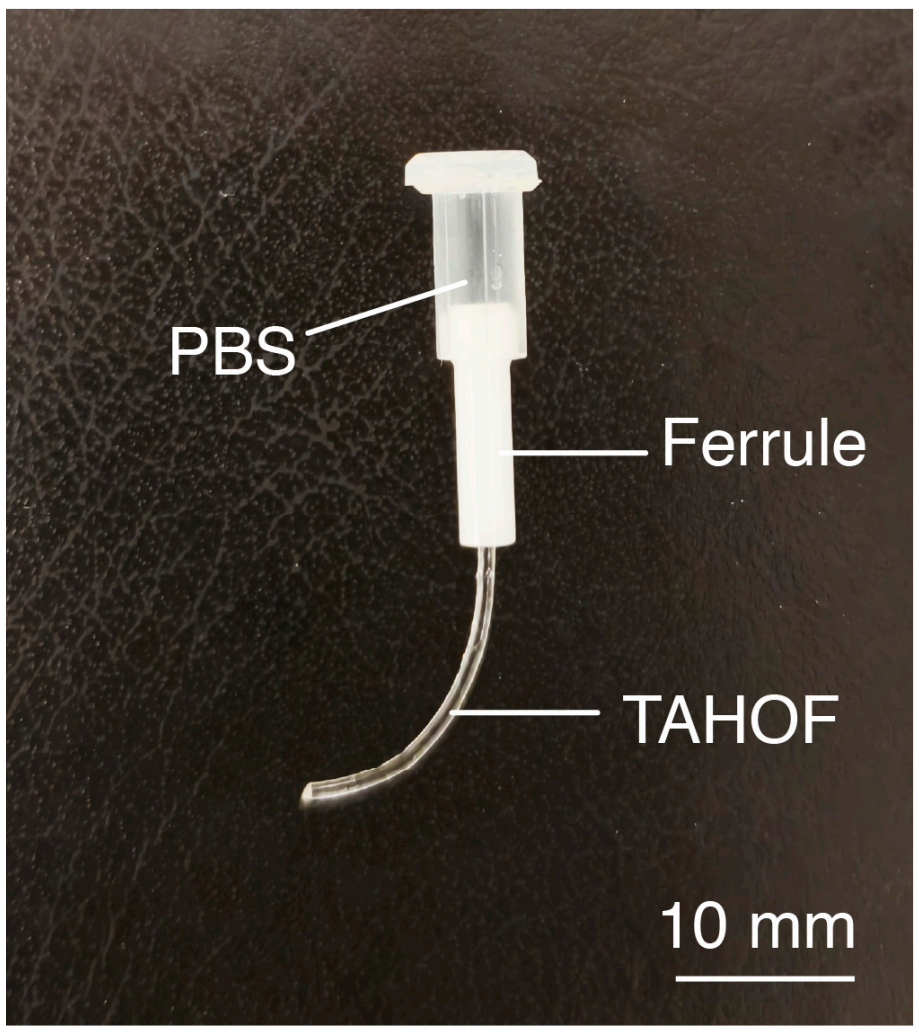

Fig.R1 Photograph of the ceramic ferrule covered with its matching protective cap, with the ferrule cavity filled with PBS to maintain hydration of the TAHOF. Scale bar, 10 mm.

Comment 4: The authors use transgenic mice that express ChR2 in all ChAT+ neurons. It is important to note that ChAT vagal neurons innervate not only the pancreas, but also many other organs in the abdominal cavity, including the stomach, duodenum, ileum, and hepatic portal vein. This point is particularly relevant because the authors do not use a light-blocking cladding along the length of the fiber, which creates the possibility of light leakage into surrounding tissues. In several images, substantial light leakage along the fiber length is clearly visible. This could result in unintended optogenetic stimulation of other abdominal organs. The authors should address this issue and provide a mitigation strategy, either in the Discussion section or as a supplemental note in the Supporting Information.

Answer: We thank the reviewer for this helpful comment. We recognize that ChAT-positive vagal neurons innervate multiple abdominal organs and that, without a fully light-blocking outer layer, side leakage along the fiber path could in principle raise the possibility of unintended optical stimulation. We have therefore added further discussion of this point in the revised manuscript.

The TAHOF uses a **step-index core-cladding architecture to confine light within the waveguide through total internal reflection**, with a propagation loss of 0.534 ± 0.092 dB/cm at 470 nm. Although visible light emission can be observed along the fiber path in **Supplementary Fig. 7b** and **Supplementary Fig. 33a**, this appearance is amplified by dark-environment imaging, camera overexposure, and tissue scattering. More

importantly, any side leakage is distributed along the propagation path rather than concentrated at a single stimulation site. Supplementary Fig. 7c shows that after propagation over 3 cm on porcine tissue, the remaining transmission of the TAHOF was 77 ± 4.8% of that measured in air. Based on the measured propagation loss in air, a 30 mW input corresponds to an output of approximately 20.7 mW after 3 cm of propagation, and to approximately 15.9 mW after further accounting for the tissue-associated transmission reduction. Thus, **the optical loss along the fiber length is distributed over the 3 cm path and is further attenuated by tissue scattering and absorption**, whereas optical activation in our experiments was achieved by delivering 20 mW specifically at the fiber tip adjacent to the intrapancreatic ganglia. Therefore, side leakage along the fiber length is much less likely to induce unintended optogenetic activation than the localized light delivered at the tip.

The current mitigation strategy relies on optical confinement by the cladding and stable tissue adhesion, which together help maintain on-target delivery and reduce motion-induced misalignment. We have also noted in the revised manuscript that future optimization could incorporate an additional light-blocking outer layer (e.g., light-absorbing graphene oxide, Nat. Methods 20, 1802-1809 (2023)) to further suppress side leakage along the fiber path. These revisions have been incorporated into the revised manuscript (**Supplementary Fig. 7**) and are highlighted in yellow.

Comment 5: In Fig. R5a, the title indicates “ChR2-Cre,” whereas the caption refers to “ChR2(-) littermates,” which would imply control mice that do not express ChR2 after breeding with the Ai27 line. Please ensure consistency.

Answer: We thank the reviewer for this comment. As shown in **Fig. R2**, we have corrected this inconsistency and unified the labeling in the revised figure. To avoid misunderstanding, “ChAT-Cre” has been revised to “ChAT-ChR2(-)”, and the ChAT-ChR2 mice are now labeled as “ChAT-ChR2(+)” in revised **Supplementary Fig. 20**, ensuring consistency with the corresponding caption and the intended control group designation. This revision is highlighted in yellow.

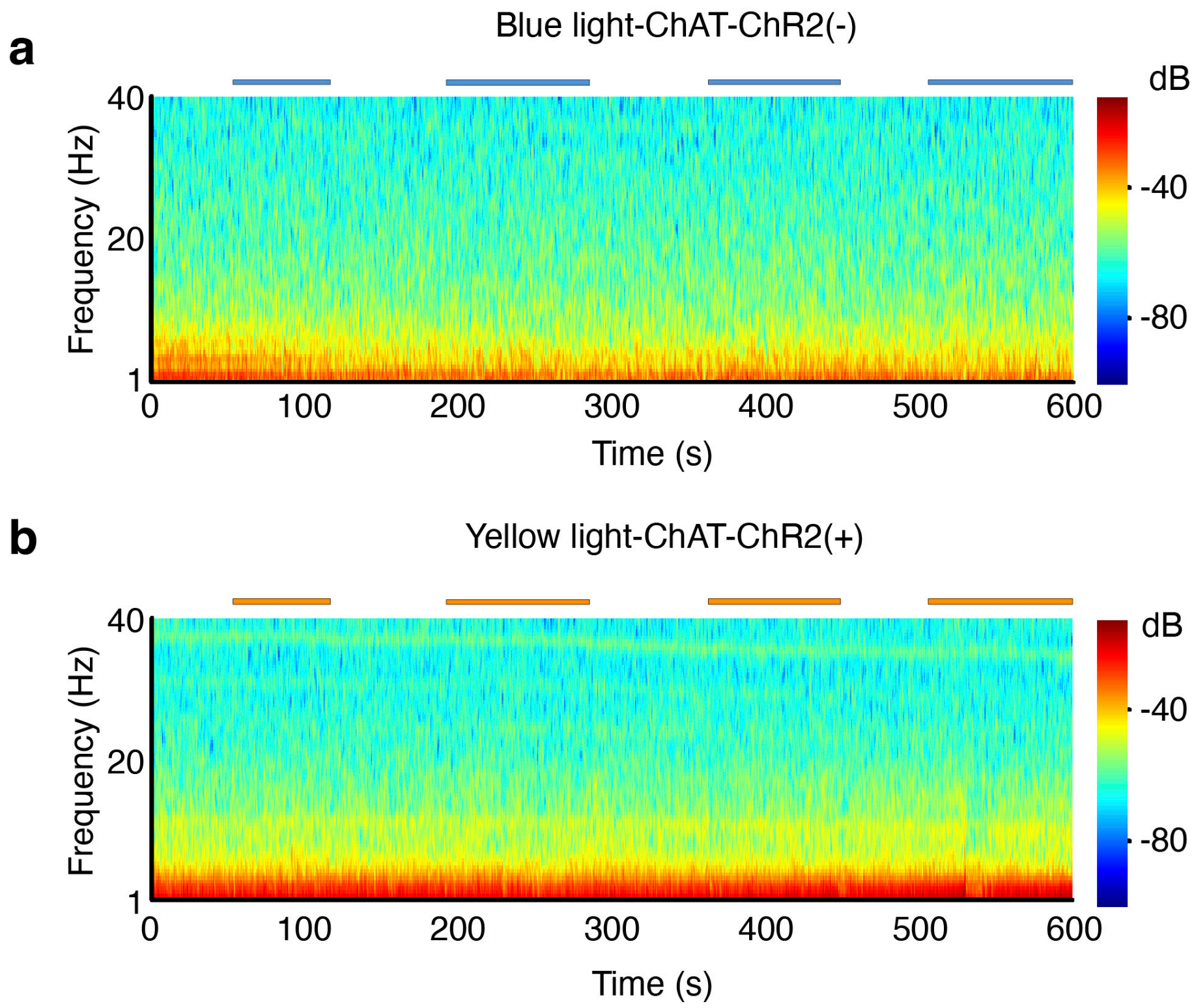

Fig. R2 Spectrograms of local field potentials (LFPs) recorded from the subdiaphragmatic vagus nerve during optical stimulation. a, Spectrogram of LFPs recorded with a cuff electrode in ChR2-negative mice (ChAT-ChR2(-)) during pulsed blue-light stimulation (470 nm, 20 Hz, 5 ms) delivered through the TAHOF. **b,** Spectrogram of LFPs recorded with a cuff electrode in ChAT-ChR2(+) mice during pulsed yellow-light stimulation (589 nm, 20 Hz, 5 ms) delivered through the TAHOF.
